# Species Delimitation in the Bark Louse Genus *Neostenopsocus* Liang & Liu, 2024 (Psocodea: Stenopsocidae) Based on DNA Barcoding

**DOI:** 10.3390/insects16111147

**Published:** 2025-11-09

**Authors:** Feiyang Liang, Wei Zeng, Sheng Li, Xingyue Liu

**Affiliations:** 1 Key Laboratory of Economic Crops Genetic Improvement and Integrated Utilization, School of Life and Health Sciences, Hunan University of Science and Technology, Xiangtan 411201, China; 24010901005@mail.hnust.edu.cn; 2 State Key Laboratory of Animal Biodiversity Conservation and Integrated Pest Management, Institute of Zoology, Chinese Academy of Sciences, Beijing 100101, China; lisheng9202@163.com; 3 Department of Entomology, China Agricultural University, Beijing 100193, China

**Keywords:** COI gene, genetic distance, Psocoptera, psocids, species identification

## Abstract

The family Stenopsocidae exhibits a rich biodiversity in China, yet species identification based on morphological characters remains problematic due to insufficient study and absent molecular data. DNA barcoding has been widely employed for rapid species identification in insects, particularly through standardized COI gene sequencing. In this study, we conducted species delimitation for *Neostenopsocus* in China based on the morphological characters and DNA barcodes, comprising 20 morphological species with 110 barcodes. We redescribed 13 species from China and proposed 39 new synonyms. This study supports the applicability of DNA barcoding for species identification in *Neostenopsocus*. The K2P distance between the morphological species of *Neostenopsocus* ranges from 0.0051 to 0.2040, with most exceeding 0.10. The maximum intraspecific divergence reaches 0.0778 in *N. nepalensis*. The external morphology of *Neostenopsocus* shows considerable structural uniformity, and the genitalia provide limited diagnostic value for species identification. Instead, the marking patterns of the head and forewing are considered to be useful for species identification. Therefore, delimiting closely related species demands an integrated taxonomy approach that combines extensive geographical sampling, detailed morphological analysis, and nuclear DNA data.

## Simple Summary

Bark lice are common in terrestrial ecosystems, yet the poor taxonomic study of them renders species identification difficult. In this study, we investigated the species identification of the bark louse genus *Neostenopsocus* using DNA barcodes (675 bp fragment of the mitochondrial COI gene). We examined newly collected specimens and the type materials of some valid species of *Neostenopsocus*, redescribing 13 valid species and proposing 39 new synonyms. A total of 110 sequences of *Neostenopsocus* species were obtained from newly collected specimens and public data. The results show that the COI barcode is sufficient to distinguish most *Neostenopsocus* species, whereas more molecular marks and distribution data are necessary to identify the closely similar species.

## 1. Introduction

The order Psocodea includes three suborders: Trogiomorpha, Troctomorpha, and Psocomorpha [1,2]. Psocomorpha is the largest suborder, which represents a large group of free-living psocids, and generally lives in trees. Stenopsocidae is a family of Psocodea belonging to the suborder Psocomorpha, in the infraorder Caeciliusetae, including more than 190 valid species, and divided into four genera: *Graphopsocus* Kolbe, 1880; *Malostenopsocus* Li, 1992; *Neostenopsocus* Liang & Liu, 2024; and *Stenopsocus* Hagen, 1866 [3,4,5,6,7]. Liang & Liu [5] estimated the phylogenetic relationships within the family Stenopsocidae based on their mitochondrial genes and nuclear 18S rRNA, and established the genus *Neostenopsocus*, which includes 115 species previously placed in *Stenopsocus*. This genus can be distinguished from the other genera of Stenopsocidae by a forewing with glabrous CuP, a labrum with distal styli, and an abdomen with three ventral vesicles [5]. *Neostenopsocus* is extremely rich in East and Southeast Asia, with the highest diversity in China. Li [2] described 97 species of *Stenopsocus* from China, all of which were transferred to *Neostenopsocus* by Liang & Liu [5]. *Neostenopsocus* shows considerable structural uniformity, and it is not easy to identify the species based on the original descriptions by Li [2]. Saville [8] examined 100 specimens of *Stenopsocus immaculatus* from Britain, and considered the external characters of this species to be variable. These previous studies have implied that species identification based solely on morphological characters remains challenging due to insufficient morphological studies of *Neostenopsocus*.

In recent studies, taxonomists have generally followed an integrative taxonomic approach that combines DNA barcodes and morphological characters for insects’ species delimitation [9,10,11,12]. DNA barcoding is a popular tool for species identification in the fields of ecology, evolution, and conservation [13,14]. Hebert et al. [15] defined the concept of DNA barcode, and considered the mitochondrial COI gene could serve as the core of a global bioidentification system for animals. For insects, the COI provides an ideal species marker owing to its infrequent possession of introns, simple alignment, limited exposure to recombination, and the availability of robust primer sites [16]. The Barcode of Life Data System (BOLD) is one of the largest online DNA barcode databases and provides an integrated bioinformatics platform that supports all phases of the analytical pathway, from specimen collection to a tightly validated barcode library [17,18]. For the family Stenopsocidae, BOLD contains 2813 barcodes, predominantly from *Graphopsocus cruciatus* (1856 barcodes), with most of the specimens collected from Europe and America [19]. However, BOLD has released only a few DNA barcodes of Stenopsocidae specimens from Asia, while this family shows a rich biodiversity in this region. Liang et al. [20] employed DNA barcoding to identify *Stenopsocus* species from Taiwan Island, yet the limited specimens’ size and narrow geographic coverage hindered its utility as a robust molecular identification for this genus.

In the present study, we present an integrative taxonomy of the genus *Neostenopsocus*, with the redescription of 13 valid species of *Neostenopsocus* and the proposition of 39 new synonyms by using the DNA barcodes (675 bp region of the mitochondrial COI gene) and morphological features for species delimitation.

## 2. Materials and Methods

*DNA extraction, PCR amplification, and sequencing.* The total genomic DNA was extracted from the thoracic muscle tissues of single specimens using a TIANamp Genomic DNA Kit (TIANGEN Biotech Co., Beijing, China). The polymerase chain reactions (PCRs) were performed using an NEB Long Taq DNA polymerase system (New England BioLabs, Ipswich, MA, USA). A 675 bp fragment of the COI gene was amplified with the universal primer pair LCO1490 (5′-GGTCAACAAATCATAAAGATATTGG-3′) and HCO2198 (5′-TAAACTTCAGGGTGACCAAAAAATCA-3′) [21]. The thermal cycling program consisted of an initial denaturation at 95 °C for 30 s, followed by 40 cycles of 95 °C denaturation for 10 s, 45 °C annealing for 50 s, a 65 °C extension for 1 min, and a final extension at 65 °C for 10 min. The PCR products were sequenced bidirectionally using a BigDye^®^ Terminator v3.1 Cycle Sequencing Kit (Applied Biosystems, 850 Lincoln Centre Drive, Foster City, CA, USA).

*Sequence analysis and tree reconstruction.* Consensus sequences were created with the program Consed 15.0 [22] using sequence data from both DNA strands. The sequences were double-checked by eye and aligned with ClustalW [23], using MEGA 11 [24] as the sequence editor. We constructed a set of COI datasets from 122 barcodes of 23 Stenopsocidae species (Alignment S1) used for the molecular study. A neighbor-joining (NJ) tree was performed in MEGA 11 using the Kimura-2-Parameter (K2P) model [25] with 1000 bootstrap replicates, based on the above COI dataset. All the samples used in the present study are shown in Table S1.

*Phylogenetic Analyses.* Maximum likelihood (ML) analysis was performed using the IQ-Tree web server(http://iqtree.cibiv.univie.ac.at/, accessed on 24 October 2025) [26] with an ultrafast bootstrap approximation approach with 1000 replicates. A phylogenetic analysis was conducted using MrBayes v3.2 [27], run with a total of 4 million generations, sampled every 100 generations, and cut off with 25% of the sampled trees. We estimated the best partitioning scheme and model for our dataset with PartitionFinder 2 [28]. The concatenated dataset was partitioned into six subsets and the HKY+I+G model was selected.

*Species delimitation.* The pre-delimitated species were identified based on the external color characteristics as described by Liang et al. [20], i.e., markings on the head and forewings, antennae coloration, and genital segment pigmentation. We identified 17 species from China by these features. The genetic distances between and within the species were calculated using the Kimura 2 Parameter (K2P) model with MEGA 11. Subsequently, three molecular species delimitation methods were used for the production of species hypotheses, as follows: (1) Automatic Barcode Gap Discovery (ABGD) [29]; (2) Assemble Species by Automatic Partitioning (ASAP) [30]; and (3) the Poisson tree processes (PTP) method [31]. ABGD and ASPS, the two distance-based analyses, were performed using the K2P model for the above COI dataset with the outgroup taxa excluded. The Poisson tree processes (PTP) is a phylogeny-based approach. The ML tree was generated by IQ-tree web server and was imported into the bPTP web server (https://species.h-its.org/ptp/, accessed on 24 October 2025) to delimit the species.

*Taxonomy study.* The specimens were stored in 95% ethanol (Aladdin Scientific Corp., Shanghai, China) after collection and all were deposited in the Entomological Museum of China Agricultural University (CAU) and the Zoological Specimen Collection of Hunan University of Science and Technology (HNUST). The genitalic preparations were made by clearing the apex of the abdomen in a cold, saturated NaOH solution (Aladdin Scientific Corp., Shanghai, China) for 6 h. After rinsing off the NaOH with water, the apex of the abdomen was transferred to glycerin (Aladdin Scientific Corp., Shanghai, China) for further dissection and examination. The type materials were deposited in the Entomological Museum of China Agricultural University (CAU), except for the type specimens of *Stenopsocus externus*, which were deposited in the Museum of Comparative Zoology (MCZ). The terminology follows Yoshizawa [32] and Liang & Liu [5]. The abbreviations for the parts measured are as follows: f1-fn, flagellomeres 1-n; d, transverse diameters of right compound eye; IO, minimum distance between compound eyes; FWL, length of forewings; FWW, width of forewings; HWL, length of hind wings; HWW, width of hind wings; t1 and t2, first and second tarsomere of right hind leg.

## 3. Results

### 3.1. Species Delimitation

**Figure 1:**
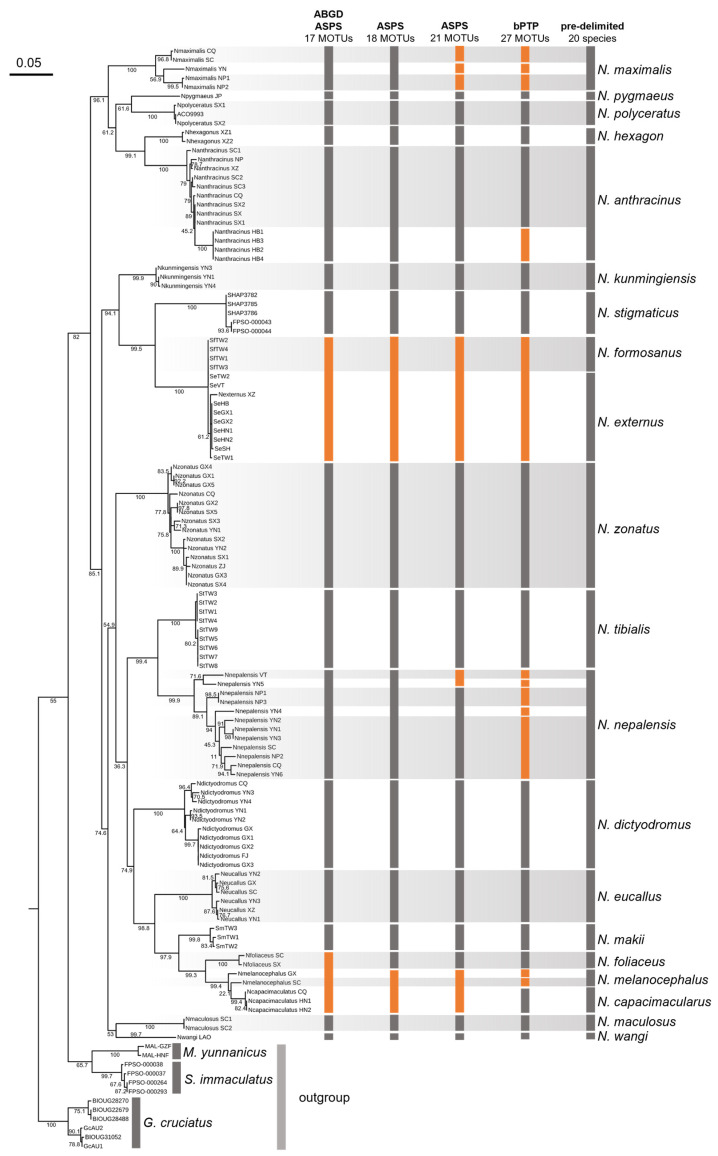
ML tree of *Neostenopsocus* based on COI sequence and molecular species delimitation results. Results of molecular species delimitation analyses by ABGD, ASAP, bPTP, and morphological delimitation are reported through vertical bars on the right side of the tree. Numbers at nodes indicate ML bootstrap values. The colored vertical bars indicate species delimitated by the different approaches. The grey bar indicates congruent results between the molecular and morphological identifications, the orange bars indicate differences in the results from different species delimitation methods.

The intergeneric phylogenetic relationships within the family Stenopsocidae and the monophyly of *Neostenopsocus* were supported by Liang and Liu [5] through phylogenetic analyses based on the mitochondrial genomes and the nuclear 18S rRNA gene. Based on the morphological diagnostic characteristics of *Neostenopsocys* provided by Liang and Liu [5], we could confirm that all individuals were identified as *Neostenopsocus* in this study. In this study, we used three approaches to construct phylogenetic trees: maximum likelihood (ML) (Figure 1), Bayesian inference (BI) (Figure S1), and neighbor joining (NJ) (Figure S2). These trees showed phylogenetic relationships among the four genera of Stenopsocidae that are consistent with those of Liang & Liu [5], and recovered all *Neostenopsocus* individuals, forming a monophyletic clade. The low support value at the root of the phylogenetic trees may be attributed to the limited resolution of the COI DNA barcodes, combined with the substantially smaller sample sizes for the genera *Graphopsocus* and *Malostenopsocus* compared to that for *Neostenopsocus*.

Due to the simplicity of the genital structure in *Neostenopsocus*, which provides few diagnostic features, we used the external color characteristics for species identification. These comprised markings on the head and forewings, antennae coloration, and genital segment pigmentation. Integrating these features with the geographical distribution information enabled the identification of 17 species from China. Among them, detailed descriptions of four species from Taiwan can be found in Liang et al. [20]. The specific diagnostic characteristics and redescriptions for the remaining 13 species are provided in the following section, 3.2 Systematics.

By using MEGA 11, the K2P distance between the pre-delimited species of *Neostenopsocus* was found to range from 0.0051 to 0.2040, with the smallest distance being between *N. externus* and *N. formosanus* (0.0051) and the largest distance being between *N. anthracinus* and *N. stigmaticus* (0.2040) (Table S2). The interspecific K2P distances were typically more than 0.10. Five pairwise COI distances fell below 0.10, i.e., *N. externus* and *N. formosanus* (0.0051), *N. capacimacularus* and *N. melanocephalus* (0.0290), *N. capacimacularus* and *N. foliaceus* (0.0803), *N. melanocephalus* and *N. foliaceus* (0.0849), and *N. melanocephalus* and *N. makii* (0.0917). The intraspecific variation ranged from 0 (*N. capacimacularus* (n = 3), *N. maculosus* (n = 2), and *N. formosanus* (n = 3)) to 0.0489 (*N. nepalensis* (n = 12)), overlapping the lower bound of interspecific divergence (Table S3). The zero intraspecific genetic distances observed in several species are likely attributable to insufficient sampling. The maximal intraspecific distance (0.0778) occurred between NnepalensisYN5 and NnepalensisNP2 (Table S4).

The ASPS analysis produced ten best partitions, with the ASPS scores ranging from 6.00 to 17.50. According to the standard (the lower the score, the better the partition), three solutions stood out: 21 MOTUs with a score of 6.00 (threshold distance: 0.0410), 17 MOTUs with a score of 6.50 (threshold distance: 0.0915), and 18 MOTUs with a score of 7.50 (threshold distance: 0.0600) (Figure 1). A barcoding gap occurred at 0.06–0.07. It is noteworthy that the ASAP analysis assigned *N. externus* and *N. formosanus* individuals to a single MOTU. Due to the unavailability of additional *Neostenopsocus* specimens from Taiwan Island, we adopted the published COI barcodes from Liang et al. [20] and provisionally maintained their distinct species-level designations pending further data. We argued that the result of 21 MOTUs partition should be rejected due to over-splitting, because the morphologically uniform *N. maximalis* individuals were divided into three separate MOTUs in this result. The distinction between the 17 and 18 MOTU schemes lies in the treatment of *N. melanocephalus*, *N. capacimacularus*, and *N. foliaceus*: the former unites all specimens of these taxa in a single MOTU, whereas the latter segregates them into two MOTUs (Figure 1). Additionally, from the results of ABGD analyses, 17 MOTUs were identified as the optimal species partition (Figure 1), a result fully consistent with the 17 MOTUs inferred by ASAP. Besides the species mentioned above, the remaining predelimited species of *Neostenopsocus* were supported by the ASAP and ABGD analyses.

Compared to the results from the ABGD and ASAP, the bPTP delimitated many more MOTUs, with 27 MOTUs for *Neostenopsocus* (Figure 1). In the PTP delimitation, some individuals with highly similar morphological characteristics but from different geographical populations were classified into different MOTUs, such as individuals from the pre-delimited *N. maximalis* and *N. anthracinus*. Therefore, the PTP model may have over-split the species of *Neostenopsocus* in this study. Similar cases are found for the species delimitation of Psylloidea (Hemiptera) [33] and Chrysopidae (Neuroptera) [34]. This situation regarding the results of PTP model may be due to incomplete lineage sorting, which has been documented in previous studies [35,36,37].

Therefore, we examined numerous individuals of the genus *Neostenopsocus,* including recently collected specimens and type materials. Integrating the morphological evidence, distributional data, and DNA barcodes, we propose 39 new subjective synonyms: 13 synonyms of *N. anthracinus*, 1 synonym of *N. capacimacularus*, 3 synonyms of *N. dictyodromus*, 1 synonym of *N. eucallus*, 2 synonyms of *N. externus*, 1 synonym of *N. kunmingiensis*, 8 synonyms of *N. maculosus*, and 10 synonyms of *N. zonatus*.

### 3.2. Systematics

**Genus *Neostenopsocus* Liang & Liu, 2024**

*Neostenopsocus* Liang & Liu, 2024 [5]: 439. Type species: Stenopsocus externus Banks, 1937 [38]: 259, by original designation.

Diagnosis: See Liang & Liu [5].

Remarks: See Liang & Liu [5].

Distribution: Oriental, Palearctic, and Australian regions.

**Figure 2:**
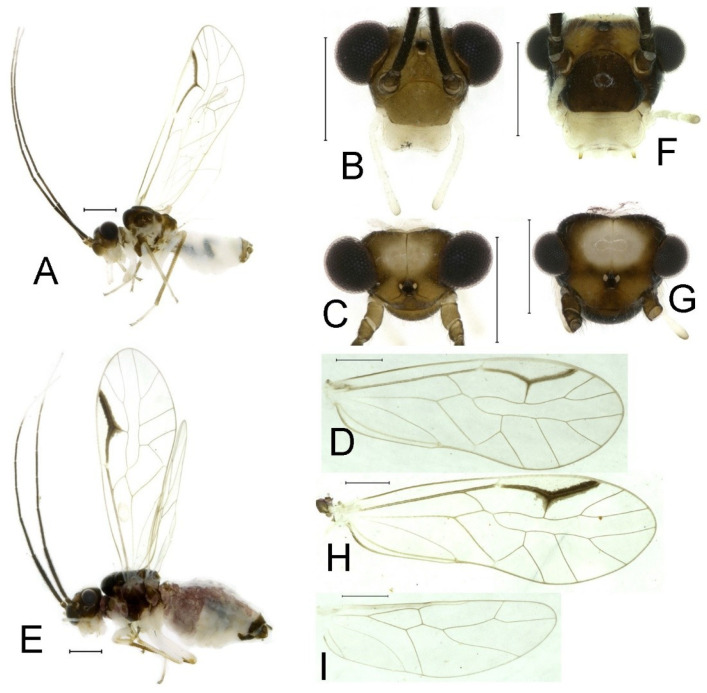
Photos of *Neostenopsocus anthracinus*: (**A**) male, habitus; (**B**) male, frontal view of head; (**C**) male, dorsal view of head; (**D**) male, forewing; (**E**) female, habitus; (**F**) female, frontal view of head; (**G**) female, dorsal view of head; (**H**) female, forewing; (**I**) female, hind wing. Scale bar = 0.5 mm.

**Figure 3:**
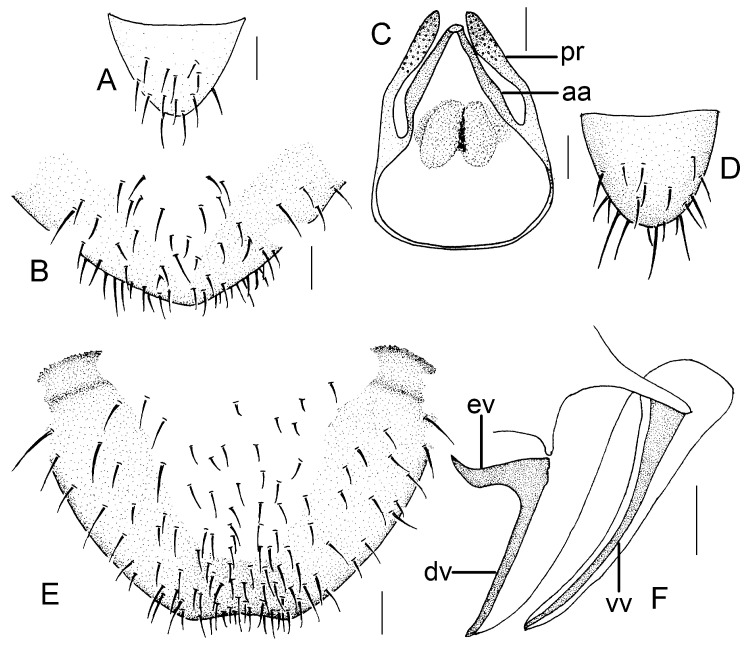
Drawing of *Neostenopsocus anthracinus:* (**A**) male, epiproct; (**B**) male, hypandrium; (**C**) male, endophallus; (**D**) female, epiproct; (**E**) female, subgenital plate; (**F**) female, gonapophyses. aa: aedeagal arch; pr: paramere; dv: dorsal valve; ev: external valve; vv: ventral valve. Scale bar = 0.05 mm.

**(1) *Neostenopsocus anthracinus* (Li, 1989) (**Figure 2 and Figure 3**)**

*Stenopsocus anthracinus* Li, 1989 [39]: 36. Type locality: China (Shaanxi: Zhenba).

*Stenopsocus angustifurcus* Li, 2002 [2]: 658. Type locality: China (Guizhou: Dushan). **syn. nov.**

*Stenopsocus biconvexus* Li, 1997 [40]: 428. Type locality: China (Hubei: Xingshan). **syn. nov.**

*Stenopsocus bipunctatus* Li, 2002 [2]: 637. Type locality: China (Yunnan: Longling). **syn. nov.**

*Stenopsocus cassideus* Li, 1997 [41]: 313. Type locality: China (Hunan: Sangzhi). **syn. nov.**

*Stenopsocus dichospilus* Li, 2002 [2]: 657. Type locality: China (Anhui: Huangshan). **syn. nov.**

*Stenopsocus flavifrons* Li, 1989 [39]: 35. Type locality: China (Shaanxi: Liuba). **syn. nov.**

*Stenopsocus frontalis* Li, 1989 [39]: 38. Type locality: China (Shaanxi: Zhenba). **syn. nov.**

*Stenopsocus fulivertex* Li, 2002 [2]: 647. Type locality: China (Hubei: Jiugongshan). **syn. nov.**

*Stenopsocus parviforficatus* Li, 2002 [2]: 649. Type locality: China (Ningxia: Liupanshan). **syn. nov.**

*Stenopsocus podorphus* Li, 1997 [40]: 435. Type locality: China (Hubei: Xingshan). **syn. nov.**

*Stenopsocus qianipullus* Li, 2005 [42]: 92. Type locality: China (Guizhou: Dashahe). **syn. nov.**

*Stenopsocus symipsarous* Li, 2002 [2]: 656. Type locality: China (Guizhou: Guiyang). **syn. nov.**

**Diagnosis.** This species exhibits noticeable sexual dimorphism in body coloration. The females are characterized by the antenna, with 9–13 antennomeres whitish, forewing pterostigma with brown marking extending to mid of r-rs, and black–brown genital segments. In contrast, males display paler overall pigmentation, uniformly brown antennae, and forewing pterostigma with brown marking not extending to r-rs.

**Redescription.** Adult male: Body (Figure 2A) length 2.42 mm, length from postclypeus to wing tip 4.42 mm. IO: 0.29 mm; d: 0.27 mm; IO/d = 1.07; f1: 0.76 mm; f2: 0.59 mm; f3: 0.51 mm; FWL: 3.65 mm; FWW: 1.34 mm; HWL: 2.69 mm; HWW: 0.88 mm; t1: 0.42 mm; t2: 0.15 mm.

Color (in alcohol): Head (Figure 2B,C) yellowish brown, vertex with a yellowish area. Antenna blackish brown. Mouthparts mostly yellowish with blackish-brown postclypeus, apex of maxillary palpus pale brown, remaining segments of maxillary palpus whitish. Thorax dark brown. Leg femur mostly yellowish, tibia brown. Abdomen yellowish white, genital segments brown.

Forewing (Figure 2D) transparent. R dark brown. Posterior margin of pterostigma with narrow brown stripe not extending along r-rs. Hindwing immaculate.

Genital segments strongly sclerotized. Epiproct (Figure 3A) subtriangular. Paraproct with 22 trichobothria. Hypandrium (Figure 3B) sclerotized strongly. Endophallus (Figure 3C) strongly sclerotized, external parameres robust, with some punctures on apex, and not exceeding apex of aedeagal arch; aedeagal arch narrow.

Adult female: Body (Figure 2E) length 2.68 mm, length from postclypeus to wing tip 5.02 mm. IO: 0.44 mm; d: 0.14 mm; IO/d = 3.14; f1: 0.89 mm; f2: 0.65 mm; f3: 0.45 mm; FWL: 4.08 mm; FWW: 1.41 mm; HWL: 2.86 mm; HWW: 0.91 mm; t1: 0.40 mm; t2: 0.14 mm.

Color similar to male, slightly darker (Figure 2E–I). Forewing (Figure 2H) markings much larger than those in male. Antenna with 9–13 antennomeres whitish. Pterostigma with brown marking extending to mid of r-rs. Abdomen purplish red, 4–8 segments ventrally yellowish white. Genital segments dark brown.

Genital segments strongly sclerotized. Epiproct (Figure 3D) subtrapezoidal. Paraproct with 30 trichobothria. Subgenital plate (Figure 3E) with a broad sclerotized area. External valve short and robust, with a shark tip, almost perpendicular to dorsal valve (Figure 3F).

**Specimens examined. Holotype** of *Stenopsocus anthracinus*, ♂, China, Shaanxi, Zhenba (800 m), 1985.VII.20, Fasheng Li (CAU) (Figure S3); **Holotype** of *Stenopsocus angustifurcus*, ♂, China, Guizhou, Dushan (1000 m), 1981.VI.1, Fasheng Li (CAU) (Figure S4); **Holotype** of *Stenopsocus biconvexus*, ♂, China, Sichuan, Wushan, Liziping (1850 m), 1994.VI.1, Fasheng Li (CAU) (Figure S5); **Holotype** of *Stenopsocus bipunctatus*, ♀, China, Yunnan, Longling (900 m), 1989.IX.17, Fasheng Li (CAU) (Figure S6); **Holotype** of *Stenopsocus cassideus*, China, Hunan, Sangzhi, Mt. Tianping, 1981.IX.17, Xingwang Tong (CAU) (Figure S7); **Holotype** of *Stenopsocus dichospilus*, ♂, China, Anhui, Mt. Huangshan, Beihai (2000 m), 1977.VIII.23, Fasheng Li (CAU) (Figure S8); **Holotype** of *Stenopsocus flavifrons*, ♀, China, Shaanxi, Foping, Zhangliangmiao (1200 m), 1985.VII.23, Fasheng Li (CAU) (Figure S9); **Holotype** of *Stenopsocus fulivertex*, ♂, China, Hubei, Tongshan, Mt. Jiugong, 1984.VI.16, Chikun Yang (CAU) (Figure S10); **Holotype** of *Stenopsocus frontalis*, ♀, China, Shaanxi, Foping (1200 m), 1985.VII.16, Fasheng Li (CAU) (Figure S11); **Holotype** of *Stenopsocus parviforficatus*, ♂, China, Ningxia, Mt. Liupanshan, Erlong River (2100 m), 1980.VIII.4, Fasheng Li (CAU) (Figure S12); **Holotype** of *Stenopsocus podorphus*, ♀, China, Hubei, Xingshan, Longmen River (1300 m), 1994.IX.8, Fasheng Li (CAU) (Figure S13); **Holotype** of *Stenopsocus qianipullus*, ♀, China, Guizhou, Daozhen, Dashahe Nature Reserve (1400 m), 2004.VIII.17, Maofa Yang (CAU) (Figure S14); **Holotype** of *Stenopsocus symipsarous*, ♂, China, Guizhou, Huaxi (1000 m), Guiyang, Guizhou, 1981.V.22, Chikun Yang (CAU) (Figure S15).

CHINA: 3♀♀, Sichuan, Mt. Emei, Linggongli, 2012.IX.2, Xuankun Li (CAU); 1♀, Sichuan, Mt. Emei, Leidongping, 2012.IX.3, Ding Yang (CAU); 1♀, Sichuan, Mt. Emei, 2012.VI.8-15, Xiao Zhang (CAU); 1♀, Sichuan, Tianshidong (1000 m), Mt. Qingcheng, 1978.V.2, Fasheng Li (CAU); 1♀, Sichuan, Linggongli, Mt. Emei, 2010.VII.15, Junchao Wang (CAU); 3♂, Leidongping, Mt. Emei, Sichuan, 2012.IX.3, Xuankun Li (CAU); 1♀, 1♀, Xishan (2100 m), Kunming, Yunnan, 1981.V.16, Fasheng Li (CAU); 1♂, Yunnan, Nanjian, Mt. Wuliang (2221 m), 2016.VII.16, Qicheng Yang (CAU); 1♀, Chongqing, Yintiaoling, 2022.VII.8, Leran Cao (CAU); 2♂♂1♀, Hubei, Yinshan, Mt. Wujiashan (715 m), Wencheng Chang (CAU); 1♂1♀, Yunnan, Nujiang, Pianma, 2013.VII.6, Ziqiang Sun (CAU); 2♀♀, Yunnan, Pingbian, Mt. Daweishan (1962 m), 2016.VII.15, Yanan Lv (CAU).

**Distribution.** China (Shaanxi, Yunnan, Guizhou, Sichuan, Hubei, Hunan, Anhui, Guangxi, Zhejiang, Ningxia, Gansu).

**Remarks.** Li [2,39,40,41,42] described *N. anthracinus*, *N. flavifrons,* and *N. frontalis* from Shaanxi; *N. angustifurcus*, *N. qianpullus*, and *N. symipsarous* from Guizhou; *N. biconvexus* from Sichuan; *N. bipunctatus* from Yunnan; *N. cassideus* from Hunan; *N. dichospilus* from Anhui; *N. fulivertex* and *N. podorphus* from Hubei; and *N. parviforficatus* from Ningxia. Some of the above species were proposed based on a single male or female specimen. After examining the type specimens of each species and comparing them with the specimens collected in recent years, the latter 13 species are considered synonyms of *N. anthracinus* based on the yellowish vertex, the similar color of antenna, and the marking pattern of forewing. We matched the male and female of this species based on the DNA barcoding in the present study. The males and females of this species exhibit distinct differences in their pterostigma markings. Females possess a brown band along the posterior margin of the pterostigma, while males have an extremely narrow and inconspicuous brown band. *N. anthracinus* shares a similar external morphology with *N. tibialis*. In the NJ tree, these two species clustered within one clade. We consider these two species as valid based on the interspecific K2P distance of 0.1631. Moreover, *N. anthracinus* is distributed in the southern Chinese mainland, while *N. tibialis* is only distributed in Taiwan Island.

**Figure 4:**
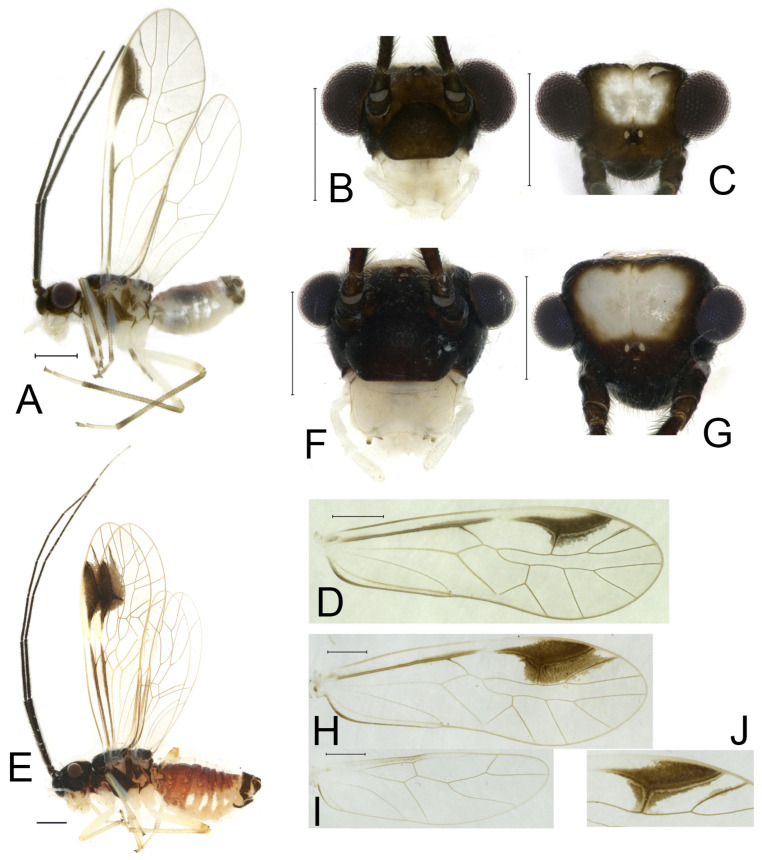
Photos of *Neostenopsocus capacimacularus*: (**A**) male, habitus; (**B**) male, frontal view of head; (**C**) male, dorsal view of head; (**D**) male, forewing; (**E**) female, habitus; (**F**) female, frontal view of head; (**G**) female, dorsal view of head; (**H**) female, forewing; (**I**) female, hind wing; (**J**) female, another marking pattern of pterostigma. Scale bar = 0.5 mm.

**Figure 5:**
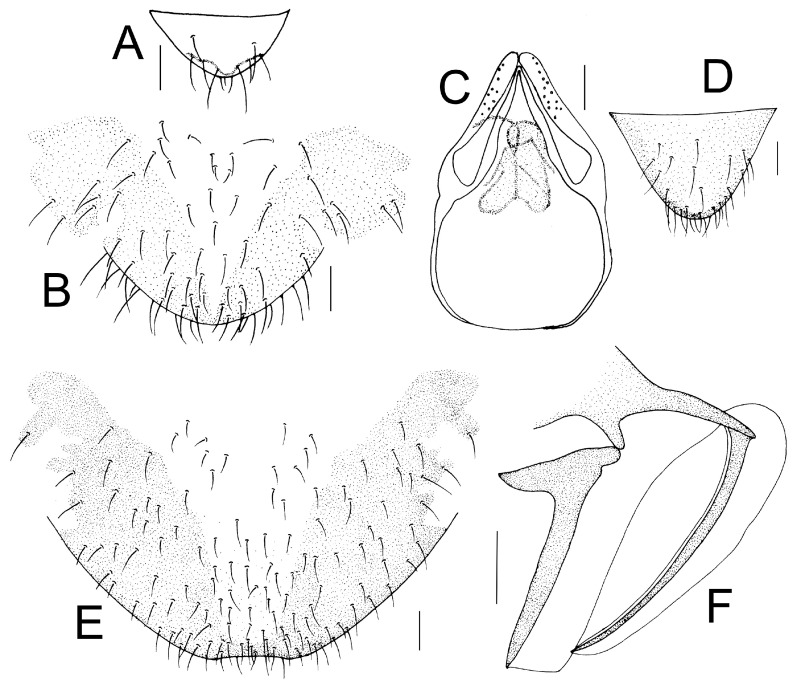
Drawing of *Neostenopsocus capacimacularus*: (**A**) male, epiproct; (**B**) male, hypandrium; (**C**) male, endophallus; (**D**) female, epiproct; (**E**) female, subgenital plate; (**F**) female, gonapophyses. Scale bar = 0.05 mm.

**(2) *Neostenopsocus capacimacularus* (Li, 1993) (**Figure 4 and Figure 5**)**

*Stenopsocus capacimacularus* Li, 1993 [43]: 347. Type locality: China (Guangdong: Chebaling).

*Stenopsocus tamdaoi* Georgiev & Quang-Manh, 2024 [44]: 4. Type locality: Vietnam (Vinh Phuc: Tam Dao). **syn. nov.**

**Diagnosis**. This species is characterized by the unique forewing markings near the pterostigma area.

**Redescription.** Adult male: Body (Figure 4A) length 2.55 mm, length from postclypeus to wing tip 4.00 mm. IO: 0.31 mm; d: 0.27 mm; IO/d = 1.15; f1: 0.94 mm; f2: 0.83 mm; f3: 0.64 mm; FWL: 3.38 mm; FWW: 1.11 mm; HWL: 2.55 mm; HWW: 0.75 mm; t1: 0.37 mm; t2: 0.12 mm.

Color (in alcohol): Head (Figure 4B) dark brown, vertex (Figure 4C) with a subtrapezoidal yellowish area. Antenna blackish brown. Mouthparts mostly yellowish with blackish-brown postclypeus, apex of maxillary palpus pale brown, remaining segments of maxillary palpus whitish. Thorax dark brown. Leg femur mostly yellowish, tibia brown, 1st tarsomeres and entire 2nd tarsomere brown. Abdomen dorsally pale purplish, ventrally yellowish white, genital segments brown.

Forewing transparent (Figure 4D). R dark brown. Pterostigma yellowish, with brown marking which do not extend along r-rs. Hindwing with pale brown markings present between Sc and R.

Genital segments strongly sclerotized. Epiproct (Figure 5A) subtriangular. Paraproct with 27 trichobothria. Hypandrium (Figure 5B) sclerotized strongly. Endophallus (Figure 5C) strongly sclerotized, external parameres robust, with some punctures on broad end toward apex, and exceeding apex of aedeagal arch; aedeagal arch narrow.

Adult female: Body (Figure 4E) length 4.10 mm, length from postclypeus to wing tip 5.33 mm. IO: 0.60 mm; d: 0.25 mm; IO/d = 2.4; f1: 1.29 mm; f2: 1.12 mm; f3: 0.84 mm; FWL: 4.17 mm; FWW: 1.41 mm; HWL: 3.07 mm; HWW: 0.92 mm; t1: 0.38 mm; t2: 0.14 mm.

Color similar to male, slightly darker. Forewing (Figure 4H) markings much larger than those in male. More than 2/3 area of pterostigma with a big brown marking. Abdomen purplish red, with 4–8 segments ventrally yellowish white.

Genital segments strongly sclerotized. Epiproct (Figure 5D) subtrapezoidal. Paraproct with 30 trichobothria. Subgenital plate (Figure 5E) with a broad sclerotized area. External valve short and robust, with a shark tip, almost perpendicular to dorsal valve (Figure 5F).

**Specimens examined. Holotype** of *Stenopsocus capacimacularus*, ♀, Chebaling Nature Reserve, Shixing, Guangdong, 1991.IV.23, Fasheng Li (CAU) (Figure S16).

CHINA: 5♀♀, Zhejiang, Lin’an, Mt. Tianmushan, 2012.IX.4, Hong Wu (Malaise trap) (CAU); 2♂♂2♀♀, Mt. Daweishan, Liuyang, Hunan, 2019.V.28, Feiyang Liang (HNUST); ♀, Guangxi, Nanning, Wuming, Mt. Damingshan, 2013.IX.23, Xingyue Liu (CAU); 2♀♀, Fujian, Nanping, Tongmu, 2009.VII.6, Xiushuai Yang (CAU); 9♀♀, Zhejiang, Lin’an, Mt. Tianmushan (300–1000 m), 2016.IV.27, Feiyang Liang (CAU); 1♂3♀♀, Guangxi, Jinxiu, Yinshan Park (1150 m), 2016.V.22, Feiyang Liang (CAU); 4♂♂, Guangxi, Jinxiu, Lianhuashan Park (950 m), 2016.V.22, Xingyue Liu (CAU); 1♀, Guangxi, Tianlin, Mt. Cenwanglaoshan, Dalongping Station, 2016.V.8, Xingyue Liu (CAU); 1♀, Zhejiang, Qingliangfeng, Tianchi, 2012.X, Malaise trap (CAU).

**Distribution.** China (Guangxi, Guangdong, Hunan, Zhejiang) and Vietnam (Vinh Phuc).

**Remarks.** Li [43] described *S. capacimacularus* based on a single female specimen from Chebaling Nature Reserve, Guangdong. Although no specimens were collected from the type locality during our study, recent examinations have identified several individuals from Guangxi, Hunan, and Zhejiang as conspecific based on the vertex coloration and forewing marking patterns. This species exhibits notable intraspecific variation in the forewing markings. The female specimens from Mt. Tianmu (Zhejiang) revealed two distinct marking patterns: a large-marked form (Figure 4H) and a small-marked form (Figure 4J). Additionally, male individuals sampled from Guangxi and Hunan were found to display forewing markings similar to the small-marked female form, though the size of these markings was relatively diminished. *S. tamdaoi* described from Tam Dao, northern Vietnam, near China, is herein synonymized with *N. capacimacularus* based on shared diagnostic characters: a uniformly yellowish vertex and an extensive brown forewing marking of the latter species.

**Figure 6:**
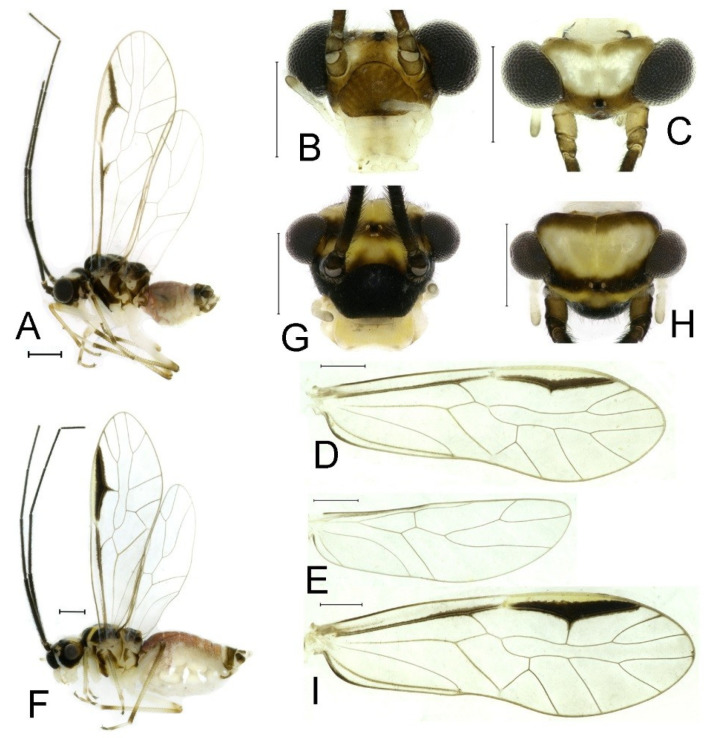
Photos of *Neostenopsocus dictyodromus*: (**A**) male, habitus; (**B**) male, frontal view of head; (**C**) male, dorsal view of head; (**D**) male, forewing; (**E**) male, hind wing; (**F**) female, habitus; (**G**) female, frontal view of head; (**H**) female, dorsal view of head; (**I**) female, forewing. Scale bar = 0.5 mm.

**Figure 7:**
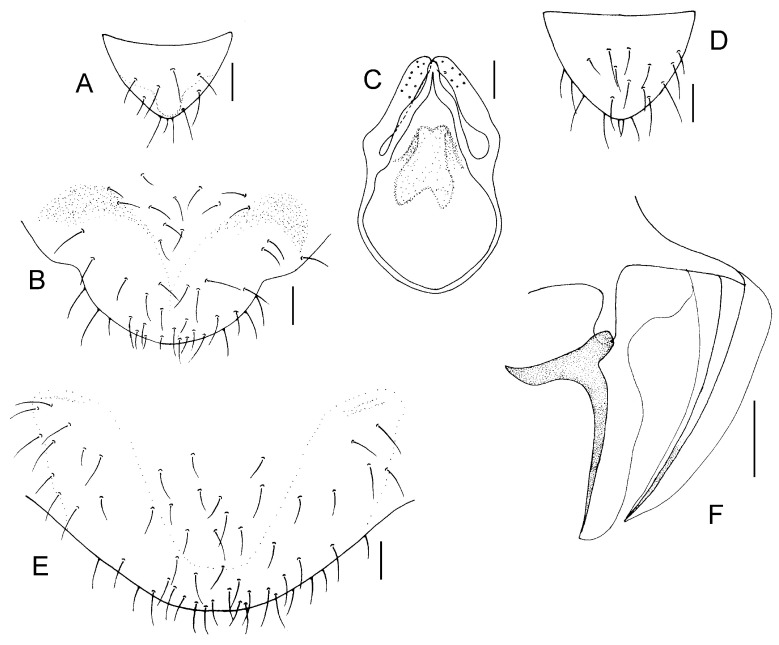
Drawing of *Neostenopsocus dictyodromus*: (**A**) male, epiproct; (**B**) male, hypandrium; (**C**) male, endophallus; (**D**) female, epiproct; (**E**) female, subgenital plate; (**F**) female, gonapophyses. Scale bar = 0.05 mm.

**Figure 8:**
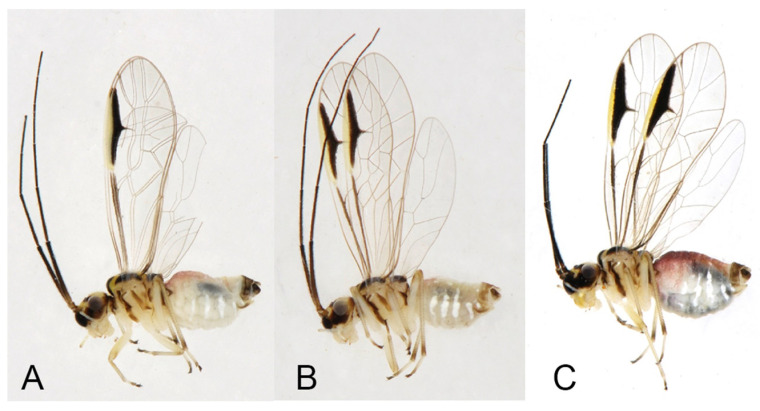
Habitus of *Neostenopsocus dictyodromus* from Yinshan Park, Guangxi, females. The markings of pterostigma display continuous variation. (**A**) a individual with a smaller pterostigmal dark brown marking; (**B**) a individual with a slightly larger dark brown marking on the pterostigma; (**C**) a individuals with the pterostigmal area nearly covered by a dark brown marking.

**(3) *Neostenopsocus dictyodromus* (Li, 1993) (**Figure 6, Figure 7 and Figure 8**)**

*Stenopsocus dictyodromus* Li, 1993 [43]: 349. Type locality: China (Guangdong: Chebaling).

*Stenopsocus beroni* Georgiev & Quang-Manh, 2024 [44]: 2. Type locality: Vietnam (Vinh Phuc: Tam Dao). **syn. nov.**

*Stenopsocus longitudinalis* Li, 2002 [2]: 623. Type locality: China (Guangxi: Tianlin). **syn. nov.**

*Stenopsocus trisetus* Li, 2002 [2]: 638. Type locality: China (Guangxi: Pingxiang). **syn. nov.**

**Diagnosis**. This species is characterized by a yellowish vertex, a yellowish frontal area with brown markings, posterior margin of pterostigma with brown stripe, and sclerotized genitalia.

**Redescription.** Adult male: Body (Figure 6A) length 2.51 mm, length from postclypeus to wing tip 3.89 mm. IO: 0.38 mm; d: 0.34 mm; IO/d = 1.12; f1: 0.99 mm; f2: 0.72 mm; f3: 0.65 mm; FWL: 3.96 mm; FWW: 1.32 mm; HWL: 3.01 mm; HWW: 0.91 mm; t1: 0.47 mm; t2: 0.15 mm.

Color (in alcohol): Head (Figure 6B) yellowish brown, vertex (Figure 6C) yellowish white, mid of frontal area with a brown marking. Antenna dark brown. Mouthparts yellowish white, with brown postclypeus. Thorax dark brown. Leg yellowish white, hindleg with brown tibia. Abdomen purplish red, with 4–7 segments ventrally yellowish white, genital segments brown.

Forewing (Figure 6D) transparent. Posterior margin of pterostigma with narrowly brown marking. Hindwing (Figure 6E) immaculate.

Genital segments strongly sclerotized. Epiproct (Figure 7A) subtriangular. Paraproct with 44 trichobothria. Hypandrium (Figure 7B) with strongly sclerotized area. Endophallus (Figure 7C) more sclerotized than hypandrium, external parameres robust, with some punctures on broad end toward apex, and exceeding apex of aedeagal arch.

Adult female: Body (Figure 6F) length 3.71 mm, length from postclypeus to wing tip 5.85 mm. IO: 0.58 mm; d: 0.25 mm; IO/d = 2.32; f1: 1.20 mm; f2: 1.05 mm; f3: 0.83 mm; FWL: 4.55 mm; FWW: 1.48 mm; HWL: 3.31 mm; HWW: 1.01 mm; t1: 0.47 mm; t2: 0.14 mm.

Color (in alcohol): Frontal area vertex (Figure 6G) yellowish white, with a brown stripe crossing ocellus area and connecting compound eyes. Vertex (Figure 6H) yellowish white. Antenna dark brown. Mouthparts yellowish, with dark brown postclypeus. Thorax brown. Leg yellowish white, hindleg with brown tibia. Abdomen dorsally purplish red, ventrally yellowish white, genital segments brown.

Forewing vertex (Figure 6I) yellowish white, transparent. Anterior area of pterostigma yellowish, posterior area of pterostigma with dark brown markings. Hindwing with a brown stripe between Sc and R.

Genital segments sclerotized. Epiproct vertex (Figure 7D) yellowish white, subtriangular. Paraproct with 25 trichobothria. Subgenital plate vertex (Figure 7E) yellowish white, with distinctly sclerotized area. Gonapophyses vertex (Figure 7F) yellowish white, strongly sclerotized.

**Specimens examined. Holotype** of *Stenopsocus dictyodromus*, ♀, China, Guangdong, Shixing, Chebaling National Nature Reserve (600 m), 1991.IV.23, Fasheng Li (CAU) (Figure S17); **Holotype** of *Stenopsocus longitudinalis*, ♀, China, Guangxi, Tianlin, Langping (1500 m), 1982.V.30, Fasheng Li (CAU) (Figure S18); **Holotype** of *Stenopsocus trisetus*, ♀, China, Guangxi, Pingxiang, Daqingshan, 1963.V.15, Chikun Yang (CAU) (Figure S19).

CHINA: 2♂♂2♀♀, Guangxi, Wuming, Mt. Damingshan (1230 m), 2013.V.20, Xingyue Liu (CAU); 1♀, Chongqing, Yintiaoling, 2022.VII.12, Leran Cao (CAU); 2♂♂8♀♀, Guangxi, Jinxiu, Yinshan Park (1150 m), 2016.V.22, Feiyang Liang (CAU); 2♀♀, Yunnan, Bingchuan, Mt. Jizushan (2228 m), 2016.VII.4, Liang Wang & Qicheng Yang (CAU); 1♀, Yunnan, Baoshan, Nankang (2048 m), 2015.VII.19, Yunlan Jiang (CAU); 1♀, Yunnan, Jinping, Jinhe Hotel (1792 m), 2016.VII.11, Ya’nan, Lv (CAU); ♂2♀♀, Yunnan, Malipo, Xiajinchang (1430 m), 2016.VII.27, Yunlan Jiang (CAU); 1♀, Fujian, Dehua, Leifengcun (850 m), 2010.VII.13, Xinyu Luo (CAU); 1♀, Chongqing, Yintiaoling Nature Reserve, Linkouzi Station (1248 m), 16.VIII.2022, Leran Cao (CAU).

**Distribution.** China (Guangdong, Guangxi, Yunnan, Chongqing, Fujian).

**Remarks.** The forewing markings display slight variations among different female individuals of this species. The brown marking within the forewing pterostigma exhibits continuous size variation, ranging from occupying less than half of the pterostigmal area to covering more than half of it (Figure 8). We consider *S. beroni*, *N. longitudinalis*, and *N. trisetus* to be synonymous with *N. dictyodromus* based on the similarities in the forewing marking patterns and sclerotized terminalia. This species appears to be related to *N. gracillimus* due to their similar head and forewing marking patterns; however, it can be distinguished from the latter by the sclerotized terminalia.

**Figure 9:**
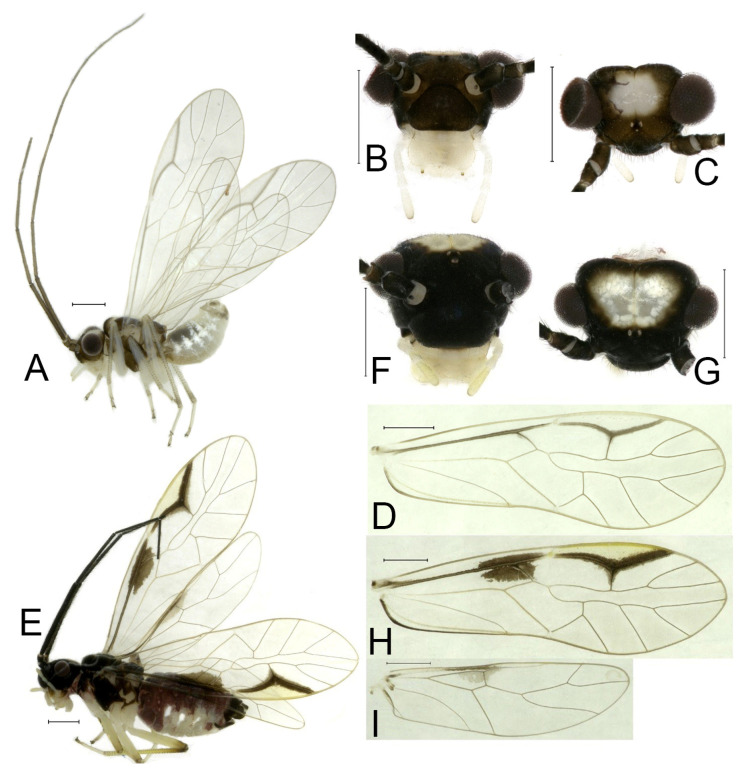
Photos of *Neostenopsocus eucallus*: (**A**) male, habitus; (**B**) male, frontal view of head; (**C**) male, dorsal view of head; (**D**) male, forewing; (**E**) female, habitus; (**F**) female, frontal view of head; (**G**) female, dorsal view of head; (**H**) female, forewing; (**I**) female, hind wing. Scale bar = 0.5 mm.

**Figure 10:**
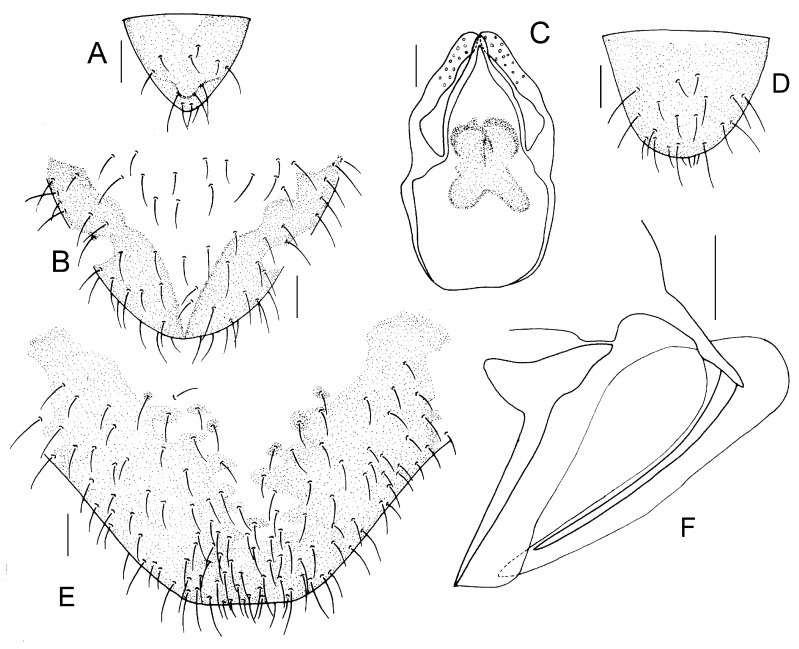
Drawing of *Neostenopsocus eucallus*; (**A**) male, epiproct; (**B**) male, hypandrium; (**C**) male, endophallus; (**D**) female, epiproct; (**E**) female, subgenital plate; (**F**) female, gonapophyses. Scale bar = 0.05 mm.

**(4) *Neostenopsocus eucallus* (Li & Yang, 1988) (Figure 9 and Figure 10** **)**

*Stenopsocus eucallus* Li & Yang, 1988 [45]: 174. Type locality: China (Guizhou: Fanjingshan).

*Stenopsocus metastictus* Li, 2002 [2]: 629. Type locality: China (Guangxi: Jinxiu). **syn. nov.**

**Diagnosis**. This species is characterized by a dark brown head with yellowish-white vertex, forewing R with a dark brown spot, posterior margin of pterostigma with brown stripe, hindwing with a brown spot between anterior margin and M, and strongly sclerotized genitalia.

**Redescription.** Adult male: Body (Figure 9A) length 2.46 mm, length from postclypeus to wing tip 4.82 mm. IO: 0.38 mm; d: 0.29 mm; IO/d = 1.31; f1: 1.01 mm; f2: 0.87 mm; f3: 0.71 mm; FWL: 3.49 mm; FWW: 1.18 mm; HWL: 2.58 mm; HWW: 0.81 mm; t1: 0.44 mm; t2: 0.12 mm.

Color (in alcohol): Head (Figure 9B,C) dark brown, vertex with a subtrapezoidal yellowish area. Antenna blackish brown. Mouthparts mostly yellowish with blackish-brown postclypeus, apex of maxillary palpus pale brown. Thorax dark brown. Leg yellowish. Abdomen yellowish white, genital segments brown.

Forewing (Figure 9D) transparent. R dark brown. Pterostigma yellowish, posterior margin with narrow brown marking which does not extend along r-rs. Hindwing with dark brown markings present between Sc and R.

Genital segments strongly sclerotized. Epiproct (Figure 10A) subtriangular. Paraproct with 32 trichobothria. Subgenital plate (Figure 10B) sclerotized strongly. Endophallus (Figure 10A) strongly sclerotized, external parameres robust, with some punctures on broad end toward apex, and not exceeding apex of aedeagal arch; aedeagal arch narrow.

Adult female: Body (Figure 9E) length 3.20 mm, length from postclypeus to wing tip 5.48 mm. IO: 0.67 mm; d: 0.25 mm; IO/d = 2.28; f1: 1.18 mm; f2: 1.10 mm; f3: 0.85 mm; FWL: 4.29 mm; FWW: 1.41 mm; HWL: 3.24 mm; HWW: 0.95 mm; t1: 0.41 mm; t2: 0.13 mm.

Color similar to male, slightly darker. Forewing (Figure 9H) markings much larger than those in male, R with a brown marking. Hindwing (Figure 9I) with dark brown markings present between Sc and R. Abdomen with 1–3 segments purplish red, 4–6 segments laterally purplish.

Genital segments strongly sclerotized. Epiproct (Figure 10D) subtrapezoidal. Paraproct with 20 trichobothria. Subgenital plate (Figure 10E) with a broad sclerotized area. External valve short and robust, with a flat tip, almost perpendicular to dorsal valve (Figure 10F).

**Specimens examined. Holotype** of *Stenopsocus eucallus*, ♀, Guizhou, Jiangkou, Mt. Fanjingshan (850 m), 1986.VIII.14, Fasheng Li (CAU) (Figure S20); **Holotype** of *Stenopsocus metastictus*, ♀, Guangxi, Jinxiu, Mt. Dayaoshan (800 m), 1982.VI.12, Fasheng Li (CAU) (Figure S21).

**CHINA:** 2♀♀, Guangxi, Jinxiu, Yinshan Park (1150 m), 2016.VII.22, Xingyue Liu (CAU); 2♂♂1♀, Yunnan, Pingbian, Daweishan Park, 2016.VII.16, Ya’nan Lv (CAU); 1♂, Yunnan, Baoshan, Xiaoheishan (2116 m), 2015.VII.20, Yunlan Jiang (CAU); 1♂, Yunnan, Baoshan, Mt. Gaoligongshan (2148 m), 2015.VII.20, Yunlan Jiang (CAU); 1♀, Sichuan, Mt. Emei, Linggongli, 2015.VIII.25, Tingting Zhang (CAU); 1♀, Xizang, Motuo (1117 m), 2014.VII.25, Yan Li (CAU).

**Distribution.** China (Yunnan, Guangxi, Guizhou, Sichuan, Xizang).

**Remarks.** Combined with the molecular delimitation results, this species shows stable coloration in the body and wings. However, there are differences in the markings of the forewings between male and female individuals. The R vein of male has no obvious markings, while that of female has a brown spot; the posterior marginal spot of the pterostigma in male is significantly narrower. We consider *S. metastictus* to be synonymous with *N. eucallus* based on the similarities in the forewing marking patterns and yellowish vertex. This species appears to be closely related to *N. formosanus* by the forewing marking patterns, but can be distinguished from the latter species by the hindwing with a larger brown spot.

**(5) *Neostenopsocus externus* (Banks, 1937)**

*Stenopsocus externus* Banks, 1937 [38]: 259. Type locality: China (Taiwan: Taihoku).

*Stenopsocus hemiostictus* Li, 2002 [2]: 659. Type locality: China (Yunnan: Kunming). **syn. nov.**

*Stenopsocus phaneostriatus* Li, 2002 [2]: 663. Type locality: China (Yunnan: Kunming). **syn. nov.**

**Diagnosis.** See Liang et al., 2015 [20].

**Specimens examined. Holotype** of *Stenopsocus externus*, ♀, China, Taiwan, Taipei, 1934.V.2, Judson Linsley Gressitt (MCZ) (Figure S22); **Paratype** of *Stenopsocus externus*, ♀, China, Taiwan, Arisan, 1934.V.29, Judson Linsley Gressitt (MCZ) (Figure S22); **Holotype** of *Stenopsocus hemiostictus*, ♀, China, Yunnan, Kunming, Xishan (2100 m), 1981.V.16, Fasheng Li (CAU) (Figure S23); **Holotype** of *Stenopsocus phaneostriatus*, ♀, China, Yunnan, Kunming, Xishan (1900 m), 1981.V.18, Fasheng Li (CAU) (Figure S24).

CHINA: 1♀, Taiwan, Kaohsiung, Shoushan Park, 2013.V.31, Xinyu Luo; 1♀, Taiwan, Yilan, Wushibi, 2013.VI.8, Xinyu Luo (CAU); 1♀, Zhejiang, Li’an, Mt. Tianmu (300–1000 m), 2016.VII.27, Feiyang Liang (CAU); 1♂2♀♀, Guangxi, Wuming, Daminshan, 2016.V.25, Feiyang Liang (CAU); 2♀♀, Fujian, Xiamen, Mt. Dongpingshan, 2021.VII.2, Yuchen Zheng (CAU); 1♂, Guizhou, Mt. Leigongshan, Xiaodanjiang (664 m), 2014.VII.23, Lu Yue (CAU); 1♀, Guizhou, Mt. Leigongshan, Xiaodanjiang (664 m), 2014.VII.23, Yuting Dai (CAU); 1♀, Guangxi, Pingxiang, Lanhuagu, 2014.V.8, Xiumei Lu (CAU); 1♂, Guangxi, Nanning, Guangxi Academy of Forestry Sciences, 2013.V.14, Xingyue Liu (CAU); 1♀, Guangxi, Fangchenggang, Wangle Nature Reserve, 2013.V.18, Xingyue Liu (CAU); 2♂♂, Guangxi, Fangchenggang, Shiwandashan Forest Park, 2013.V.17, Guoquan Wang (CAU); 1♂, Guangxi, Fangchenggang, Jinhuacha Nature Reserve, 2014.VI.7, Xingyue Liu (CAU); 1♂, Yunnan, Menglun, Xishuangbanna Tropical Botanical Garde, 2015.IV.10-13, Feiyang Liang (CAU); 1♀, Guangxi, Baise, Songshuping, 2013.VIII.1, Feiyang Liang (CAU); 1♂4♀♀, Henan, Xinxian, Jiulongtan (200 m), 2014.VI.18, Xingyue Liu (CAU); 1♀, Xizang, Linzhi, Lulang, 2012.VII.18, Chenliang Zhang & Jianyun Wang (CAU); 1♂1♀, Hubei, Yinshan, Mt. Wujiashan (715 m), 2014.VI.30, Wencheng Chang (CAU); 1♂1♀, Hunan, Xiangtan, Hunan University of Science and Technology, 2019.V.30, Feiyang Liang (HNUST). LAOS: 1♂, Khammouan, Phou Hi Poun NBCA, near Tha Long (550 m), 2016.IV.1, Xingyue Liu (CAU). VIETNAM: ♀, Kon Tum, Chu Mom Ray National Park, 2012.VIII.1, Feiyang Liang (CAU).

**Distribution.** China (Taiwan, Guangxi, Hunan, Hubei, Henan, Fujian, Yunnan, Guizhou, Sichuan, Gansu, Hebei, Xizang, Shanghai); Laos (Khammouan); Vietnam (Kon Tum).

**Remarks.** Li [2] and Liang et al. [20] reported this species is widely distributed in southern China. In the present study, we matched the male and female of this species based on the DNA barcoding. This species is diagnosable by the brown pedicel and the distal half of R1 with a brown marking. Li [2] described *S. hemiostictus* and *S. phaneostriatus* from Kunming and Yunnan, noting that their antennae have yellowish 1st antennomere, brown 2nd–10th antennomeres, and half of the posterior margin of the pterostigma on the forewings bears brown markings. After examining the relevant specimens, we found that these characteristics are consistent with the diagnostic features of *S. externus*, and consider *S. hemiostictus* and *S. phaneostriatus* to be synonyms of *N. externus*.

**Figure 11:**
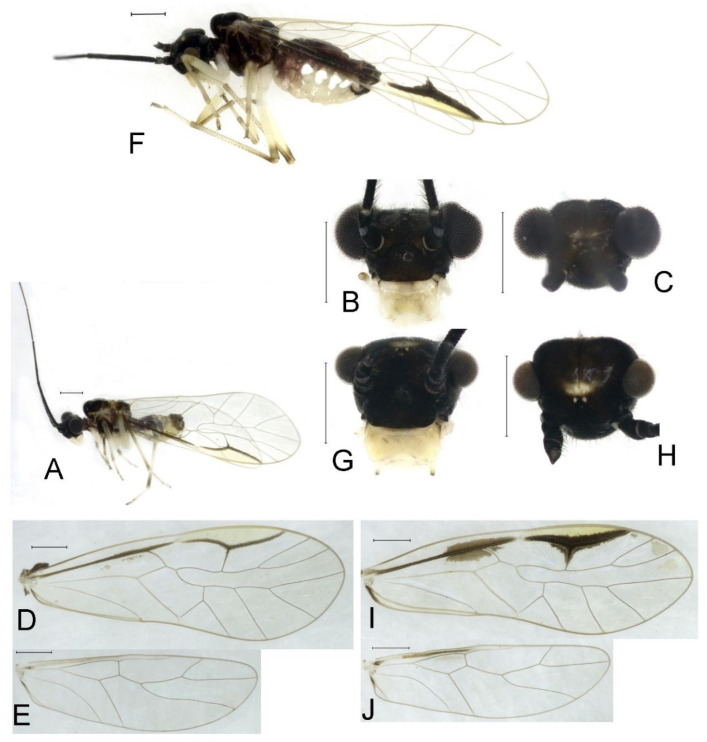
Photos of *Neostenopsocus foliaceus*: (**A**) male, habitus; (**B**) male, frontal view of head; (**C**) male, dorsal view of head; (**D**) male, forewing; (**E**) male, hind wing; (**F**) female, habitus; (**G**) female, frontal view of head; (**H**) female, dorsal view of head; (**I**) female, forewing; (**J**) female, hind wing. Scale bar = 0.5 mm.

**Figure 12:**
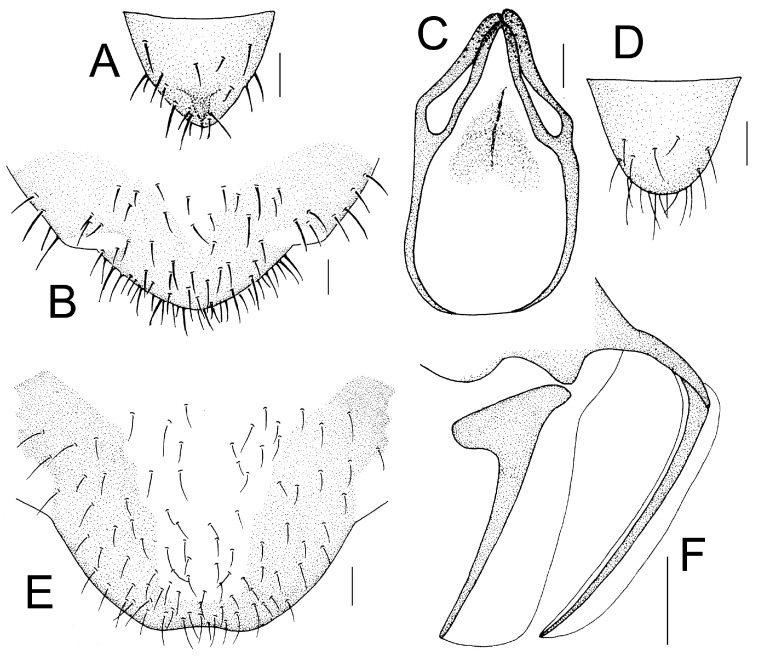
Drawing of *Neostenopsocus foliaceus*: (**A**) male, epiproct; (**B**) male, hypandrium; (**C**) male, endophallus; (**D**) female, epiproct; (**E**) female, subgenital plate; (**F**) female, gonapophyses. Scale bar = 0.05 mm.

**(6) *Neostenopsocus foliaceus*** (**Li, 1997) (**Figure 11 and Figure 12**)**

*Stenopsocus foliaceus* Li, 1997 [40]: 434. Type locality: China (Hubei: Xingshan).

**Diagnosis**. This species is characterized by a dark brown head, forewing R with brown spot, pterostigma with a subtriangle brown spot, and a strongly sclerotized terminalia.

**Redescription.** Adult male: Body (Figure 11A) length 2.13 mm, length from postclypeus to wing tip 3.92 mm. IO: 0.39 mm; d: 0.30 mm; IO/d = 1.30; f1: 0.82 mm; f2: 0.63 mm; f3: 0.51 mm; FWL: 2.91 mm; FWW: 1.06 mm; HWL: 2.31 mm; HWW: 0.72 mm; t1: 0.32 mm; t2: 0.11 mm.

Color (in alcohol): Head (Figure 11C) dark brown. Antenna blackish brown. Mouthparts mostly yellowish with blackish-brown postclypeus, apex of maxillary palpus pale brown. Thorax dark brown. Leg brown to dark brown, with femur mostly yellowish white. Abdomen yellowish white, genital segments dark brown.

Forewing (Figure 11D) transparent. R dark brown. Pterostigma yellowish, posterior margin with narrow brown marking which does not extend along r-rs. Hindwing (Figure 11E) with pale brown markings present between Sc and R.

Genital segments strongly sclerotized. Epiproct (Figure 12A) subtriangular. Paraproct with 29 trichobothria. Hypandrium (Figure 12B) sclerotized strongly. Endophallus (Figure 12C) strongly sclerotized, external parameres robust, with some punctures on broad end toward apex, and not exceeding apex of aedeagal arch; aedeagal arch narrow.

Adult female: Body (Figure 11F) length 2.90 mm, length from postclypeus to wing tip 4.64 mm. IO: 0.52 mm; d: 0.19 mm; IO/d = 2.74; f1: 0.91 mm; f2: 0.67 mm; f3: 0.52 mm; FWL: 3.62 mm; FWW: 1.22 mm; HWL: 2.63 mm; HWW: 0.82 mm; t1: 0.34 mm; t2: 0.13 mm.

Color similar to male, slightly darker. Forewing (Figure 11I) markings much larger than those in male, R with a brown marking. Abdomen dorsally purplish red, ventrally yellowish white, genital segments dark brown.

Genital segments strongly sclerotized. Epiproct (Figure 12D) subtrapezoidal. Paraproct with 33 trichobothria. Subgenital plate (Figure 12E) with a broad sclerotized area. External valve short and robust, with a round apex (Figure 12F).

**Specimens examined. Holotype** of *Stenopsocus foliaceus*, ♀, China, Hubei, Xingshan, Longmenhe (1300 m), 1994.IX.13, Fasheng Li (CAU) (Figure S25).

CHINA: 1♂, Sichuan, Mt. Emei, Leidongping (2300 m), 2012.IX.2, Ding Yang (CAU); 1♀, Sichuan, Mt. Emei, Leidongping (2300 m), 2012.IX.3, Xuankun Li (CAU); 4♀♀, Shaanxi, Lingwu, 2013.VII.13, Wanzhi Cai & Jianyun Wang (CAU).

**Distribution.** China (Hubei, Sichuan, Shaanxi).

**Remarks.** This species differs from *N. eucallus* by the dark brown vertex, and differs from *N. melanocephalus* by the forewing R with a large brown spot.

**Figure 13:**
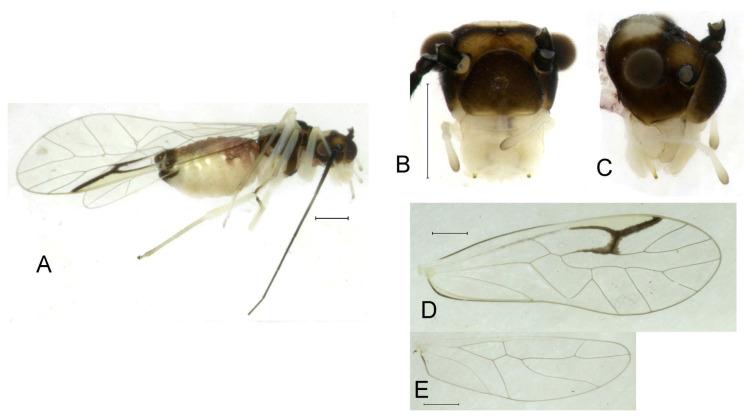
Photos of *Neostenopsocus hexagonus*: (**A**) male, habitus; (**B**) male, frontal view of head; (**C**) male, dorsal view of head; (**D**) male, forewing; (**E**) male, hind wing. Scale bar = 0.5 mm.

**Figure 14:**
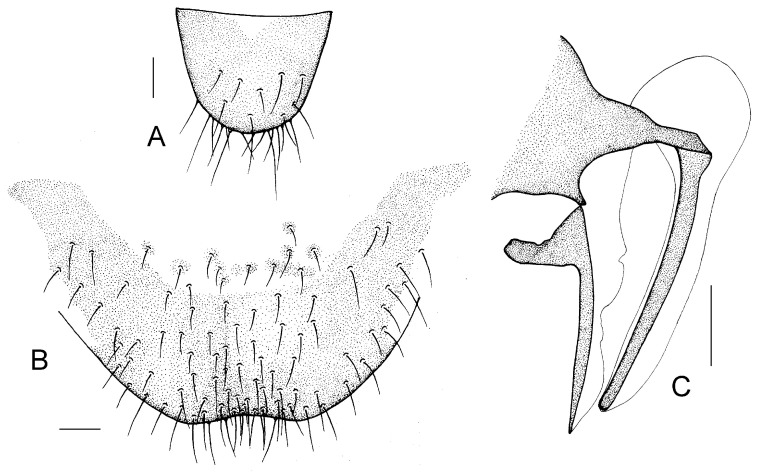
Drawing of *Neostenopsocus hexagonus*: (**A**) female, epiproct; (**B**) female, subgenital plate; (**C**) female, gonapophyses. Scale bar = 0.05 mm.

**(7) *Neostenopsocus hexagonus* (Li, 2002) (**Figure 13 and Figure 14**)**

*Stenopsocus hexagonus* Li, 2002 [2]: 633. Type locality: China (Xizang: Bomi).

**Diagnosis**. This species is characterized by a yellowish vertex, dark brown antenna, yellowish-white leg, and forewing with a Y-shape marking near the pterostigma.

**Redescription.** Adult male: Unknown.

Adult female: Body (Figure 13A) length 2.78 mm, length from postclypeus to wing tip 5.05 mm. IO: 0.51 mm; d: 0.19 mm; IO/d = 2.73; f1: 1.03 mm; f2: 0.71 mm; f3: 0.69 mm; FWL: 4.37 mm; FWW: 1.44 mm; HWL: 3.12 mm; HWW: 0.93 mm; t1: 0.46 mm; t2: 0.13 mm.

Color (in alcohol): Head (Figure 13B) brown, vertex (Figure 13C) yellowish white. Antenna blackish brown. Mouthparts mostly yellowish with blackish-brown postclypeus, apex of maxillary palpus pale brown, remaining segments of maxillary palpus whitish. Thorax dark brown. Leg yellowish white. Abdomen dorsally pale purplish red, ventrally yellowish white, genital segments brown.

Forewing (Figure 13D) transparent. Pterostigma yellowish, posterior margin with narrow brown stripe extending along r-rs. Hindwing (Figure 13E) immaculate.

Genital segments strongly sclerotized. Epiproct (Figure 14A) subtrapezoidal. Paraproct with 24 trichobothria. Subgenital plate (Figure 14B) with a broad sclerotized area. External valve short and robust, almost perpendicular to dorsal valve (Figure 14C).

**Specimens examined. Holotype** of *Stenopsocus hexagonus*, ♀, China, Xizang, Bomi, Zhamu (2800 m), 1978.VII.22, Fasheng Li (CAU) (Figure S26).

CHINA: 3♀♀, Xizang, Linzhi, 2012.IX.22-X.1, Malaise trap (CAU).

**Distribution.** China (Xizang).

**Remarks.** This species can be distinguished from *N. polyceratus* by a forewing R without markings, and the narrower makings near the pterostigma.

**Figure 15:**
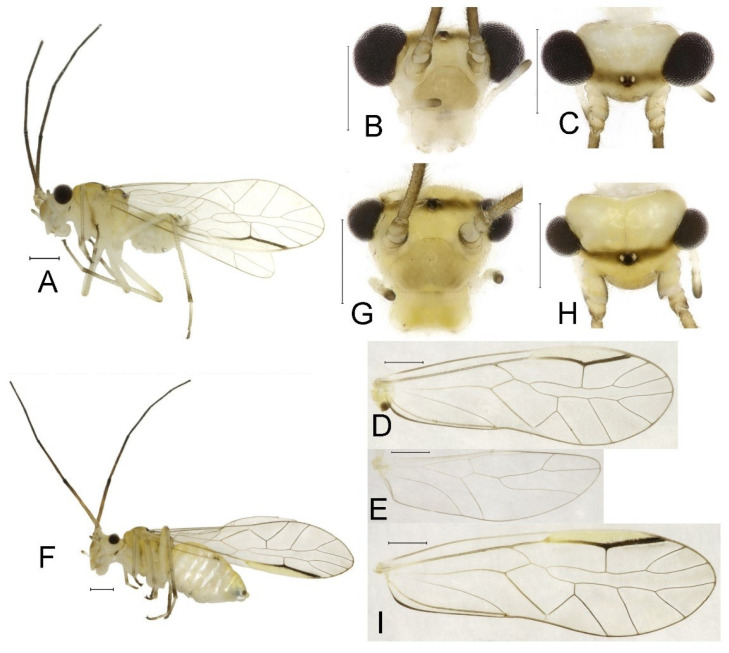
Photos of *Neostenopsocus kunmingiensis*: (**A**) male, habitus; (**B**) male, frontal view of head; (**C**) male, dorsal view of head; (**D**) male, forewing; (**E**) male, hind wing; (**F**) female, habitus; (**G**) female, frontal view of head; (**H**) female, dorsal view of head; (**I**) female, forewing. Scale bar = 0.5 mm.

**Figure 16:**
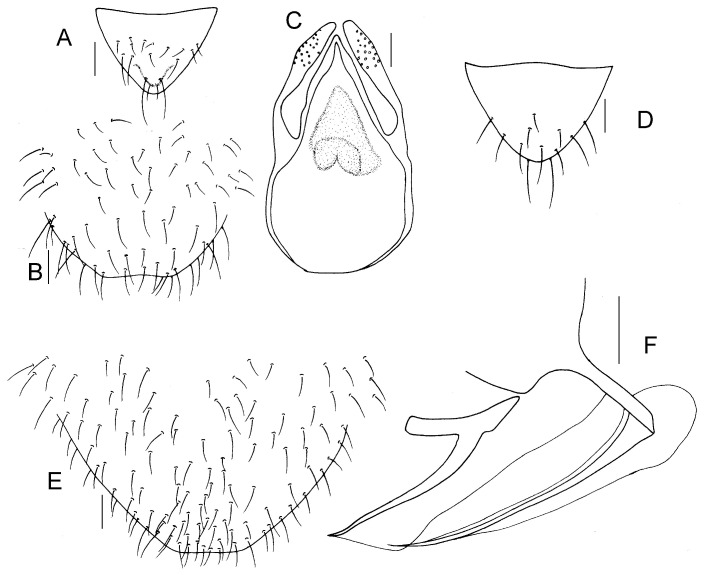
Drawing of *Neostenopsocus kunmingiensis*: (**A**) male, epiproct; (**B**) male, hypandrium; (**C**) male, endophallus; (**D**) female, epiproct; (**E**) female, subgenital plate; (**F**) female, gonapophyses. Scale bar = 0.05 mm.

**(8) *Neostenopsocus kunmingiensis* (Li, 2002) (**Figure 15 and Figure 16**)**

*Stenopsocus kunmingiensis* Li, 2002 [2]: 681. Type locality: China (Yunnan: Kunming).

*Stenopsocus pavonicus* Li, 2002 [2]: 697. Type locality: China (Yunnan: Mengzhe). **syn. nov.**

**Diagnosis**. This species is characterized by a yellowish head with a brown stripe crossing the ocellus area and connecting compound eyes, 1st–5th flagellomeres yellowish-brown with brown tips, posterior margin of pterostigma with very narrow brown stripe, and weakly sclerotized terminalia.

**Redescription.** Adult male: Body (Figure 15A) length 2.44 mm, length from postclypeus to wing tip 4.86 mm. IO: 0.42 mm; d: 0.33 mm; IO/d = 1.27; f1: 0.97 mm; f2: 0.86 mm; f3: 0.70 mm; FWL: 3.86 mm; FWW: 1.31 mm; HWL: 2.94 mm; HWW: 0.94 mm; t1: 0.47 mm; t2: 0.14 mm.

Color (in alcohol): Head (Figure 15B,C) yellowish white. Frontal area with a brown stripe crossing ocellus area and connecting compound eyes. Antenna with yellowish-brown scape and pedicel, and blackish-brown flagella. Mouthparts yellowish, apex of maxillary palpus brown, remaining segments of maxillary palpus whitish. Prothorax yellowish, mid- and metathorax laterally yellowish and dorsally brown. Leg yellowish, with apex of tibiae, 1st tarsomeres, and entire 2nd tarsomere pale brown. Abdomen yellowish white, only trichobothrial area brown.

Forewing (Figure 15D) transparent. Pterostigma pale yellowish, posterior margin with narrow brown marking. Hindwing (Figure 15E) immaculate.

Genital segments weakly sclerotized. Epiproct (Figure 16A) subtriangular. Paraproct with 31 trichobothria. Hypandrium (Figure 16B) without distinctly sclerotized area. Endophallus (Figure 16C) weakly sclerotized; external parameres robust, with some punctures on broad end toward apex and not exceeding apex of aedeagal arch; aedeagal arch narrow.

Adult female: Body (Figure 15F) length 3.66 mm, length from postclypeus to wing tip 5.79 mm. IO: 0.57 mm; d: 0.23 mm; IO/d = 2.48; f1: 1.23 mm; f2: 1.03 mm; f3: 0.77 mm; FWL: 4.43 mm; FWW: 1.41 mm; HWL: 3.15 mm; HWW: 0.95 mm; t1: 0.36 mm; t2: 0.11 mm.

Color similar to male, slightly darker. Forewing (Figure 15I) markings much larger than those in male, R with a brown marking. Abdomen with 1–3 segments purplish red, 4–6 segments laterally purplish.

Genital segments strongly sclerotized. Epiproct (Figure 16D) subtrapezoidal. Paraproct with 20 trichobothria. Subgenital plate (Figure 16E) with a broad sclerotized area. External valve short and robust, with a flat tip, almost perpendicular to dorsal valve (Figure 16F).

**Specimens examined. Holotype** of *Stenopsocus kunmingiensis*, ♂, China, Yunnan, Kunming (1900 m), 1981.V.18, Fasheng Li (CAU).

CHINA: 3♂♂2♀♀, Xiajinchang (1430 m), Malipo, Yunnan, 2016.VII.27, Yunlan Jiang (CAU).

**Distribution.** China (Yunnan).

**Remarks.** This species is one with a relatively light body color in the genus *Neostenopsocus*; most of its body is pale yellow with few brown markings. In particular, the head only bears a brown band between the compound eyes, while all other areas are yellow.

**Figure 17:**
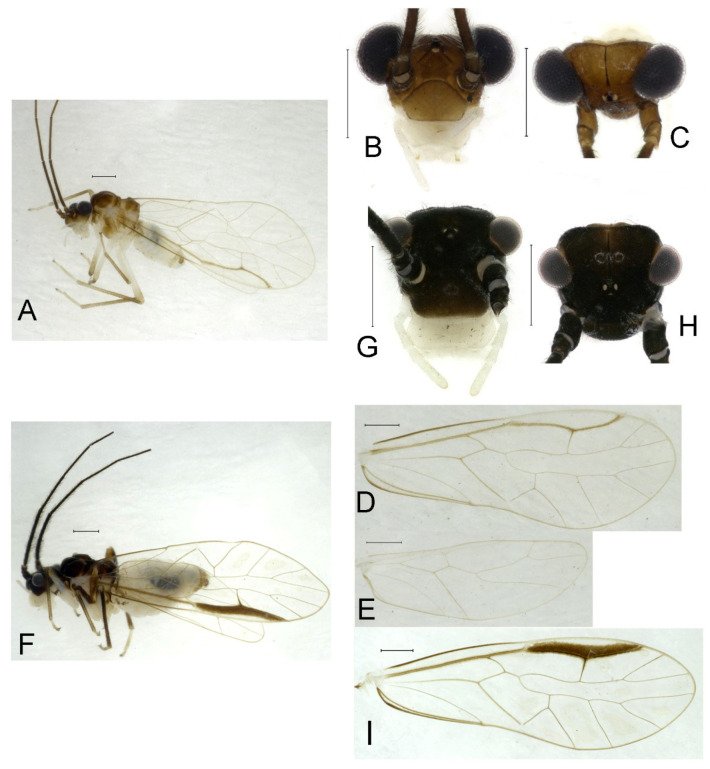
Photos of *Neostenopsocus maculosus*: (**A**) male, habitus; (**B**) male, frontal view of head; (**C**) male, dorsal view of head; (**D**) male, forewing; (**E**) male, hind wing; (**F**) female, habitus; (**G**) female, frontal view of head; (**H**) female, dorsal view of head; (**I**) female, forewing. Scale bar = 0.5 mm.

**Figure 18:**
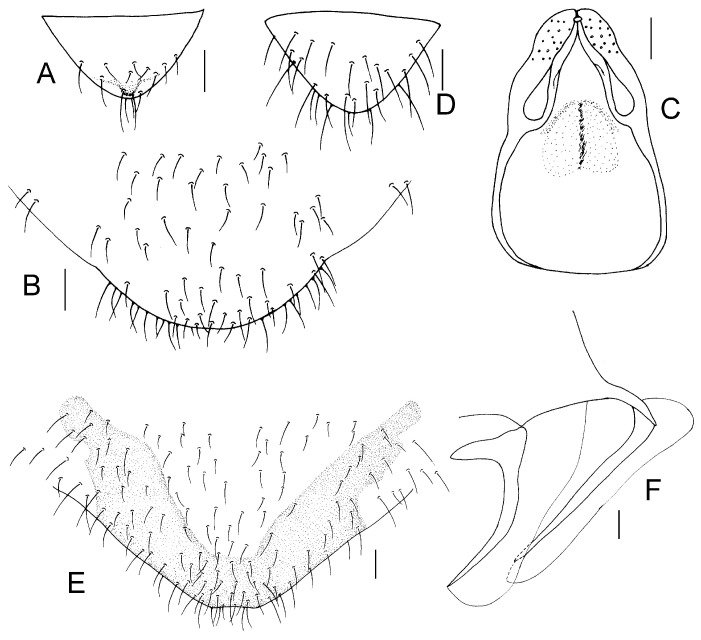
Drawing of *Neostenopsocus maculosus*: (**A**) male, epiproct; (**B**) male, hypandrium; (**C**) male, endophallus; (**D**) female, epiproct; (**E**) female, subgenital plate; (**F**) female, gonapophyses. Scale bar = 0.05 mm.

**(9) *Neostenopsocus maculosus* (Li & Yang, 1988) (**Figure 17 and Figure 18**)**

*Stenopsocus maculosus* Li & Yang, 1988 [45]: 73. Type locality: China (Guizhou: Fanjingshan).

*Stenopsocus brevicapitus* Li, 1997 [40]: 429. Type locality: China (Sichuan: Wushan). **syn. nov.**

*Stenopsocus dactylinus* Li, 1997 [40]: 431. Type locality: China (Sichuan: Wushan). **syn. nov.**

*Stenopsocus daozheniensis* Li, 2005 [42]: 92. Type locality: China (Guizhou: Dashahe). **syn. nov.**

*Stenopsocus isotomus* Li, 2002 [2]: 673. Type locality: China (Sichuan: Emeishan). **syn. nov.**

*Stenopsocus perspicuus* Li, 1997 [40]: 427. Type locality: China (Sichuan: Wushan). **syn. nov.**

*Stenopsocus shennongjiaensis* Li, 2002 [2]: 624. Type locality: China (Hubei: Shennongjia). **syn. nov.**

*Stenopsocus wuxiaensis* Li, 1997 [40]:432. Type locality: China (Sichuan: Wushan). **syn. nov.**

*Stenopsocus xilingxianicus* Li, 1997 [40]: 430. Type locality: China (Hubei: Xingshan). **syn. nov.**

**Diagnosis**. This species is characterized by a large body (female), mostly black–brown head, and a head equal in length (from vertex to margin of labrum in frontal view) and width (distance of the outer margins of the two compound eyes).

**Redescription.** Adult male: Body (Figure 17A) length 2.76 mm, length from postclypeus to wing tip 5.46 mm. IO: 0.36 mm; d: 0.27 mm; IO/d = 1.33; f1: 1.07 mm; f2: 0.93 mm; f3: 0.76 mm; FWL: 4.60 mm; FWW: 1.75 mm; HWL: 3.36 mm; HWW: 1.13 mm; t1: 0.50 mm; t2: 0.18 mm.

Color (in alcohol): Head (Figure 17B,C) dark brown. Antenna blackish brown. Mouthparts mostly yellowish with blackish-brown postclypeus. Thorax dark brown. Leg yellowish, with dark brown tibia. Abdomen yellowish white, genital segments pale brown with brown trichobothrial area.

Forewing (Figure 17D) transparent. R dark brown. Pterostigma without distinct markings. Hindwing (Figure 17E) immaculate.

Genital segments weakly sclerotized. Epiproct (Figure 18A) subtriangular. Paraproct with 29 trichobothria. Hypandrium (Figure 18B) without distinct sclerotized area. Endophallus (Figure 18C) weakly sclerotized; external parameres robust, with some punctures on broad end toward apex and not exceeding apex of aedeagal arch; aedeagal arch narrow.

Adult female: Body (Figure 17F) length 3.70 mm, length from postclypeus to wing tip 6.90 mm. IO: 0.36 mm; d: 0.16 mm; IO/d = 2.25; f1: 1.14 mm; f2: 0.81 mm; f3: 0.76 mm; FWL: 5.12 mm; FWW: 1.76 mm; HWL: 3.45 mm; HWW: 1.06 mm; t1: 0.52 mm; t2: 0.18 mm.

Color similar to male, slightly darker. Forewing (Figure 17I) with a brown stripe along posterior margin of Pterostigma.

Genital segments sclerotized. Epiproct (Figure 18D) subtrapezoidal. Paraproct with 36 trichobothria. Subgenital plate (Figure 18E) with a V-shape sclerotized area. External valve (Figure 18F) short and robust, with a round tip.

**Specimens examined. Holotype** of *Stenopsocus maculosus*, ♀, China, Guizhou, Jiangkou, Fanjingshan Nature Reserve, 1983.VII.13, Fasheng Li (CAU) (Figure S27); **Holotype** of *Stenopsocus brevicapitus*, ♀, China, Sichuan, Wushan, Liziping (1850 m), 1994.IX.21, Fasheng Li (CAU) (Figure S28); **Holotype** of *Stenopsocus dactylinus*, ♀, China, Sichuan, Wushan, Liziping (1850 m), 1994.IX.21, Fasheng Li (CAU) (Figure S29); **Holotype** of *Stenopsocus daozheniensis*, ♀, Guizhou, Daozhen, Dashahe Nature Reserve (1400 m), 2004.V.25, Maofa Yang (CAU) (Figure S30); **Holotype** of *Stenopsocus isotomus*, ♂, China, Sichuan, Mt. Emei, Jiulaodong (2070 m), 1978.IX.18, Fasheng Li (CAU) (Figure S31); **Holotype** of *Stenopsocus perspicuus*, ♀, China, Sichuan, Wushan, Liziping (1850 m), 1994.IX.21, Fasheng Li (CAU) (Figure S32); **Holotype** of *Stenopsocus shennongjiaensis*, ♂, China, Hubei, Shennongjia Nature Reserve, Dayanwu, Hubei, 1984.VI.19, Chikun Yang (CAU) (Figure S33); **Holotype** of *Stenopsocus wuxiaensis*, ♀, China, Sichuan, Wushan, Liziping (1850 m), 1994.IX.21, Fasheng Li (CAU) (Figure S34); **Holotype** of *Stenopsocus xilingxianicus*, ♀, China, Hubei, Xingshan, Longmen River (1300 m), 1994.IX.13, Fasheng Li (CAU) (Figure S35).

CHINA: 1♂1♀, Sichuan, Mt. Emei, Leidongping to Jinding, 2013.VI.29, Xingyue Liu & Feiyang Liang (CAU); 1♂, Sichuan, Mt. Emei, Linggongli, 2011.VII.5, Xingyue Liu (CAU); 1♀, Sichuan, Mt. Emei, Leidongping, 2015.VIII.23, Tingting Zhang (CAU); 1♀, Hubei, Shennongjia Nature Reserve, Dalongtan, 2012.VII.29, Tingting Zhang (CAU); 1♀, Shaanxi, Lingwu, 2011.VII.25, Wanzhi Cai & Jianyun Wang (CAU); 1♀, Gansu, Wenxian, Qiujiaba Station, 2012.VII.29, Sipei Liu (CAU).

**Distribution.** China (Guizhou, Sichuan, Hubei, Shaanxi, Gansu).

**Remarks.** This species can be distinguished from other *Neostenopsocus* based on the whole black–brown vertex, and the length of its head nearly equal to its width in the frontal view. Li & Yang [45] described *N. maculosus* from Mt. Fanjingshan, Guizhou. Subsequently, Li [2,40] described six additional species from three adjacent collection localities: *S. brevicapitus*, *S. dactylinus*, *S. perspicuus*, and *S. wuxiaensis* from Liziping; *S. shennongjiaensis* from Shennongjia; and *S. xilingxianicus* from Xingshan. After examining the type specimens of these six species, we found that the differences among these specimens primarily lie in the depth of body color: the body color of *S. wuxiaensis* and *S. shennongjiaensis* is relatively dark, while the body color of the specimens of other species is lighter. Besides this, there are no other obvious differences. Therefore, we consider these species to be the same species. These lighter-colored specimens may be individuals that have just emerged and have not yet fully developed their coloration. These specimens can be distinguished from the type specimen of *N. maculosus* by the forewing markings, specifically the markings along the posterior margin of the pterostigma, which are significantly narrower than those in *N. maculosus*. However, we collected several specimens from Shennongjia that exhibit broad markings consistent with those of *N. maculosus*. Therefore, these six species are considered synonyms of *N. maculosus*, as are *N. daozheniensis* and *N. isotomus*. Notably, the shape of head in *N. maculasus* is similar to the genera *Malostenopsocus* and *Stenopsocus*, with the length of head nearly equal to its width, which was identified by Liang & Liu [5] as a synapomorphy for these two genera.

**Figure 19:**
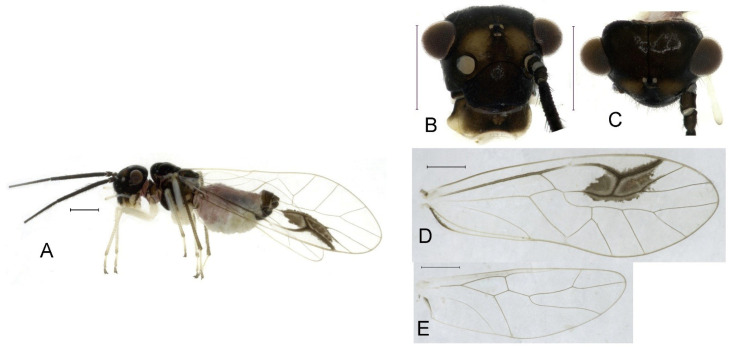
Photos of *Neostenopsocus maximalis*: (**A**) female, habitus; (**B**) female, frontal view of head; (**C**) female, dorsal view of head; (**D**) female, forewing; (**E**) female, hind wing. Scale bar = 0.5 mm.

**Figure 20:**
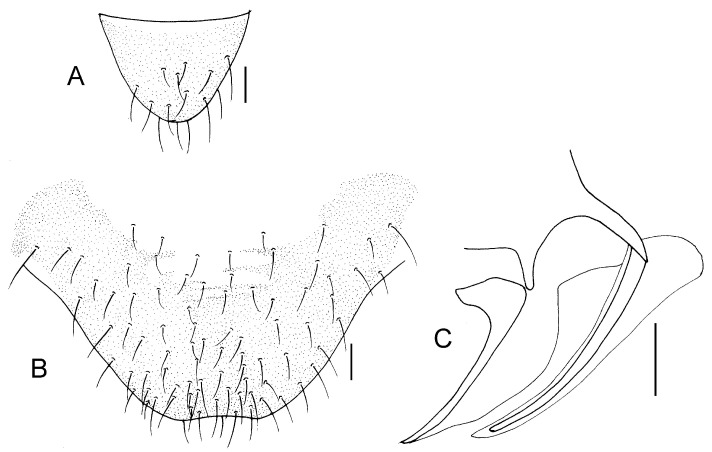
Drawing of *Neostenopsocus maximalis*: (**A**) female, epiproct; (**B**) female, subgenital plate; (**C**) female, gonapophyses. Scale bar = 0.05 mm.

**(10) *Neostenopsocus maximalis* (Li, 1997) (**Figure 19 and Figure 20**)**

*Stenopsocus maximalis* Li, 1997 [40]: 433. Type locality: China (Hubei: Xingshan).

**Diagnosis**. This species is characterized by a dark brown labrum and big markings behind pterostigma.

**Redescription.** Adult male: Unknown.

Adult female: Body (Figure 19A) length 2.98 mm, length from postclypeus to wing tip 4.91 mm. IO: 0.47 mm; d: 0.17 mm; IO/d = 2.61; f1: 1.12 mm; f2: 0.70 mm; f3: 0.64 mm; FWL: 3.87 mm; FWW: 1.36 mm; HWL: 2.74 mm; HWW: 0.94 mm; t1: 0.37 mm; t2: 0.10 mm.

Color (in alcohol): Head (Figure 19B,C) dark brown. Antenna with 1–6 antennomeres blackish brown, 7–13 antennomeres white. Postclypeus, labrum, maxilla stipes, and base of mandible darkish brown. Other part of mouthparts yellowish. Thorax dark brown. Leg yellowish white, hindleg with brown tibia. Abdomen dorsally pale purplish, ventrally yellowish white, genital segments dark brown.

Forewing (Figure 19D) transparent, wing margin mostly pale brown; costal vein from base to pterostigma and R brown, a big brown marking around crossvein r-rs. Hindwing (Figure 19E) transparent and glabrous.

Genitalia sclerotized. Epiproct (Figure 20A) subtrapezoid, with round apex. Paraproct with 22 trichobothrias. Subgenital plate (Figure 20B) with distinctly “U” shape sclerotized region, middle sclerotized region broad. Gonapophyses (Figure 20C) sclerotized; external valve broad, perpendicular to dorsal valve; dorsal and ventral valve blade shaped.

**Specimens examined. Holotype** of *Stenopsocus maximalis*, ♀, China, Hubei, Xingshan, Longmen River (1300 m), 1994.IX.13, Fasheng Li (CAU) (Figure S36).

CHINA: 4♀♀, Yunnan, Yuanjiang Nature Reserve, Yangchajie, 2015.IV.20, Feiyang Liang (CAU); 1♀, Xizang, Motuo, 2013.IX.12, Jianyuan Wang & Yun Ji (CAU); 2♀♀, Sichuan, Mt. Emei, 2015.VIII.25, Tingting Zhang (CAU); 1♀, Yunnan, Nujiang, Pianma, 2013.VII. 6, Ziqiang Sun (CAU); 3♀♀, Yunnan, Boshan, Mt. Baihualing, 1900 m, 2015.VII.26, Lu Yue (CAU); 1♀, Yunnan, Mt. Gaoligong, Xiaoheishan (2116 m), 2015.VII.20, Yunlan Jiang (CAU); 1♀, Yunnan, Nanjiang, Mt. Wuliang (2221 m), 2016.VII.16, Qicheng Yang (CAU). **Nepal:** 3♀♀, Pokhara, Mt. Annapurna (2000 m), 2014.VII. 2, Feiyang Liang (CAU).

**Distribution.** China (Hubei, Chongqing, Yunan, Xizang), Nepal (Pokhara).

**Remarks.** This species appears to be closely related to *N. capacimacularus* and *N. makii,* having similar body color and markings of forewing, but it can be distinguished from the latter two species by the black–brown vertex and labrum.

**Figure 21:**
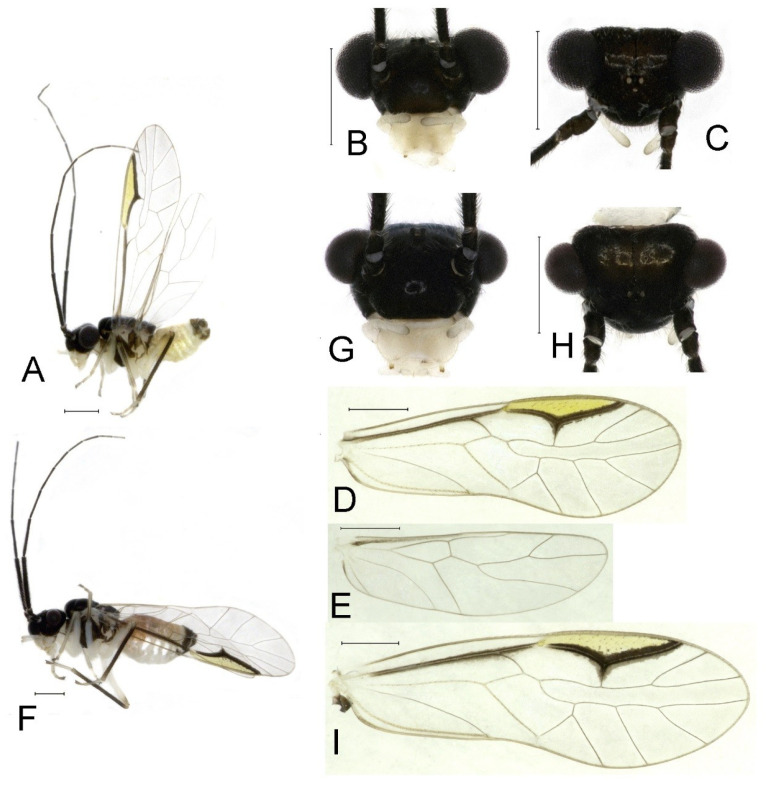
Photos of *Neostenopsocus melanocephalus*: (**A**) male, habitus; (**B**) male, frontal view of head; (**C**) male, dorsal view of head; (**D**) male, forewing; (**E**) male, hind wing; (**F**) female, habitus; (**G**) female, frontal view of head; (**H**) female, dorsal view of head; (**I**) female, forewing. Scale bar = 0.5 mm.

**Figure 22:**
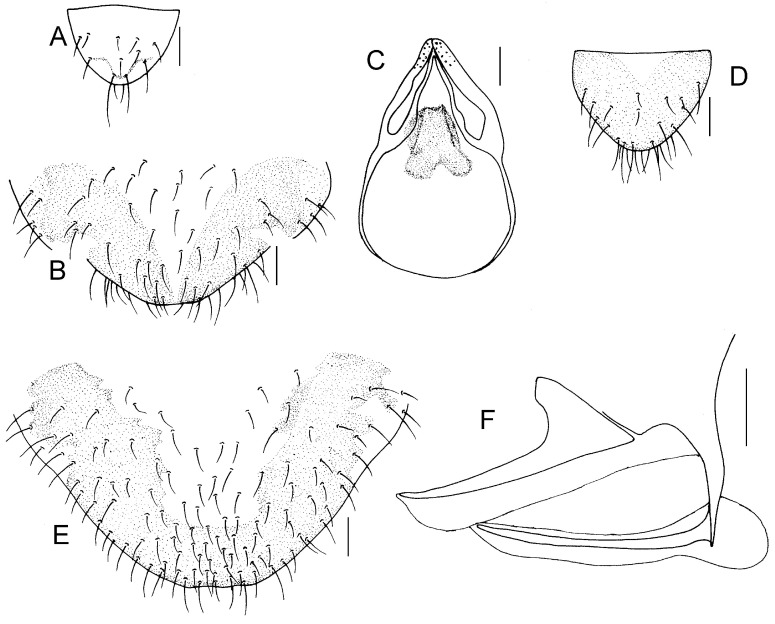
Drawing of *Neostenopsocus melanocephalus*: (**A**) male, epiproct; (**B**) male, hypandrium; (**C**) male, endophallus; (**D**) female, epiproct; (**E**) female, subgenital plate; (**F**) female, gonapophyses. Scale bar = 0.05 mm.

**(11) *Neostenopsocus melanocephalus* (Li, 1997) (**Figure 21 and Figure 22**)**

*Stenopsocus melanocephalus* Li, 1997 [40]: 437. Type locality: China (Sichuan: Wushan).

**Diagnosis**. This species is characterized by a whole brown or dark brown antenna, forewing R without marking, pterostigma with brown marking extending along r-rs, and hindleg with dark brown tibia.

**Redescription.** Adult male: Body (Figure 21A) length 2.79 mm, length from postclypeus to wing tip 5.41 mm. IO: 0.37 mm; d: 0.29 mm; IO/d = 1.28; f1: 1.13 mm; f2: 1.01 mm; f3: 0.79 mm; FWL: 4.63 mm; FWW: 1.66 mm; HWL: 3.47 mm; HWW: 1.13 mm; t1: 0.46 mm; t2: 0.16 mm.

Color (in alcohol): Head (Figure 21B,C) dark brown. Antenna blackish brown. Mouthparts mostly yellowish with blackish-brown postclypeus, apex of maxillary palpus pale brown, remaining segments of maxillary palpus whitish. Thorax dark brown. Leg yellowish, with dark brown tibiae. Abdomen dorsally purplish brown, ventrally yellowish white, genital segments brown.

Forewing (Figure 21D) transparent. R dark brown. Pterostigma yellowish, posterior margin with narrow brown marking, which does not extend along r-rs. Hindwing (Figure 21E) with pale brown markings present between Sc and R.

Genital segments strongly sclerotized. Epiproct (Figure 22A) subtriangular. Paraproct with 26 trichobothria. Hypandrium (Figure 22B) with V-shaped sclerotized area. Endophallus (Figure 22C) strongly sclerotized; external parameres robust, with some punctures on broad end toward apex and not exceeding apex of aedeagal arch; aedeagal arch narrow.

Adult female: Body (Figure 21F) length 3.92 mm, length from postclypeus to wing tip 6.33 mm. IO: 0.44 mm; d: 0.24 mm; IO/d = 1.82; f1: 1.38 mm; f2: 1.11 mm; f3: 0.84 mm; FWL: 5.37 mm; FWW: 1.13 mm; HWL: 3.60 mm; HWW: 1.13 mm; t1: 0.51 mm; t2: 0.18 mm.

Color similar to male, slightly darker. Forewing (Figure 21I) markings much larger than those in male, R with a brown marking. Abdomen purplish red, with 4–8 segments ventrally yellowish white.

Genital segments strongly sclerotized. Epiproct (Figure 22D) subtrapezoidal. Paraproct with 28 trichobothria. Subgenital plate (Figure 22E) with a broad sclerotized area. External valve short and robust, with a flat tip, almost perpendicular to dorsal valve (Figure 22F).

**Specimens examined. Holotype** of *Stenopsocus melanocephalus*, ♀, Sichuan, Mt. Wushan, Liziping (1850 m), 1994.IX.21, Fasheng Li (CAU) (Figure S37).

CHINA: 1♂, Sichuan, Mt. Emei, Leidongping, 2012.IX.2, Ding Yang (CAU); 1♀, Sichuan, Mt. Emei, Leidongping, 2012.IX.3, Xuankun Li (CAU); 4♀, Shaanxi, Lingwu, 2013.VII.13, Jianyun Wang & Wanzhi Cai (CAU); 1♀, Hunan, Sangzhi, Mt. Tianpingshan (1300 m), 2012.VII.23, Mingchao Huang (CAU); 4♂♂, Yunnan, Pingbian, Daweishan Nature Reserve, 2016.VII.17, Ya’nan Lv (CAU); 2♀♀, Yunnan, Pingbian, Daweishan Nature Reserve, 2016.VII.17, Ya’nan Lv (CAU); 2♂♂2♀♀, Guangxi, Jinxiu, Yinshan Park (1500 m), 2016.V.22, Feiyang Liang (CAU).

**Distribution.** China (Yunnan, Sichuan, Guangxi, Shaanxi).

**Remarks.** This species appears to be closely related to *N. anthracinus* and *N. tibialis,* having similar forewing marking patterns, but it can be distinguished from the latter two species by the whole blackish-brown vertex.

**Figure 23:**
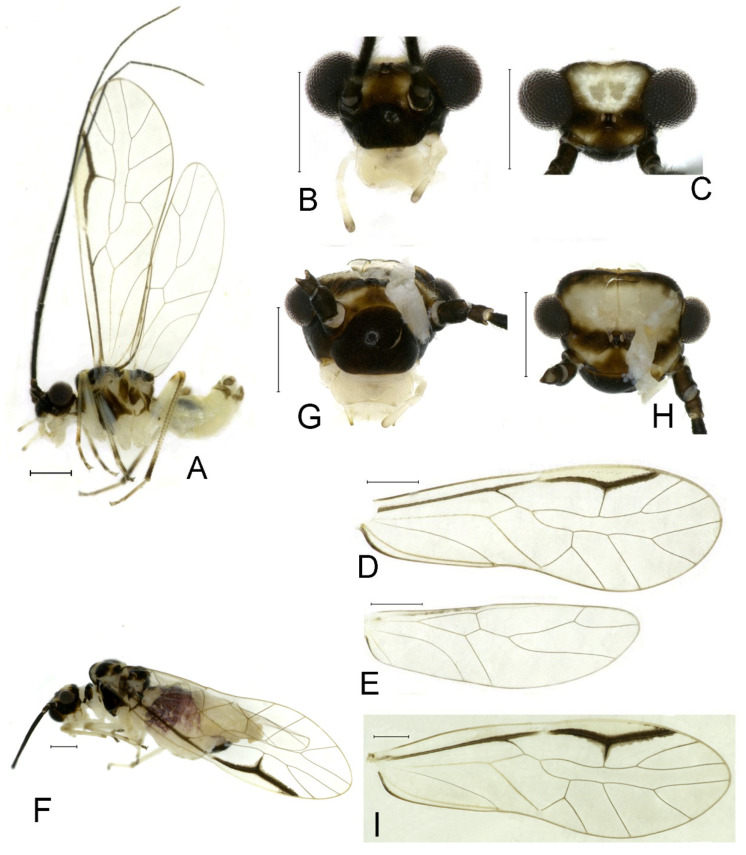
Photos of *Neostenopsocus nepalensis*: (**A**) male, habitus; (**B**) male, frontal view of head; (**C**) male, dorsal view of head; (**D**) male, forewing; (**E**) male, hind wing; (**F**) female, habitus; (**G**) female, frontal view of head; (**H**) female, dorsal view of head; (**I**) female, forewing. Scale bar = 0.5 mm.

**Figure 24:**
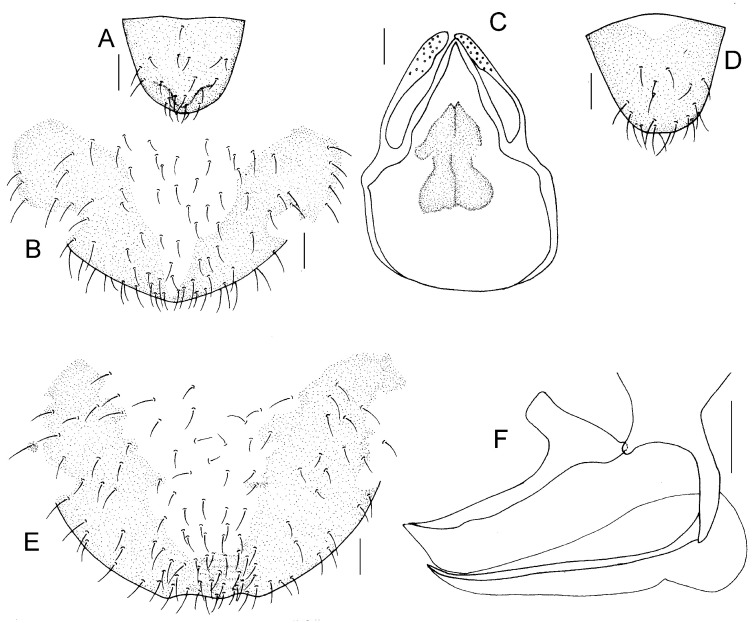
Drawing of *Neostenopsocus nepalensis*: (**A**) male, epiproct; (**B**) male, hypandrium; (**C**) male, endophallus; (**D**) female, epiproct; (**E**) female, subgenital plate; (**F**) female, gonapophyses. Scale bar = 0.05 mm.

**(12) *Neostenopsocus nepalensis* (New, 1971) (**Figure 23 and Figure 24**)**

*Stenopsocus nepalensis* New, 1971 [46]: 207. Type locality: Nepal (Bagmati).

**Diagnosis.** The females are characterized by a yellowish-white vertex, forewing R with a brown spot, posterior margin of pterostigma with dark brown stripe, hindwing with a narrow brown strip between Sc and R, and strongly sclerotized terminalia. The males are similar to the females, but lack the forewing marking on the R.

**Redescription.** Adult male: Body (Figure 23A) length 2.66 mm, length from postclypeus to wing tip 4.63 mm. IO: 0.34 mm; d: 0.30 mm; IO/d = 1.13; f1: 0.93 mm; f2: 0.86 mm; f3: 0.66 mm; FWL: 3.53 mm; FWW: 1.24 mm; HWL: 2.71 mm; HWW: 0.83 mm; t1: 0.42 mm; t2: 0.14 mm.

Color (in alcohol): Head (Figure 23B) dark brown, vertex (Figure 23C) with a subtrapezoidal yellowish area. Antenna blackish brown. Mouthparts mostly yellowish with blackish-brown postclypeus, apex of maxillary palpus pale brown, remaining segments of maxillary palpus whitish. Thorax brown. Leg yellowish, with yellow-brown tibia. Abdomen yellowish white, genital segments brown.

**Figure 25:**
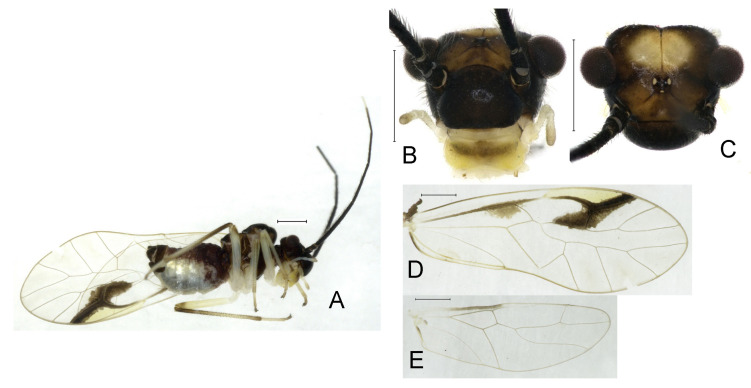
Photos of *Neostenopsocus polyceratus*: (**A**) female, habitus; (**B**) female, frontal view of head; (**C**) female, dorsal view of head; (**D**) female, forewing; (**E**) female, hind wing. Scale bar = 0.5 mm.

Forewing (Figure 23D) transparent. R dark brown. Pterostigma yellowish, posterior margin with narrow brown marking, which does not extend along r-rs. Hindwing (Figure 25E) with dark brown markings present between Sc and R.

Genital segments strongly sclerotized. Epiproct (Figure 24A) subtriangular, weakly sclerotized. Paraproct with 32 trichobothria. Hypandrium (Figure 24B) sclerotized strongly. Endophallus (Figure 24C) strongly sclerotized, external parameres robust, with some punctures on broad end toward apex and not exceeding apex of aedeagal arch; aedeagal arch narrow.

Adult female: Body (Figure 23F) length 3.46 mm, length from postclypeus to wing tip 5.27 mm. IO: 0.53 mm; d: 0.21 mm; IO/d = 2.52; f1: 1.30 mm; f2: 1.03 mm; f3: 0.75 mm; FWL: 4.29 mm; FWW: 1.41 mm; HWL: 3.24 mm; HWW: 0.95 mm; t1: 0.41 mm; t2: 0.13 mm.

Color similar to male, slightly darker. Forewing (Figure 23I) markings much larger than those in male, R with a brown marking. Abdomen with 1–3 segments purplish red, 4–6 segments laterally purplish.

Genital segments strongly sclerotized. Epiproct (Figure 24D) subtrapezoidal. Paraproct with 20 trichobothria. Subgenital plate (Figure 24E) with a broad sclerotized area. External valve short and robust, with a flat tip, almost perpendicular to dorsal valve (Figure 24F).

**Specimens examined.** CHINA: ♀, Xizang, Motuo, 2014.VII.12, Jianyun Wang & Yun Ji (CAU); 1♂1♀, Yunnan, Mt. Huanglianshan Nature Reserve, Yakou station, 2016.VII.9, Ya’nan Lv (CAU); 1♀, Guizhou, Mt. Fanjingshan, Tongkuangchang (850 m), 1988.VIII.14, Fasheng Li (CAU); 1♂, Guangxi, Jinxiu, Yinshan Park (1150 m), 2016.V.22, Feiyang Liang (CAU); 1♂3♀♀, Yunnan, Pingbian, Mt. Daweishan (1962 m), Ya’nan Lv (CAU); 1♀, Sichuan, Mianyang, Laohegou (1400–1800 m), 2016.V.14-15, Hui Dong (CAU); 1♀, Yunnan, Mt. Gaoligong Nature Reserve, Nankang Station (2048 m), 2015.VII.7, Lu Yue (CAU). NEPAL: 3♀♀, Pokhara, Mt. Annapura (2000 m), 2014.VII.17, Feiyang Liang (CAU).

**Distribution.** China (Yunnan, Guizhou, Sichuan, Guangxi, Xizang), Nepal (Bagmati, Pokhara).

**Remarks.** This species is newly recorded herein from China. It differs diagnostically from *N. eucallus* by the forewing R vein without markings in females.

**Figure 26:**
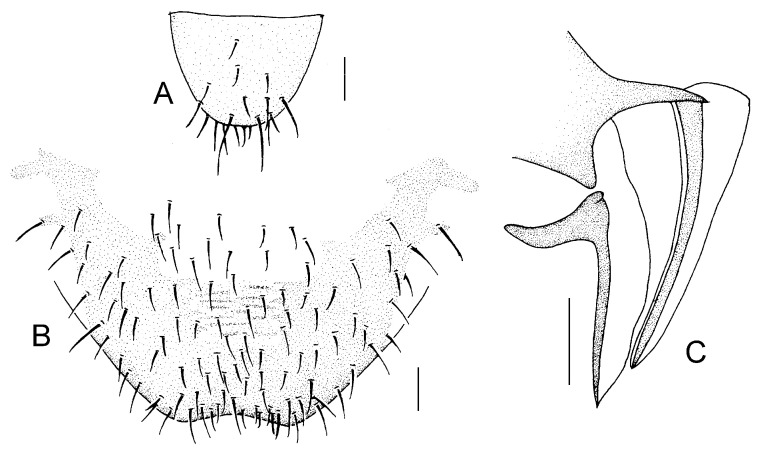
Drawing of *Neostenopsocus polyceratus*: (**A**) female, epiproct; (**B**) female, subgenital plate; (**C**) female, gonapophyses. Scale bar = 0.05 mm.

**(13) *Neostenopsocus polyceratus* (Li, 2002) (**Figure 25 and Figure 26**)**

*Stenopsocus polyceratus* Li, 2002 [2]: 613. Type locality: China (Ningxia: Liupanshan).

**Diagnosis.** This species differs from the other species of *Neostenopsocus* by the forewing with a y-shaped brown marking between the pterostigma and Rs.

**Redescription.** Adult male: Unknown.

Adult female. Body (Figure 25A) length 2.78 mm, length from postclypeus to wing tip 5.14 mm. IO: 0.49 mm; d: 0.20 mm; IO/d = 2.45; f1: 0.98 mm; f2: 0.73 mm; f3: 0.58 mm; FWL: 4.13 mm; FWW: 1.54 mm; HWL: 2.87 mm; HWW: 1.00 mm; t1: 0.44 mm; t2: 0.12 mm.

Color (in alcohol): Head (Figure 25B) dark brown, vertex (Figure 25C) with a yellowish oval area, frontal area yellowish brown, antenna with 1–9 antennomeres dark brown, 9–13 antennomeres whitish. Mouthparts mostly yellowish with blackish-brown postclypeus, apex of maxillary palpus pale brown, remaining segments of maxillary palpus whitish. Thorax brown or dark brown. Fore- and midleg mostly yellowish; hind leg mostly brown, with trochanter, middle of tibia, and entire 2nd tarsomere whitish. Abdomen purplish brown, with 4–8 segments ventrally whitish; genital segments dark brown.

Forewing (Figure 25D) transparent. Mid of R with a brown spot, anterior margin of pterostigma yellowish brown. Pterostigma yellowish, a y-shaped dark brown marking between posterior margin of pterostigma and Rs. Hindwing (Figure 25E) with a dark brown marking present between Sc and R.

Genital segments strongly sclerotized. Epiproct (Figure 26A) subtriangular. Paraproct with 22 trichobothria. Subgenital plate (Figure 26B) with a broad sclerotized area. External valve (Figure 26C) short, thumb shaped.

**Specimens examined. Holotype** of *Stenopsocus polyceratus*, ♀, Ningxia, Mt. Liupanshan (2100 m), 1992.VII.30, Fasheng Li (CAU) (Figure S38).

CHINA: 13♀♀, Shaanxi, Lingwu, 2013.VII.13, Wanzhi Cai & Jianyun Wang (CAU); 3♀♀, Ningxia, Zhongwei, Shapotou (1200 m), 1992.VIII.1, Fasheng Li (CAU); 4♀♀, Ningxia, Mt. Liupanshan, Liangdian Gorge, 2012.VIII.2, Yang Zhao (CAU); 2♀♀, Ningxia, Liupanshan Botanical Garden, 2012.VII.28, Yang Zhao (CAU); 4♀♀, Ningxia, Mt. Liupanshan, Xiaonanchuan, 2012.VII.27, Yang Zhao (CAU); 1♀, Sichuan, Mt Emei, Leidongping, 2011.VII.5, Xingyue Liu (CAU); 1♀, Sichuan, Mt Emei, Leidongping, 2011.VII.14, Yingying Wang (CAU).

**Distribution.** China (Gansu, Shaanxi, Ningxia, Sichuan).

**Remarks.** Li [2] described *S. polyceratus* from Gansu and *S. brachyodicrus* from Ningxia. Based on examination of additional specimens from Shaanxi, Ningxia, and Sichuan, we synonymize *S. brachyodicrus* under *S. polyceratus* based on the similar color of head, antenna, and markings on forewing.

**Figure 27:**
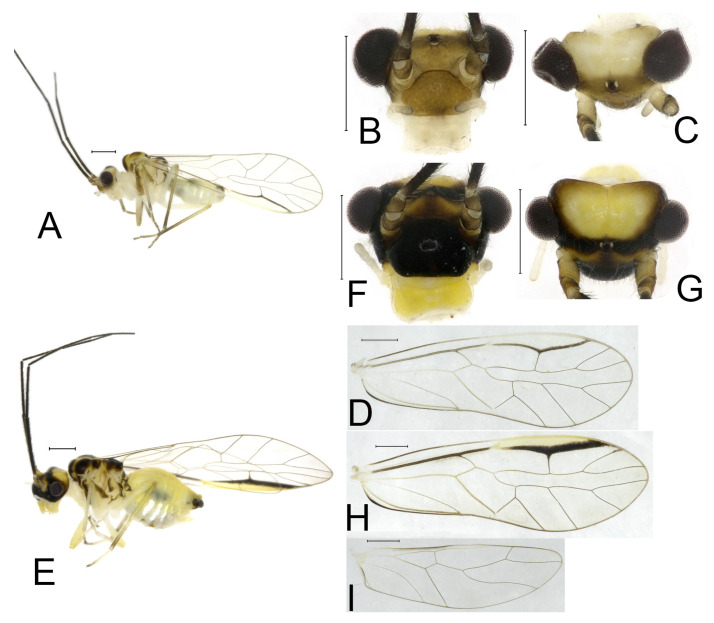
Photos of *Neostenopsocus zonatus*: (**A**) male, habitus; (**B**) male, frontal view of head; (**C**) male, dorsal view of head; (**D**) male, forewing; (**E**) female, habitus; (**F**) female, frontal view of head; (**G**) female, dorsal view of head; (**H**) female, forewing; (**I**) female, hind wing. Scale bar = 0.5 mm.

**Figure 28:**
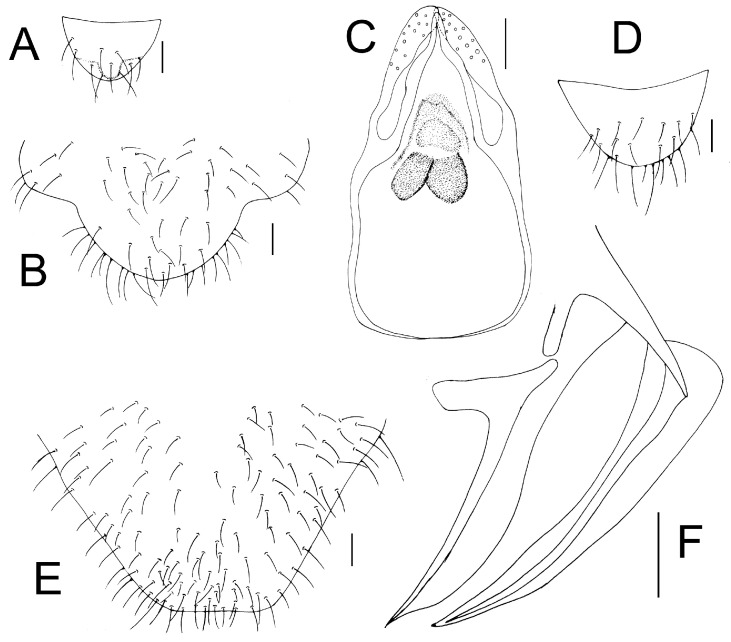
Drawing of *Neostenopsocus zonatus*: (**A**) male, epiproct; (**B**) male, hypandrium; (**C**) male, endophallus; (**D**) female, epiproct; (**E**) female, subgenital plate; (**F**) female, gonapophyses. Scale bar = 0.05 mm.

**(14) *Neostenopsocus zonatus* (Li, 1989) (**Figure 27 and Figure 28**)**

*Stenopsocus zonatus* Li, 1989 [39]: 32. Type locality: China (Shaanxi: Foping).

*Stenopsocus angustistriatus* Li, 2002 [2]: 665. Type locality: China (Guizhou: Pingtang). **syn. nov.**

*Stenopsocus genostictus* Li, 2002 [2]: 690. Type locality: China (Yunnan: Ruili). **syn. nov.**

*Stenopsocus macrocheirus* Li, 2002 [2]: 704. Type locality: China (Gansu: Wenxian). **syn. nov.**

*Stenopsocus thermophiles* Li, 2002 [2]: 705. Type locality: China (Guizhou: Luodian). **syn. nov.**

*Stenopsocus xiangxiensis* Li, 1992 [41]: 691. Type locality: China (Hunan: Zhangjiajie). **syn. nov.**

**Diagnosis**. This species is characterized by a yellowish vertex, dark brown antenna with yellowish-brown pedicel and scape, posterior margin of pterostigma with narrow brown stripe, and weakly sclerotized terminalia.

**Redescription.** Adult male: Body (Figure 27A) length 2.67 mm, length from postclypeus to wing tip 4.72 mm. IO: 0.37 mm; d: 0.26 mm; IO/d = 1.43; f1: 1.23 mm; f2: 1.17 mm; f3: 0.65 mm; FWL: 3.88 mm; FWW: 1.34 mm; HWL: 2.63 mm; HWW: 0.85 mm; t1: 0.39 mm; t2: 0.12 mm.

Color (in alcohol): Head (Figure 27B) yellowish brown, vertex (Figure 27C) yellowish. Antenna dark brown. Mouthparts yellowish white, with brown postclypeus. Thorax dark brown. Leg with yellowish-white femur and yellowish-brown tibia. Abdomen yellowish white. Genital segments yellowish white with pale brown paraproct.

Forewing (Figure 27D) transparent. Posterior margin of pterostigma with narrow brown marking. Hindwing (Figure 27E) immaculate.

Genital segments weakly sclerotized. Epiproct (Figure 28A) subtriangular. Paraproct sclerotized, with 44 trichobothria. Hypandrium (Figure 28B) without distinctly sclerotized area. Endophallus (Figure 28C) sclerotized, external parameres robust, with some punctures on broad end toward apex and exceeding apex of aedeagal arch.

Adult female: Body (Figure 27E) length 3.32 mm, length from postclypeus to wing tip 5.86 mm. IO: 0.44 mm; d: 0.20 mm; IO/d = 2.20; f1: 1.10 mm; f2: 1.07 mm; f3: 0.78 mm; FWL: 4.59 mm; FWW: 1.50 mm; HWL: 3.26 mm; HWW: 1.00 mm; t1: 0.42 mm; t2: 0.13 mm.

Color (in alcohol): Head (Figure 27F,G) brown. Frontal area with a pair of yellowish areas. Antenna dark brown. Mouthparts yellowish, with dark brown postclypeus. Prothorax yellowish brown, mid- and metathorax brown. Leg yellowish, hindleg with yellowish-brown tibia. Abdomen yellowish, genital segments yellowish with pale brown paraproct.

Forewing (Figure 27H) transparent. Anterior area of pterostigma yellowish, posterior area of pterostigma with dark brown stripe. Hindwing (Figure 27I) with a pale brown stripe between Sc and R.

Genital segments weakly sclerotized. Epiproct (Figure 28D) subtriangular. Paraproct with 28 trichobothria. Subgenital plate (Figure 28E) without distinctly sclerotized area. Gonapophyses (Figure 28F) weakly sclerotized.

**Specimens examined. Paratype** of *S*. *angustistriatus*, ♀, China, Guizhou, Guiyang, Huaxi (1000), 1981.VI.9, Fasheng Li (CAU) (Figure S39); **Holotype** of *S*. *genostictus*, ♀, China, Yunnan, Ruili, Jiegao (750 m), 1981.V.5, Chikun Yang (CAU) (Figure S40); **Holotype** of *S*. *macrocheirus*, ♀, China, Gansu, Wenxian, 1980.VIII.7, Fasheng Li (CAU) (Figure S41); **Paratype** of *S*. *thermophiles*, ♀, China, Gansu, Kangxian, Lianghe (800 m), Chikun Yang (CAU) (Figure S42); **Holotype** of *S*. *xiangxiensis*,♀, China, Hunan, Zhangjiajie (600–1500 m), 1985.X.13, Fasheng Li (CAU) (Figure S43).

CHINA: 2♂♂2♀♀, Shaanxi, Zhouzhi, Houzhenzi (1278 m), 2014.VII.17, Xiumei Lu (CAU); 2♀♀, Shaanxi, Lingwu, 2013.VII.13, Wanzhi Cai & Jianyun Wang (CAU); ♂, Shaanxi, Foping, Taiguping (1269 m), 2014.VIII.23, Xiumei Lu (CAU); 2♂♂2♀♀, Yunnan, Malipo, Xiajinchang (1430 m), 2016.VII.27, Yulan Jiang (CAU); 1♀, Guizhou, Luodian (850 m), 1981.VIII.8, Fasheng Li (CAU); 1♂1♀, Yunnan, Nanjian, Mt. Wuliangshan (2221 m), 2016.VII.17, Qicheng Yang (CAU); 1♀, Yunnan, Jinping, Maandi (1020 m), 2016.VII.13 Ya’nan Lv; VIETNAM: 1♀, Kon Tum, Chu Mom Ray National Park, 2012.VIII.1, Feiyang Liang (CAU).

**Distribution.** China (Guizhou, Yunnan, Guangdong, Guangxi, Shaanxi, Hunan), Vietnam (Kon Tum).

**Remarks.** There are intraspecific differences in the head markings of females, which are mainly displayed in the different brown areas in the frontal and genal regions. Combined with morphological characters and molecular species identification characters, *N. angustistriatus*, *N. genostictus*, *N. macrocheirus*, *N. thermophilus*, and *N. xiangxisnsis* are considered synonyms of *N. zonatus*. In addition, we also matched the male and female of this species based on the DNA barcoding. There are differences in marking patterns of forewing pterostigma between females and males of this species. The posterior margin of the pterostigma on the forewings of females bears a narrow brown band, whereas males have no obvious markings on their pterostigma. This species can be distinguished from *N. dictyodromus* by the yellowish scape and pedicel and the weakly sclerotized terminalia.

## 4. Discussion

### 4.1. Molecular Species Delimitation of Neostenopsocus

Building upon the limited analyses by Liang et al. [20], this study significantly increases the sample size and investigates species identification within *Neostenopsocus*. It demonstrates a strong concordance between the molecular species delimitation and morphological classification, confirming the utility of DNA barcoding for species identification in this genus. However, several groups failed to be delimitated based on the COI gene, i.e., *N. formosanus*–*N. externus* and *N. capacimacularus*–*N. melanocephalus* (Figure 1). The minimal interspecific K2P distance between *N. formosanus* and *N. externus* (0.0051) may reflect either a genuine genetic affinity or insufficient COI resolution. This ambiguity necessitates validation via nuclear gene sequencing to assess the incomplete lineage sorting. Additionally, expanded sampling of *N. formosanus* across Taiwan Island is critical for delimitating these two species. For the *N. capacimacularus*–*N. melanocephalus* clade, a detectable genetic divergence exists between these taxa, yet it remains insufficient to support their specific status in molecular delimitation analyses. This could be attributable to limited geographic or population coverage.

Molecular species delimitation studies based on DNA barcoding typically propose genetic distance thresholds for species identification. Although this study could not establish a definitive threshold, the K2P distance analyses of inter- and intraspecific comparisons suggest that a 0.08 minimum genetic distance may mitigate over-splitting for most *Neostenopsocus* species in biodiversity assessments. This threshold differs from the interspecific genetic distance of 0.02 previously suggested by Liang et al. [20] for the family Stenopsocidae. The primary reason for this difference is that Liang et al. [20] excluded the third codon position from their analysis, whereas our study, consistent with current practices, included all positions. In recent insect molecular species delimitation studies, nucleotide saturation analyses have often been omitted, with all codon positions directly utilized, as was performed in our study [9,10,11,12]. Notably, our dataset included the *Neostenopsocus* data generated by Liang et al. [20], and the species delimitation results for these taxa in our study are consistent with theirs. Therefore, we propose that a genetic distance threshold of 0.08 is more appropriate than 0.02. For specimens exhibiting genetic distances below 0.08, integrative approaches incorporating nuclear loci, morphological characters, and geographical data are recommended for conclusive identification.

### 4.2. External Diagnostic Characters of Neostenopsocus

In previous studies, there has been a lack of research on the morphology of Stenopsocidae. Liang & Liu [5] conducted intergeneric morphological comparisons within this family. Saville [8] reported *Stenopsocus immaculatus* to have variable marking patterns on the vertex and measurements of characters (i.e., ratio of interocular distance to eye diameter in dorsal view). Li [2] described over 90 *Neostenopsocus* species from China, proposing the vein length ratios in the forewing as the key diagnostic characters. However, these ratios have limited utility for species identification, as individual variations in forewing venation prevents it from serving as a reliable diagnostic character. Li [2] may have over-split Stenopsocidae species on the assumption that their dispersal capacity is limited. The dispersal ability of Stenopsocidae insects may far exceed Li’s estimates, as evidenced by the wide distribution of *Graphopsocus cruciatus* across the Northern Hemisphere [7,47]. In this study, we also found that several *Neostenopsocus* species have a relatively wide distribution, e.g., *N. anthracinus* and *N. externus* occur across over a dozen provinces in China. Furthermore, southwestern China likely harbors the highest species richness of *Neostenopsocus* based on this study.

For morphologically based insect species delimitation, genital morphology frequently provides critical diagnostic characters. However, it is difficult to evaluate the value of the genitalia for *Neostenopsocus* species delimitation because of its relatively conservative structure among species. The external genital structures—epiproct, paraprocts, and hypandrium/subgenital plate—are morphologically simple, without spines, hooks, or setae; their coloration varies with the degree of sclerotization, ranging from yellowish white to dark brown. The male phallosome, comprising parameres, aedeagus, phallobase, and endophallus, shows constrained interspecific morphological divergence, consequently providing limited value for *Neostenopsocus* species delimitation. In contrast, the female gonapophyses comprise three pairs of simple valves lacking spines, hooks, or setae; the intraspecific variation in these valves constrains their taxonomic reliability for species-level identification. Notably, a similar pattern occurs in the family Caeciliusidae, which has a close relationship with Stenopsocidae. Mockford [48] noted that the genus *Caecilius* exhibits conservative genital characters, yet certain species can still be reliably identified by their wing marking patterns.

In the genus *Neostenopsocus*, the body pigmentation patterns generally range from pale yellow (e.g., *N. kunmingiensis*) to dark brown (e.g., *N. melanocephalus*) in color, and can be used to identify *Neostenopsocus* species. The conspecific adults show minimal variation in body pigmentation, with no observable polymorphic patterns among the examined specimens. The following parts require careful attention to their coloration.

(1) Head excluding mouthparts and antennae: This part ranges from yellowish brown to dark brown in color. In some species with dark coloration, the head color is relatively stable, e.g., *N. maximalis* and *N. capacimacularus*. In *N. maximalis*, this part is entirely dark brown (Figure 21). *N. capacimacularus* shows a uniformly dark brown head except for the yellowish-white vertex and mouthparts (Figure 4). In contrast, intraspecific variability is evident in some species. For instance, the frons of *N. dictyodromus* is yellow with brown marking, which is obviously variable among individuals.

(2) Antennae: The antennal scape and pedicel vary from yellowish brown to blackish brown. Although this color variation is species-specific, it is insufficiently diagnostic and should not be used as a primary feature for species identification. The antennal flagellum is generally brown to dark brown, while in some species the apical several flagellomeres are whitish. For instance, the antennae of *N. anthracinus* have seven brown flagellomeres basally and four whitish ones apically (Figure 2). This characteristic is found only in females, while males lack it, and it can serve as an interspecific diagnostic feature, though its utility is limited in *Neostenopsocus* due to the fragility of antennal apices and consequent preservation challenges.

(3) Forewing: A brown marking near the pterostigma of the forewing is generally present in many species of *Neostenopsocus*, and its shape is considered a species diagnostic character. In several species, this marking is obviously large and reliable in each individual, such as *N. makii* and *N. maximalis* (Figure 21). Additionally, *N. externus*, widely distributed across southern China, shows the highly consistent marking pattern of pterostigma in individuals from different localities. However, intraspecific variation in marking of pterostigma occurs in certain taxa. This marking in *N. capacimacularus* exhibits two discrete morphotypes (Figure 4). Moreover, the individuals of *N. dictyodromus* collected from a single locality (Yinshan Park, Guangxi) displayed continuous variations in this marking (Figure 8).

(4) Leg: The variation in leg coloration occurs primarily on the tibiae. In *Neostenopsocus*, the tibiae range from yellowish to dark brown, exhibiting differences both interspecifically and intraspecifically, and therefore are not reliable for species identification.

(5) Abdomen: The coloration of abdomen generally shows yellowish white or reddish purple. In some species, the dorsal abdomen is reddish purple in live or relatively fresh specimens. However, this color fades away after prolonged preservation in alcohol.

Although the coloration and markings show some species-level differences, these alone are insufficient for reliably distinguishing between closely related species. *Neostenopsocus* species exhibit limited interspecific variation in their external morphology, and given this remarkable uniformity, deeper morphological insights may require complementary techniques—including SEM imaging of ultrastructure, micro-CT-based 3D reconstruction of internal anatomy, and geometric morphometric analyses of wings and their markings.

## 5. Conclusions

In this study, we redescribe 13 species of *Neostenopsocus* from China, and propose 39 new synonyms by integrating morphology with DNA barcoding. The results corroborate the utility of COI for species delimitation within the genus; nevertheless, for closely similar taxa, additional molecular markers must be combined with ecological and distributional evidence to achieve reliable identification. Further field surveys and specimen collection are also imperative for this poorly known group. In addition, the morphology of Stenopsocidae demands deeper investigation; applying modern morphological techniques will provide reliable and accurate morphological data for both species identification and evolutionary studies of psocids.

## Data Availability

The sequences obtained in this study were deposited in GenBank with accession numbers PX208573-PX208653.

## Supplementary Materials

The following supporting information can be downloaded at: https://www.mdpi.com/article/10.3390/insects16111147/s1, Alignment S1: Alignment of COI + outgroup dataset; Table S1: Sampling information and GenBank accession numbers/BOLD sample IDs used in this study; Table S2: Interspecific K2P genetic distances (COI gene fragments) between these 20 species of *Neostenopsocus*; Table S3: Intraspecies mean K2P distances (COI gene fragments) within these 20 species of *Neostenopsocus*; Table S4: Pairwise K2P genetic distances (COI gene fragments) between 110 individuals of 20 species of *Neostenopsocus*; Figure S1: Bayesian phylogenetic tree of COI DNA barcodes for *Neostenopsocus*; Figure S2: Neighbor-joining tree of COI DNA barcodes for *Neostenopsocus*; Figure S3: Photographs of holotype of *Stenopsocus anthracinus*; Figure S4: Photographs of holotype of *Stenopsocus angustifurcus*; Figure S5: Photographs of holotype of *Stenopsocus biconvexus*; Figure S6: Photographs of holotype of *Stenopsocus bipunctatus*; Figure S7: Photographs of holotype of *Stenopsocus cassideus*; Figure S8: Photographs of holotype of *Stenopsocus dichospilus*; Figure S9: Photographs of holotype of *Stenopsocus flavifrons*; Figure S10: Photographs of holotype of *Stenopsocus fulivertex*; Figure S11: Photographs of holotype of *Stenopsocus frontalis*; Figure S12: Photographs of holotype of *Stenopsocus parviforficatus*; Figure S13: Photographs of holotype of *Stenopsocus podorphus*; Figure S14: Photographs of holotype of *Stenopsocus qianipullus*; Figure S15: Photographs of holotype of *Stenopsocus symipsarous*; Figure S16: Photographs of holotype of *Stenopsocus capacimacularus*; Figure S17: Photographs of holotype of *Stenopsocus dictyodromus*; Figure S18: Photographs of holotype of *Stenopsocus longitudinalis*; Figure S19: Photographs of holotype of *Stenopsocus trisetus*; Figure S20: Photographs of holotype of *Stenopsocus eucallus*; Figure S21: Photographs of holotype of *Stenopsocus metastictus*; Figure S22: Paragraph of type specimens of *Stenopsocus externus*; Figure S23: Photographs of holotype of *Stenopsocus hemiostictus*; Figure S24: Photographs of holotype of *Stenopsocus phaneostriatus*; Figure S25: Photographs of holotype of *Stenopsocus foliaceus*; Figure S26: Photographs of holotype of *Stenopsocus hexagonus*; Figure S27: Photographs of holotype of *Stenopsocus maculosus*; Figure S28: Photographs of holotype of *Stenopsocus brevicapitus*; Figure S29: Photograph of holotype of *Stenopsocus dactylinus*; Figure S30: Photographs of holotype of *Stenopsocus daozheniensis*; Figure S31: Photographs of holotype of *Stenopsocus isotomus*; Figure S32: Photographs of holotype of *Stenopsocus perspicuus*; Figure S33: Photographs of holotype of *Stenopsocus shennongjiaensis*; Figure S34: Photographs of holotype of *Stenopsocus wuxiaensis*; Figure S35: Photographs of holotype of *Stenopsocus xilingxianicus*; Figure S36: Photographs of holotype of *Stenopsocus maximalis*; Figure S37: Photographs of holotype of *Stenopsocus melanocephalus*; Figure S38: Photograph of holotype of *Stenopsocus polyceratus*; Figure S39: Photographs of Paratype of *Stenopsocus angustistriatus*; Figure S40: Photographs of holotype of *Stenopsocus genostictus*; Figure S41: Photographs of holotype of *Stenopsocus macrocheirus*; Figure S42: Photographs of paratype of *Stenopsocus thermophile*; Figure S43: Photographs of holotype of *Stenopsocus xiangxiensis*.
