# Supplementary material for: Species Delimitation in the Bark Louse Genus Neostenopsocus Liang & Liu, 2024 (Psocodea: Stenopsocidae) Based on DNA Barcoding
Source: Insects. 2025 Nov 9;16(11):1147. doi: 10.3390/insects16111147 (PMC12653511; doi:10.3390/insects16111147)
Supplement: Supplementary file 1 [file insects-16-01147-s001.zip › Table S1.pdf]

Table S1. Sampling information and GenBank accession numbers/BOLD Sample IDs used in this study.

Sample code	Species	Locality	Genbank No.	BOLD ID
NanthracinusCQ	*N. anthracinus*	Chongqing, China	PX208575	
NanthracinusHB1	*N. anthracinus*	Hubei, China	PX208576	
NanthracinusHB2	*N. anthracinus*	Hubei, China	PX208577	
NanthracinusHB3	*N. anthracinus*	Hubei, China	PX208578	
NanthracinusHB4	*N. anthracinus*	Hubei, China	PX208579	
NanthracinusNP	*N. anthracinus*	Pokhara, Nepal	PX208580	
NanthracinusSC1	*N. anthracinus*	Sichuan, China	PX208581	
NanthracinusSC2	*N. anthracinus*	Sichuan, China	PX208582	
NanthracinusSC3	*N. anthracinus*	Sichuan, China	PX208583	
NanthracinusSX	*N. anthracinus*	Shaanxi, China	PX208584	
NanthracinusSX1	*N. anthracinus*	Shaanxi, China	PX208585	
NanthracinusSX2	*N. anthracinus*	Shaanxi, China	PX208586	
NanthracinusXZ	*N. anthracinus*	Xizang, China	PX208587	
NcapacimaculatusCQ	*N.capacimacularus*	Chongqing, China	PX208588	
NcapacimaculatusHN1	*N.capacimacularus*	Hunan, China	PX208589	
NcapacimaculatusHN2	*N.capacimacularus*	Hunan, China	PX208590	
NdictyodromusCQ	*N. dictyodromus*	Chongqing, China	PX208591	
NdictyodromusFJ	*N. dictyodromus*	Fujian, China	PX208592	
NdictyodromusGX	*N. dictyodromus*	Guangxi, China	PX208593	
NdictyodromusGX1	*N. dictyodromus*	Guangxi, China	PX208594	
NdictyodromusGX2	*N. dictyodromus*	Guangxi, China	PX208595	
NdictyodromusGX3	*N. dictyodromus*	Guangxi, China	PX208596	
NdictyodromusYN1	*N. dictyodromus*	Yunnan, China	PX208597	

NdictyodromusYN2	*N. dictyodromus*	Yunnan, China	PX208598
NdictyodromusYN3	*N. dictyodromus*	Yunnan, China	PX208599
NdictyodromusYN4	*N. dictyodromus*	Yunnan, China	PX208600
SeTW1	*N. externus*	Taiwan, China	KT805202.1
SeTW2	*N. externus*	Taiwan, China	KT805202.1
SeVT	*N. externus*	Kon Tum, Vietnam	KT805204.1
SeHN1	*N. externus*	Henan, China	KT805224.1
SeHN2	*N. externus*	Henan, China	KT805225.1
SeGX1	*N. externus*	Guangxi, China	KT805226.1
SeGX2	*N. externus*	Guangxi, China	KT805227.1
SeHB	*N. externus*	Hubei, China	KT805228.1
SeSH	*N. externus*	Shanghai, China	KT805229.1
NexternusXZ	*N. externus*	Xizang, China	PX208607
NeucallusGX	*N. eucallus*	Guangxi, China	PX208601
NeucallusSC	*N. eucallus*	Sichuan, China	PX208602
NeucallusXZ	*N. eucallus*	Xizang, China	PX208603
NeucallusYN1	*N. eucallus*	Yunnan, China	PX208604
NeucallusYN2	*N. eucallus*	Yunnan, China	PX208605
NeucallusYN3	*N. eucallus*	Yunnan, China	PX208606
NfoliaceusSC	*N. foliaceus*	Sichuan, China	PX208608
NfoliaceusSX	*N. foliaceus*	Shaanxi, China	PX208609
SfTW2	*N. formosanus*	Taiwan, China	KT805205.1
SfTW1	*N. formosanus*	Taiwan, China	KT805206.1
SfTW3	*N. formosanus*	Taiwan, China	KT805207.1
SfTW4	*N. formosanus*	Taiwan, China	KT805208.1

NhexagonusXZ1	*N. hexagonus*	Xizang, China	PX208610
NhexagonusXZ2	*N. hexagonus*	Xizang, China	PX208611
NkunmingensisYN1	*N. kunmingiensis*	Yunnan, China	PX208612
NkunmingensisYN3	*N. kunmingiensis*	Yunnan, China	PX208613
NkunmingensisYN4	*N. kunmingiensis*	Yunnan, China	PX208614
SmTW1	*N. makii*	Taiwan, China	KT805210.1
SmTW2	*N. makii*	Taiwan, China	KT805211.1
SmTW3	*N. makii*	Taiwan, China	KT805212.1
NmaximalisCQ	*N. maximalis*	Chongqing, China	PX208631
NmaximalisNP1	*N. maximalis*	Pokhara, Nepal	PX208632
NmaximalisNP2	*N. maximalis*	Pokhara, Nepal	PX208633
NmaximalisSC	*N. maximalis*	Sichuan, China	PX208634
NmaximalisYN	*N. maximalis*	Yunnan, China	PX208635
NmaculosusSC1	*N. maculosus*	Sichuan, China	PX208629
NmaculosusSC2	*N. maculosus*	Sichuan, China	PX208630
NmelanocephalusGX	*N. melanocephalus*	Guangxi, China	PX208636
NmelanocephalusSC	*N. melanocephalus*	Sichuan, China	PX208637
NnepalensisCQ	*N. nepalensis*	Chongqing, China	PX208638
NnepalensisNP1	*N. nepalensis*	Pokhara, Nepal	PX208639
NnepalensisNP2	*N. nepalensis*	Pokhara, Nepal	PX208640
NnepalensisNP3	*N. nepalensis*	Pokhara, Nepal	PX208641
NnepalensisSC	*N. nepalensis*	Sichuan, China	PX208642
NnepalensisVT	*N. nepalensis*	Lam Dong, Vietnam	PX208643
NnepalensisYN1	*N. nepalensis*	Yunnan, China	PX208644
NnepalensisYN2	*N. nepalensis*	Yunnan, China	PX208645

NnepalensisYN3	*N. nepalensis*	Yunnan, China	PX208646	
NnepalensisYN4	*N. nepalensis*	Yunnan, China	PX208647	
NnepalensisYN5	*N. nepalensis*	Yunnan, China	PX208648	
NnepalensisYN6	*N. nepalensis*	Yunnan, China	PX208649	
NpolyceratusSX1	*N. polyceratus*	Shaanxi, China	PX208650	
NpolyceratusSX2	*N. polyceratus*	Shaanxi, China	PX208651	
ACO9993	*N. polyceratus*	Shaanxi, China	-	BIOUG14417-G04
NpygmaeusJP	*N. pygmaeus*	Hokkaido, Japan	PX208652	
FPSO-000043	*N. stigmaticus*	Ostrobottnia ultima, Finland	MZ626940.1	
FPSO-000044	*N. stigmaticus*	Ostrobottnia ultima, Finland	MZ625496.1	
SHAP3782	*N. stigmaticus*	Unknown, UK	-	SHAP_023_A12
SHAP3785	*N. stigmaticus*	Unknown, UK	-	SHAP_023_B12
SHAP3786	*N. stigmaticus*	Unknown, UK	-	SHAP_023_C12
NwangiLAO	*N. wangi*	Oudomxai,Lao	PX208653	
Nzonatus CQ	*N. zonatus*	Chongqing, China	PX208615	
Nzonatus GX1	*N. zonatus*	Guangxi, China	PX208616	
Nzonatus GX2	*N. zonatus*	Guangxi, China	PX208617	
Nzonatus GX3	*N. zonatus*	Guangxi, China	PX208618	
Nzonatus GX4	*N. zonatus*	Guangxi, China	PX208619	
Nzonatus GX5	*N. zonatus*	Guangxi, China	PX208620	
Nzonatus SX1	*N. zonatus*	Shaanxi, China	PX208621	
Nzonatus SX2	*N. zonatus*	Shaanxi, China	PX208622	
Nzonatus SX3	*N. zonatus*	Shaanxi, China	PX208623	
Nzonatus SX4	*N. zonatus*	Shaanxi, China	PX208624	
Nzonatus SX5	*N. zonatus*	Shaanxi, China	PX208625	

Nzonatus YN1	*N. zonatus*	Yunnan, China	PX208626
Nzonatus YN2	*N. zonatus*	Yunnan, China	PX208627
Nzonatus ZJ	*N. zonatus*	Zhejiang, China	PX208628
MAL-GZF	*M. yunnanicus*	Guizhou, China	PX208573
MAL-HNF	*M. yunnanicus*	Hunan, China	PX208574
FPSO-000293	*S. immaculatus*	Regio aboensis, Finland	MZ627670.1
FPSO-000037	*S. immaculatus*	Karelia borealis, Finland	MZ624373.1
FPSO-000038	*S. immaculatus*	Ostrobottnia australis, Finland	MZ625976.1
FPSO-000264	*S. immaculatus*	Regio aboensis, Finland	MZ628856.1
GcAU1	*G. cruciatus*	Korneuburg, Austria	KT805222.1
GcAU1	*G. cruciatus*	Korneuburg, Austria	KT805223.1
BIOUG22679	*G. cruciatus*	Unknown, Canada	MG377714.1
BIOUG31052	*G. cruciatus*	Unknown, Canada	MG377858.1
BIOUG28488	*G. cruciatus*	Unknown, Canada	MG378233.1
BIOUG28270	*G. cruciatus*	Unknown, Canada	MG378245.1
