# Supplementary figures and images for: Species Delimitation in the Bark Louse Genus Neostenopsocus Liang & Liu, 2024 (Psocodea: Stenopsocidae) Based on DNA Barcoding
Source: Insects. 2025 Nov 9;16(11):1147. doi: 10.3390/insects16111147 (PMC12653511; doi:10.3390/insects16111147)

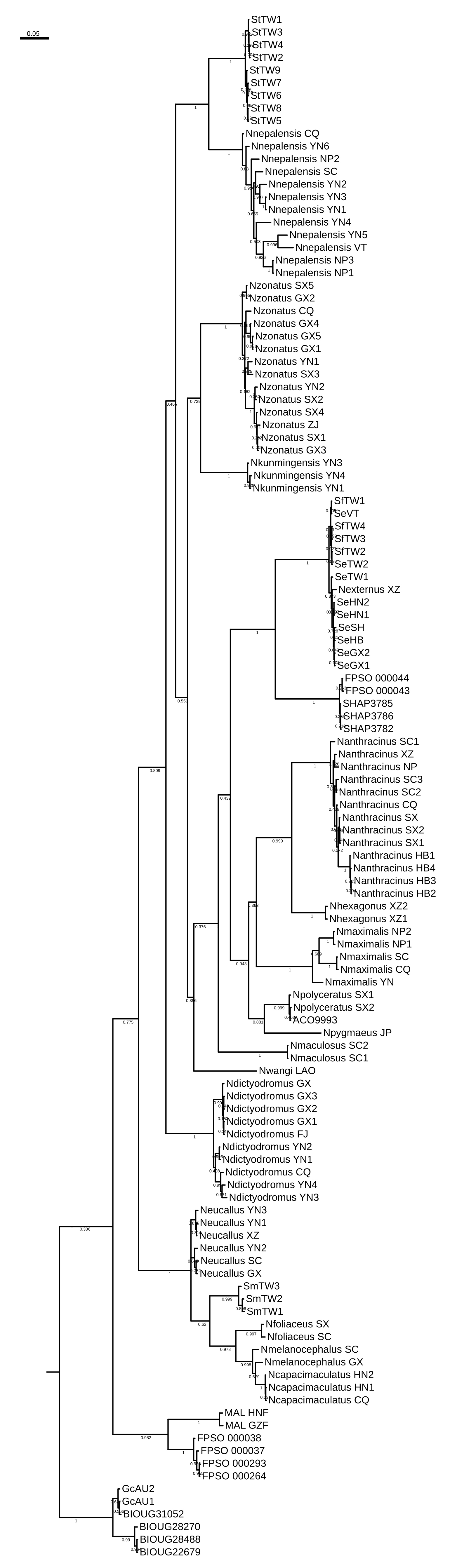

Supplement: Supplementary file 1 [file insects-16-01147-s001.zip › Figure S1 BItree.jpg]

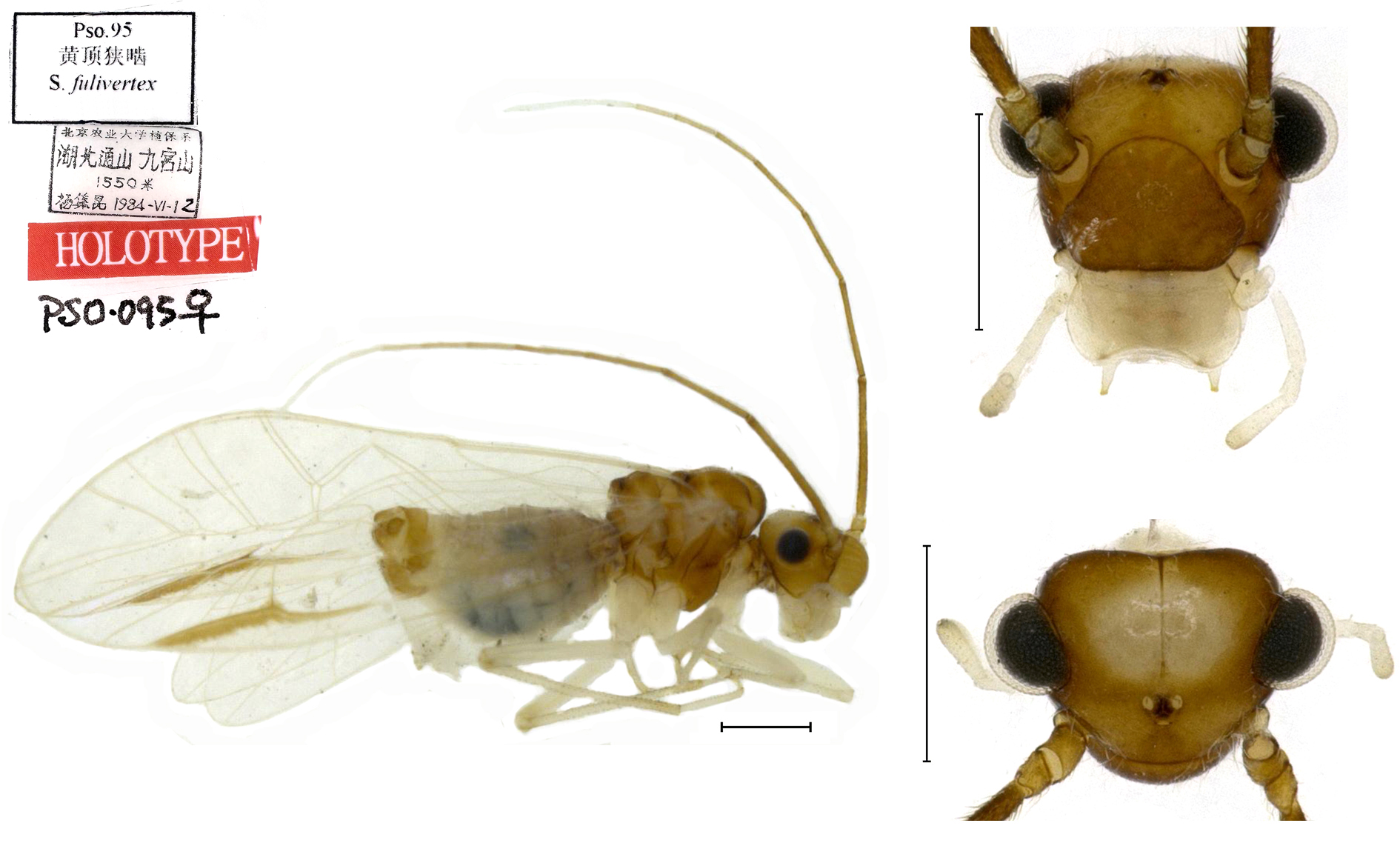

Supplement: Supplementary file 1 [file insects-16-01147-s001.zip › Figure S10 Holotype of Stenopsocus fulivertex.jpg]

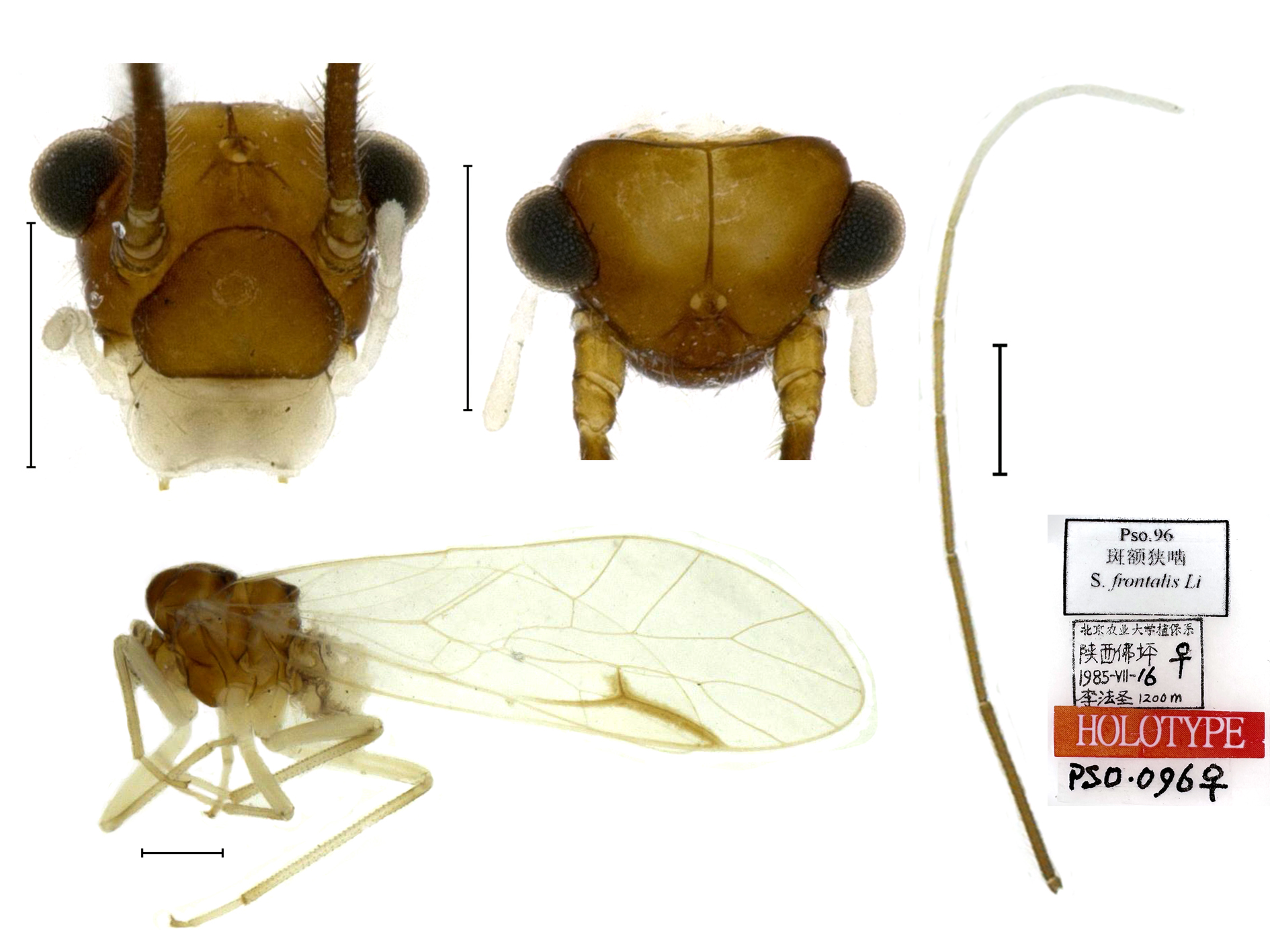

Supplement: Supplementary file 1 [file insects-16-01147-s001.zip › Figure S11 Holotype of Stenopsocus frontalis .jpg]

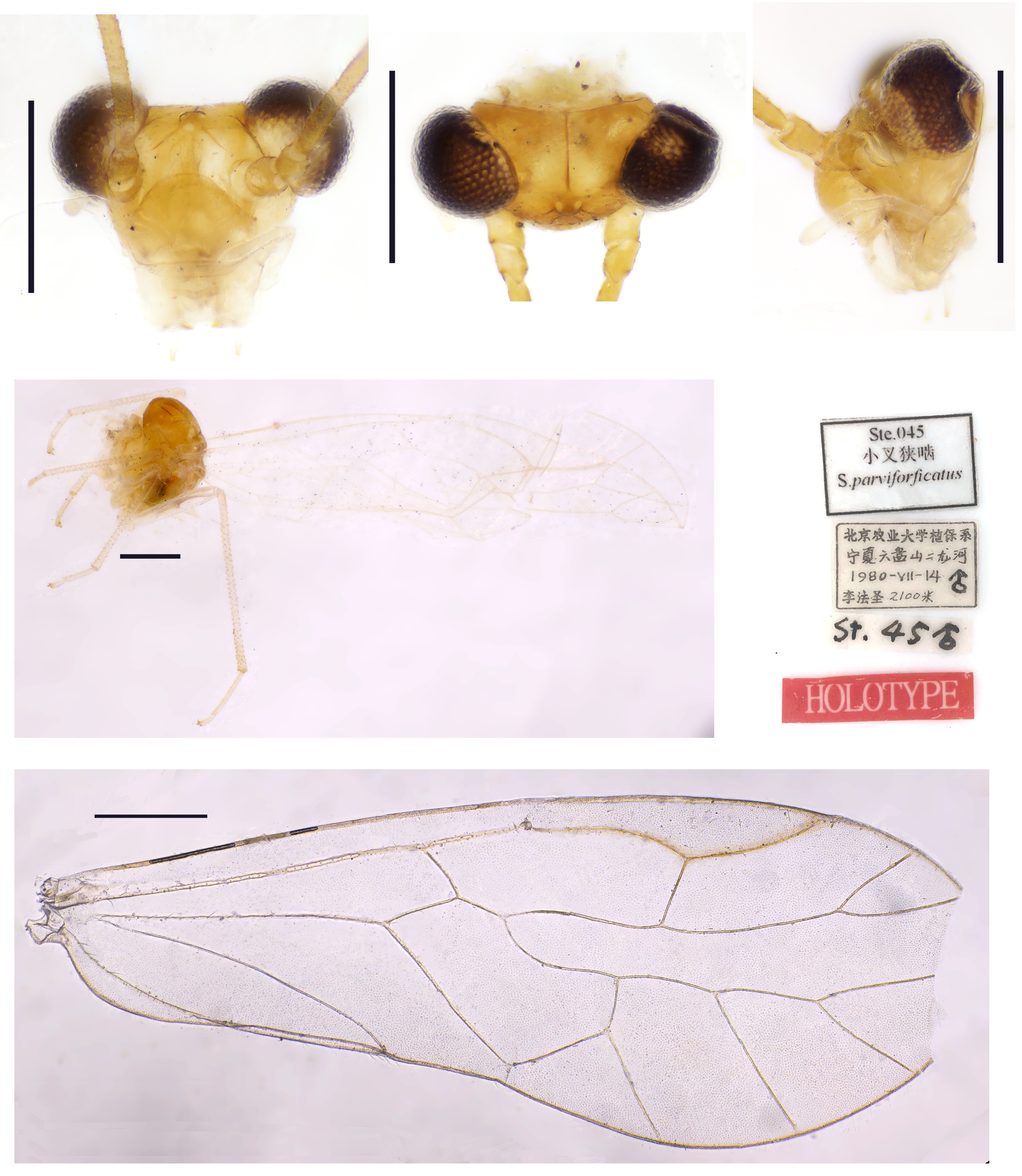

Supplement: Supplementary file 1 [file insects-16-01147-s001.zip › Figure S12 Holotype of Stenopsocus parviforficatus.jpg]

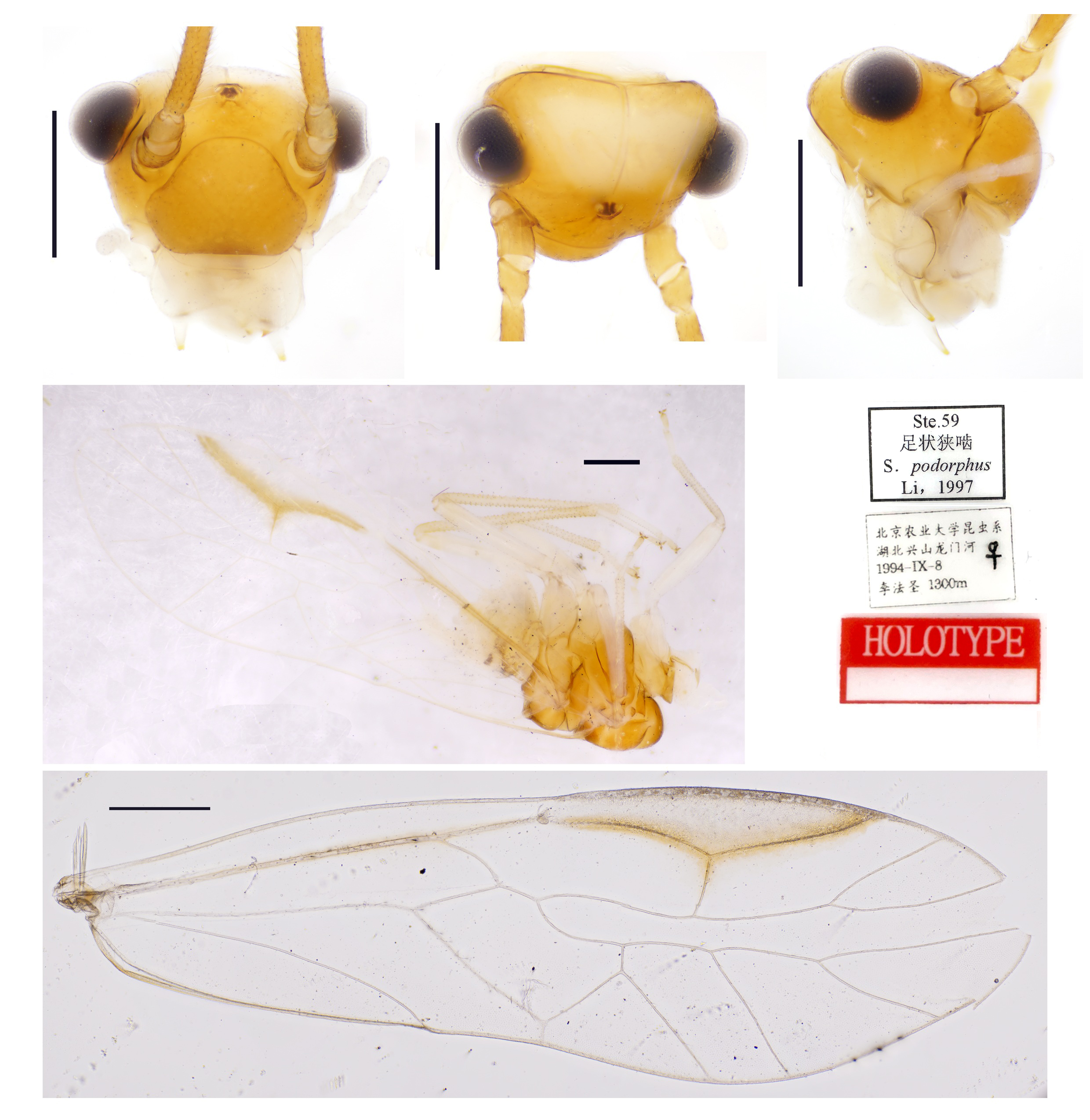

Supplement: Supplementary file 1 [file insects-16-01147-s001.zip › Figure S13 Holotype of Stenopsocus podorphus.jpg]

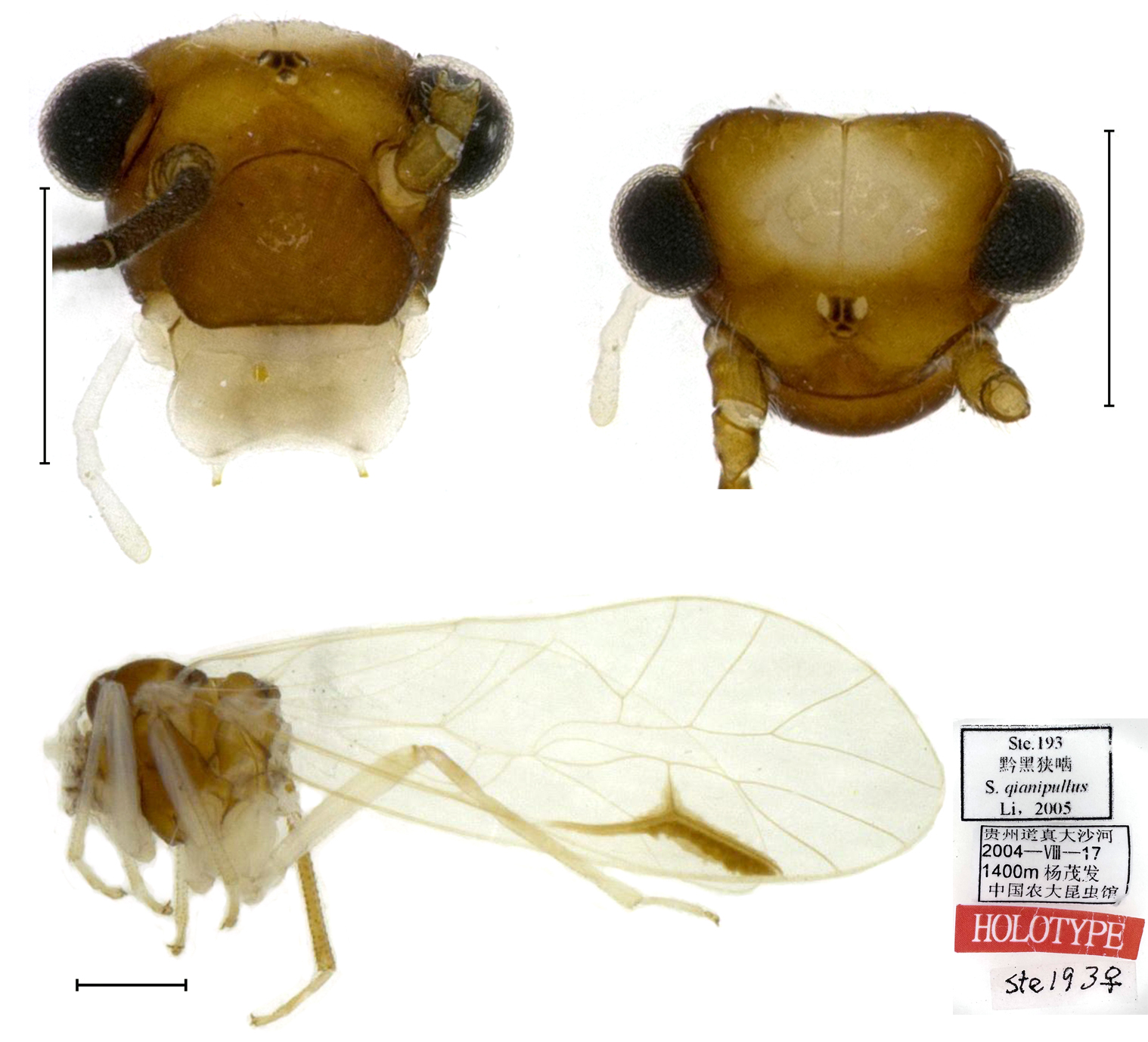

Supplement: Supplementary file 1 [file insects-16-01147-s001.zip › Figure S14 Holotype of Stenopsocus qianipullus.jpg]

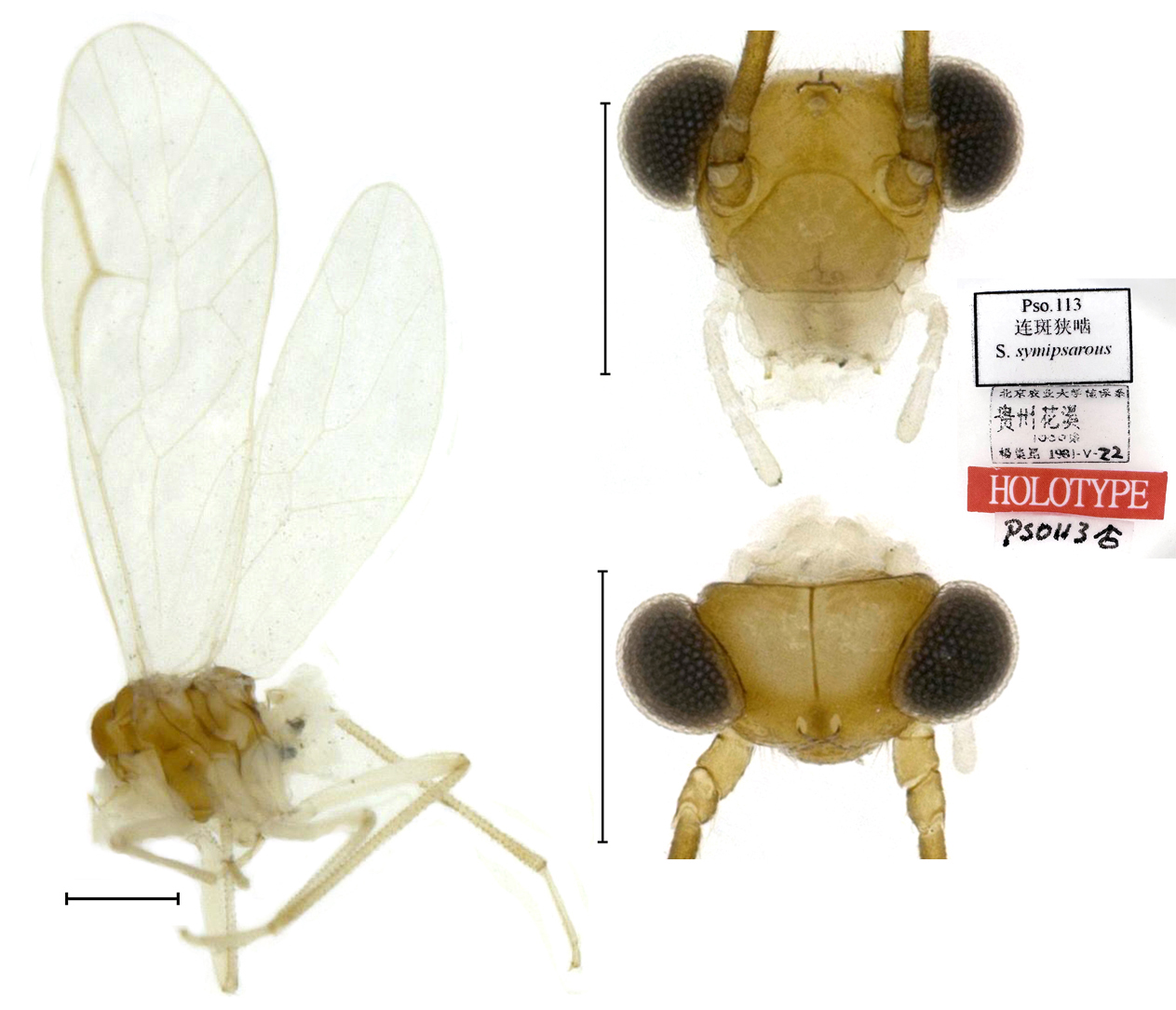

Supplement: Supplementary file 1 [file insects-16-01147-s001.zip › Figure S15 Holotype of Stenopsocus symipsarous.jpg]

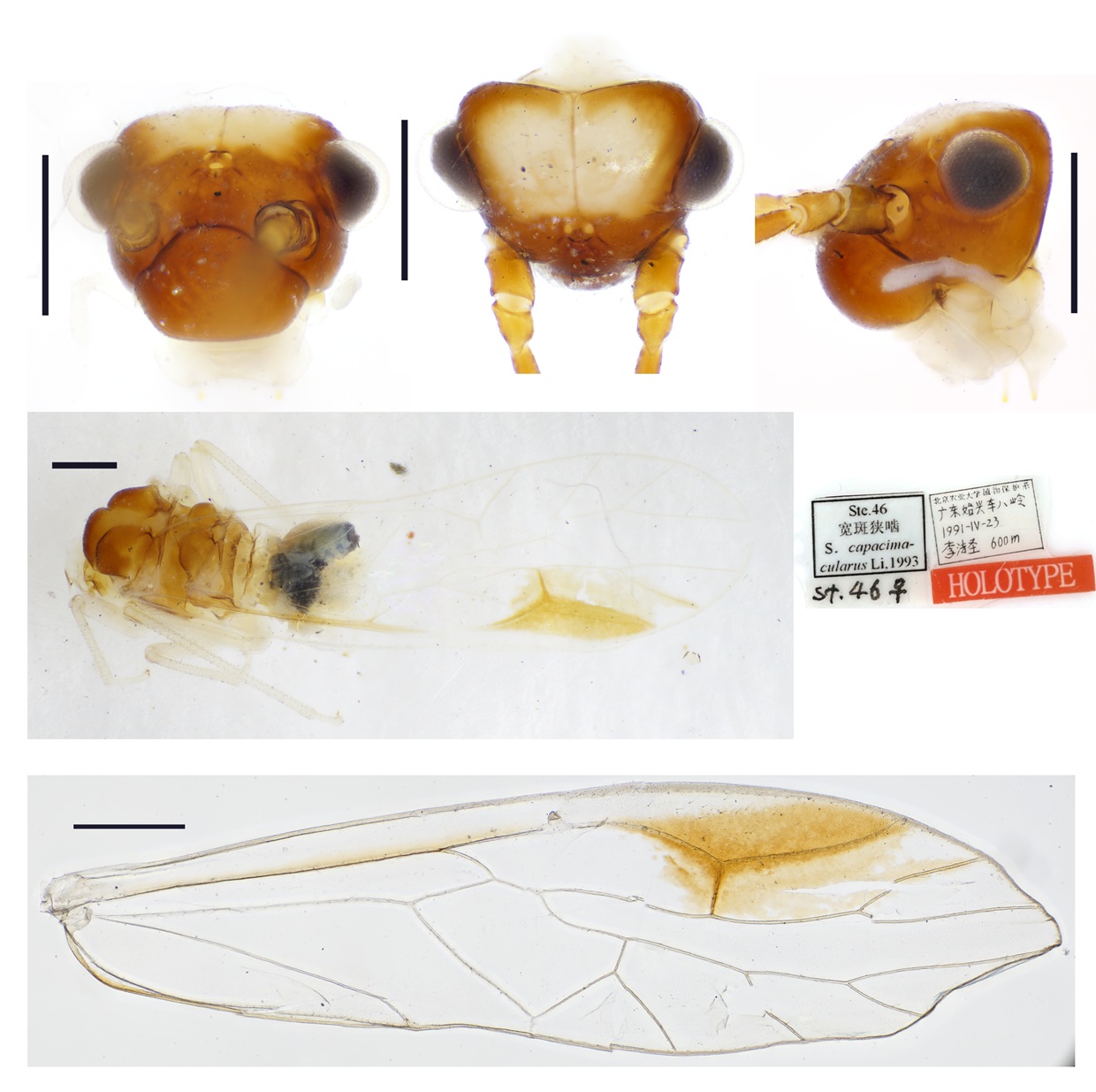

Supplement: Supplementary file 1 [file insects-16-01147-s001.zip › Figure S16 Holotype of Stenopsocus capacimacularus.jpg]

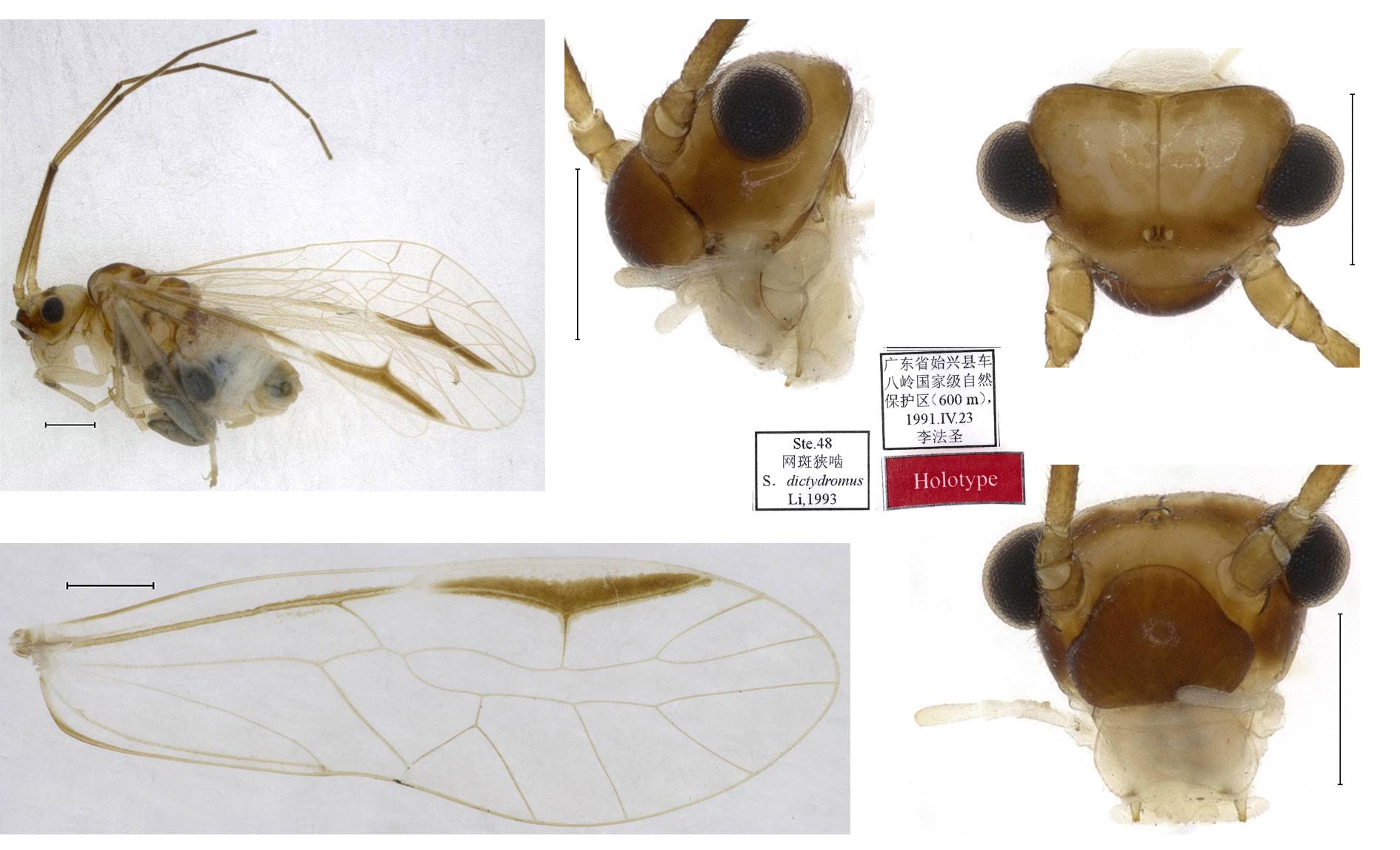

Supplement: Supplementary file 1 [file insects-16-01147-s001.zip › Figure S17 Holotype of Stenopsocus dictyodromus.jpg]

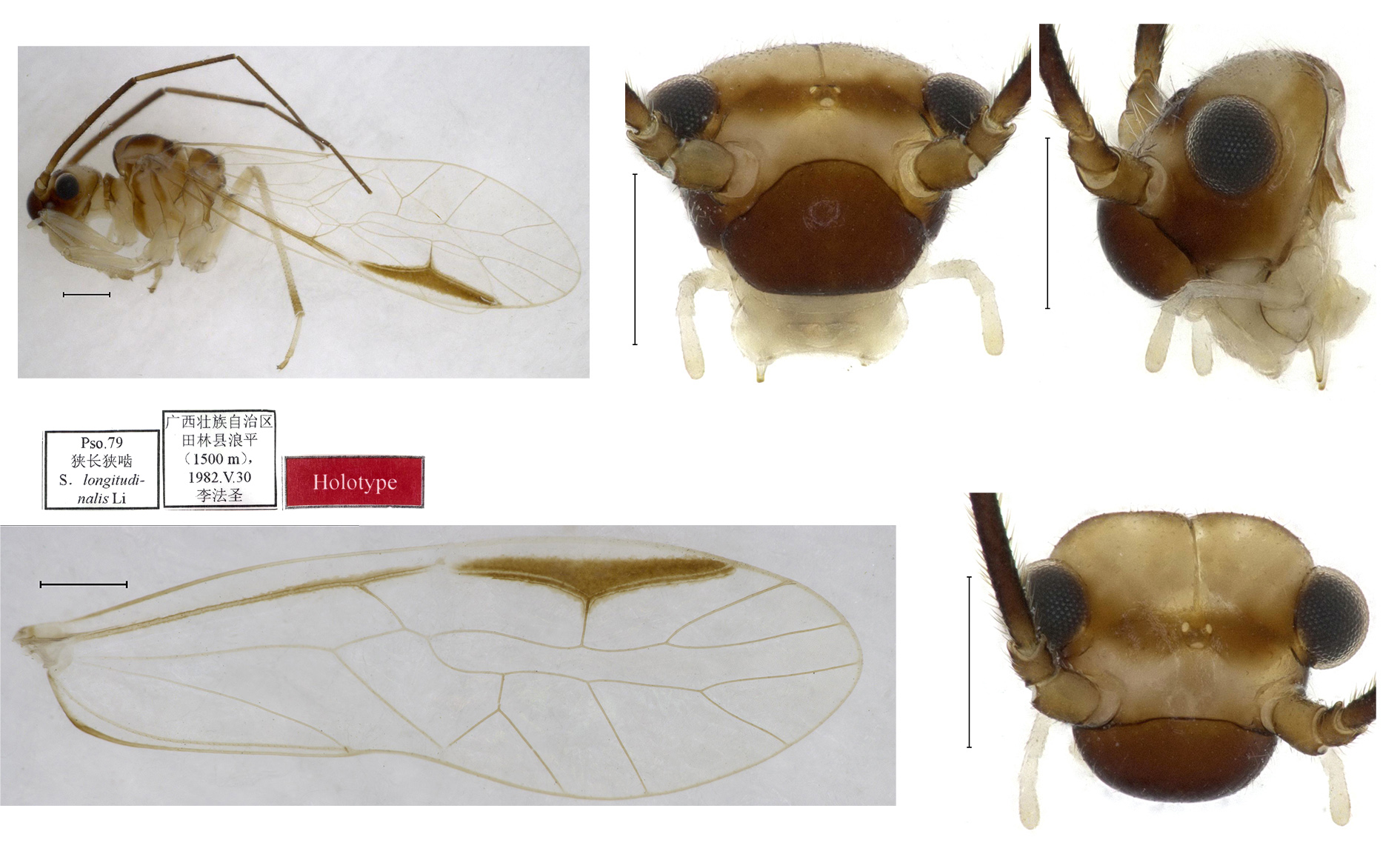

Supplement: Supplementary file 1 [file insects-16-01147-s001.zip › Figure S18 Holotype of Stenopsocus longitudinalis.jpg]

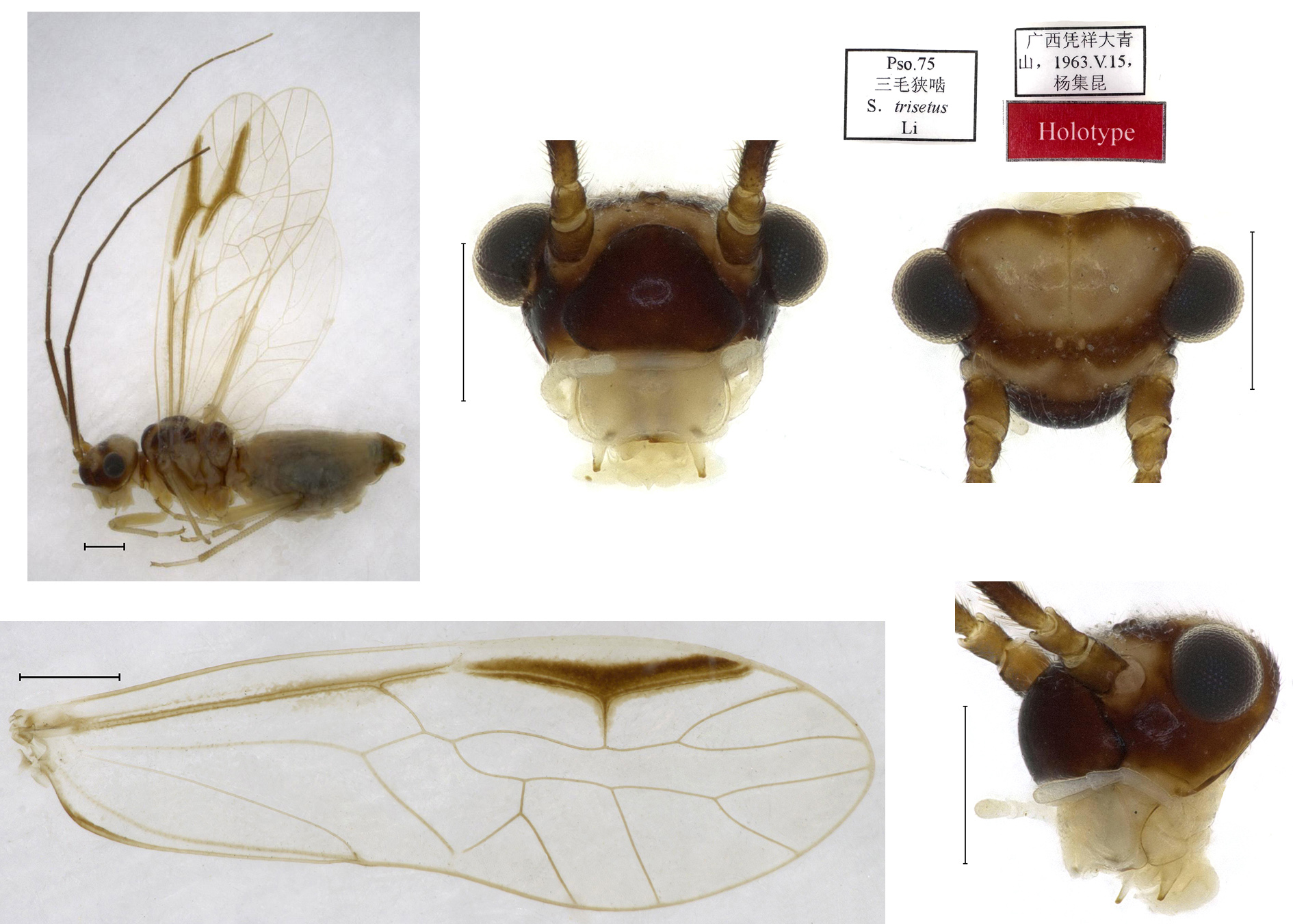

Supplement: Supplementary file 1 [file insects-16-01147-s001.zip › Figure S19 Holotype of Stenopsocus trisetus.jpg]

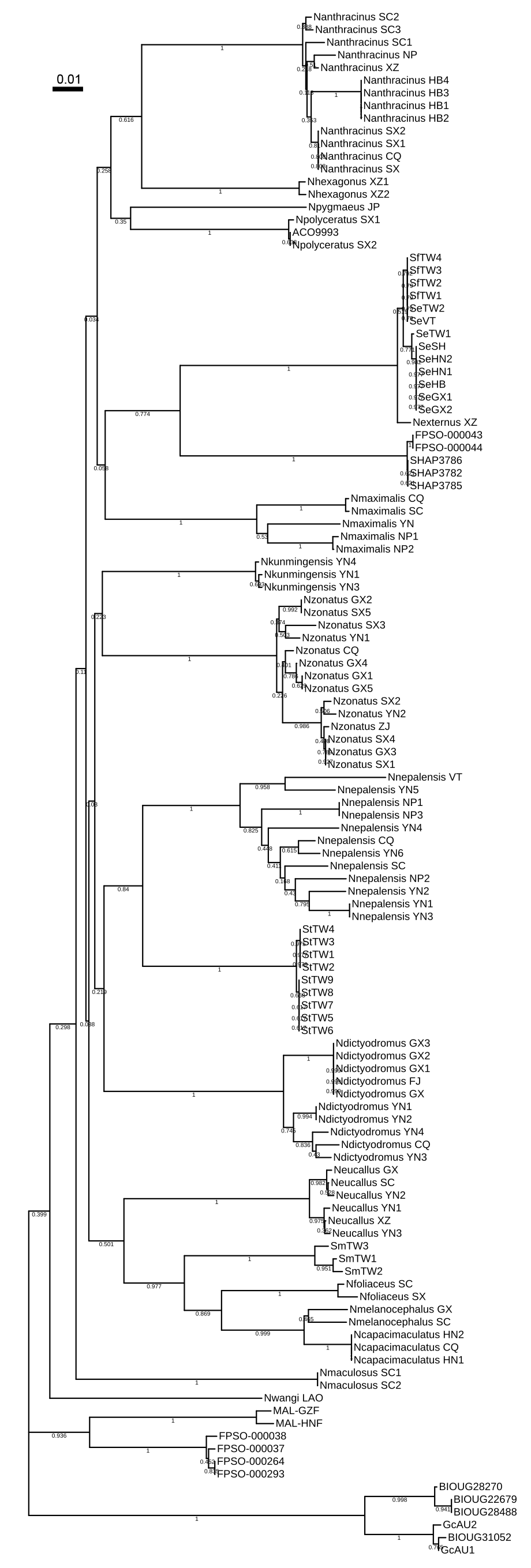

Supplement: Supplementary file 1 [file insects-16-01147-s001.zip › Figure S2 NJtreeNeo.jpg]

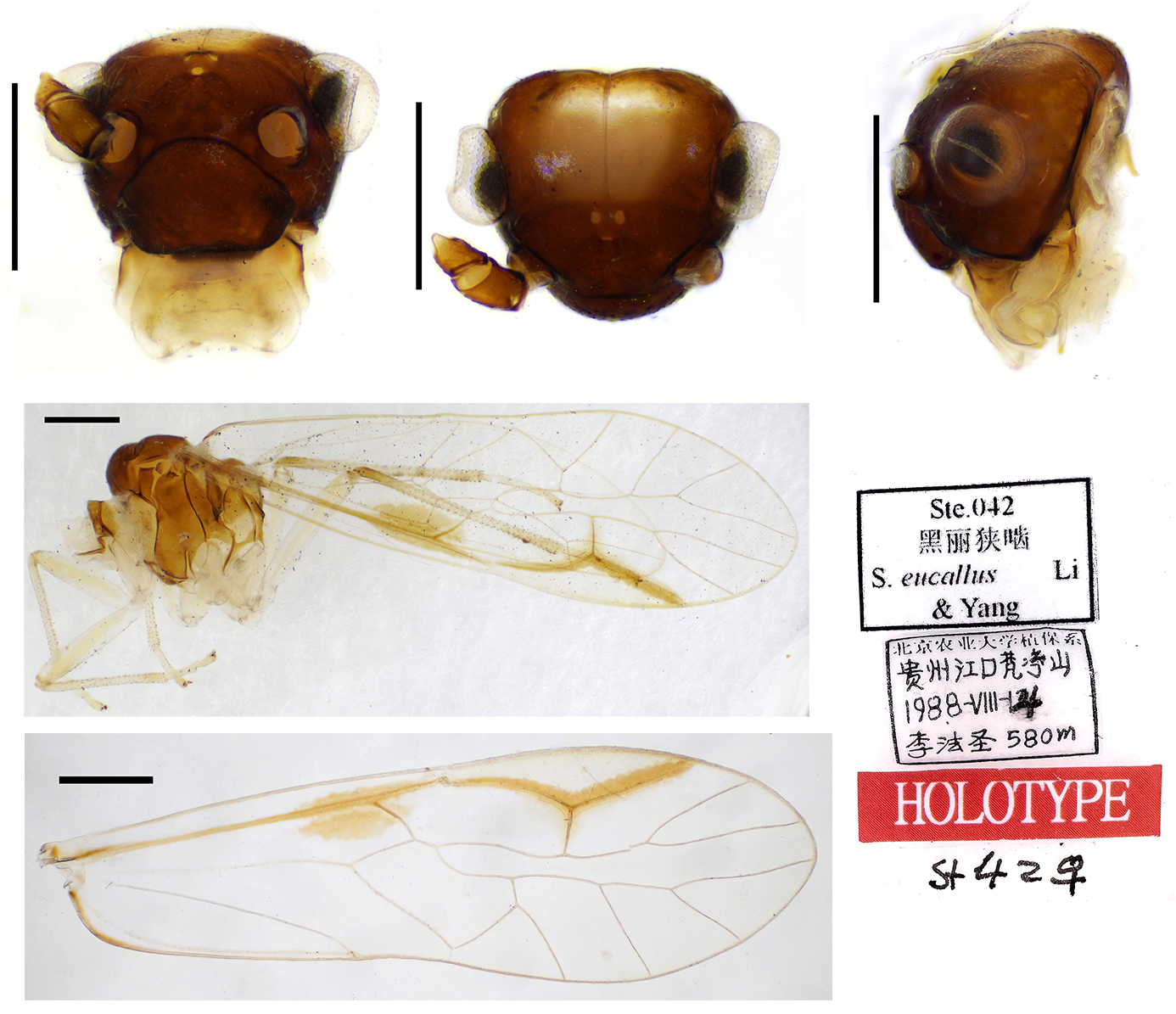

Supplement: Supplementary file 1 [file insects-16-01147-s001.zip › Figure S20 Holotype of Stenopsocus eucallus.jpg]

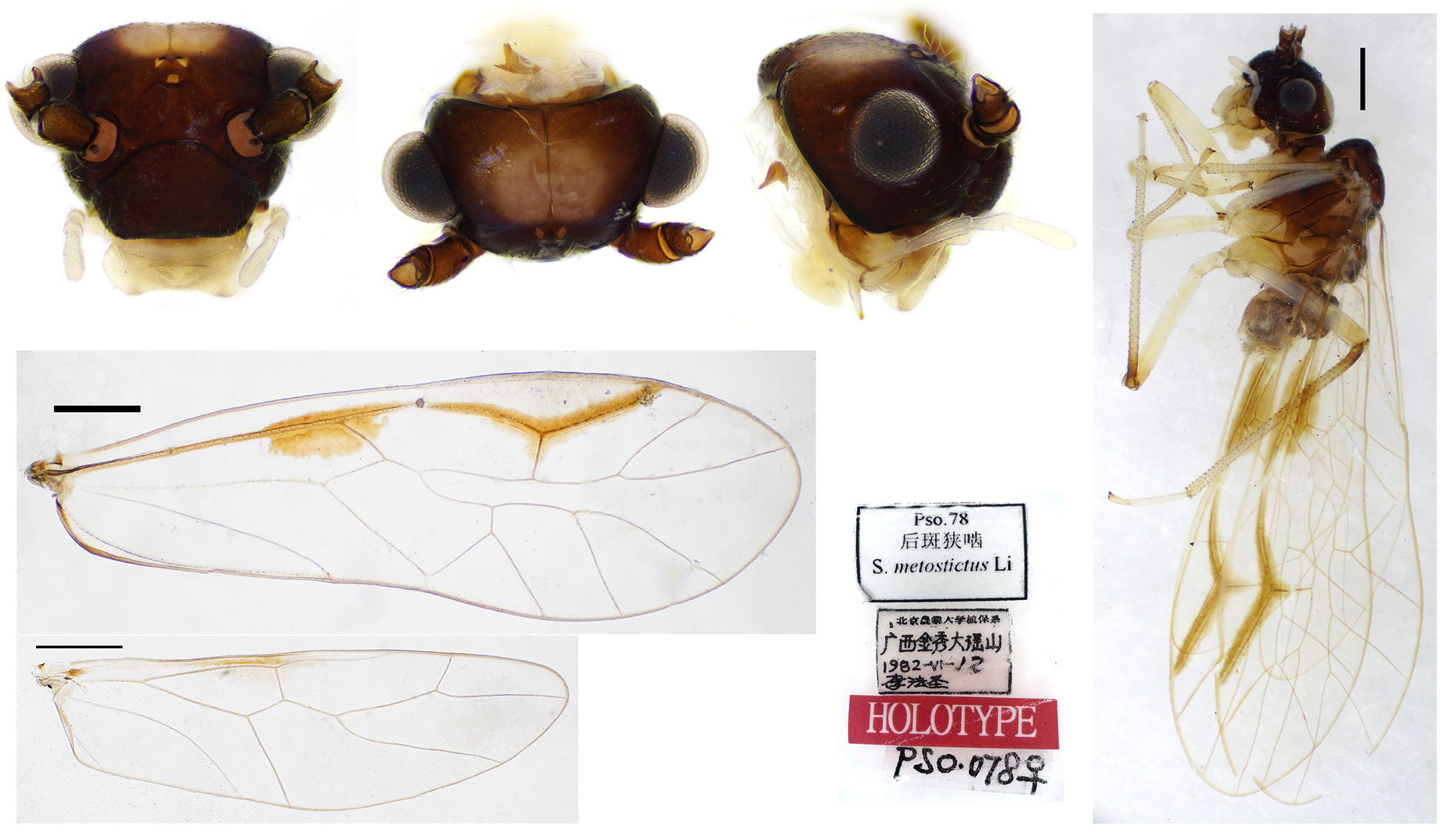

Supplement: Supplementary file 1 [file insects-16-01147-s001.zip › Figure S21 Holotype of Stenopsocus metastictus.jpg]

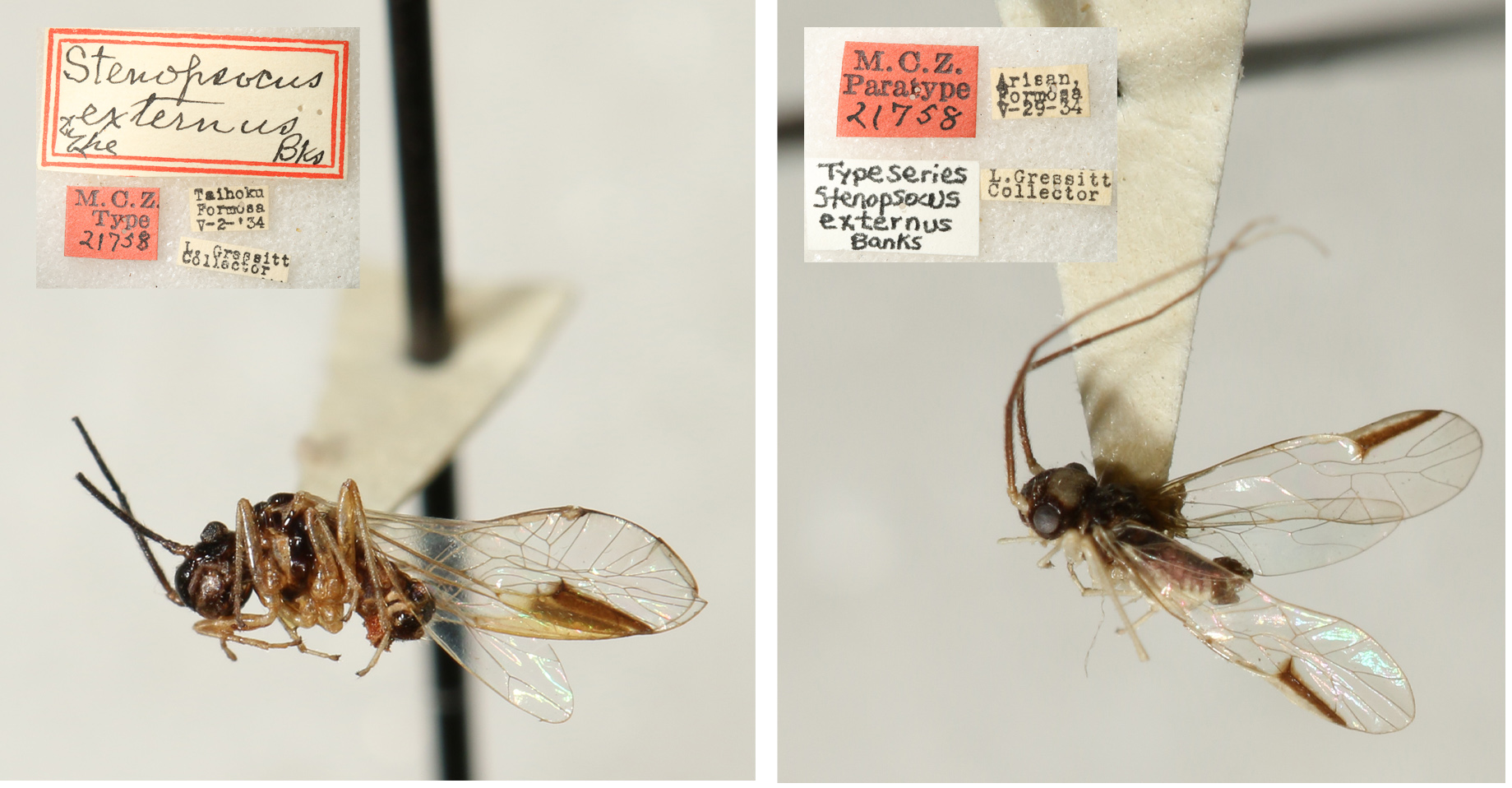

Supplement: Supplementary file 1 [file insects-16-01147-s001.zip › Figure S22 Stenopsocus externus.jpg]

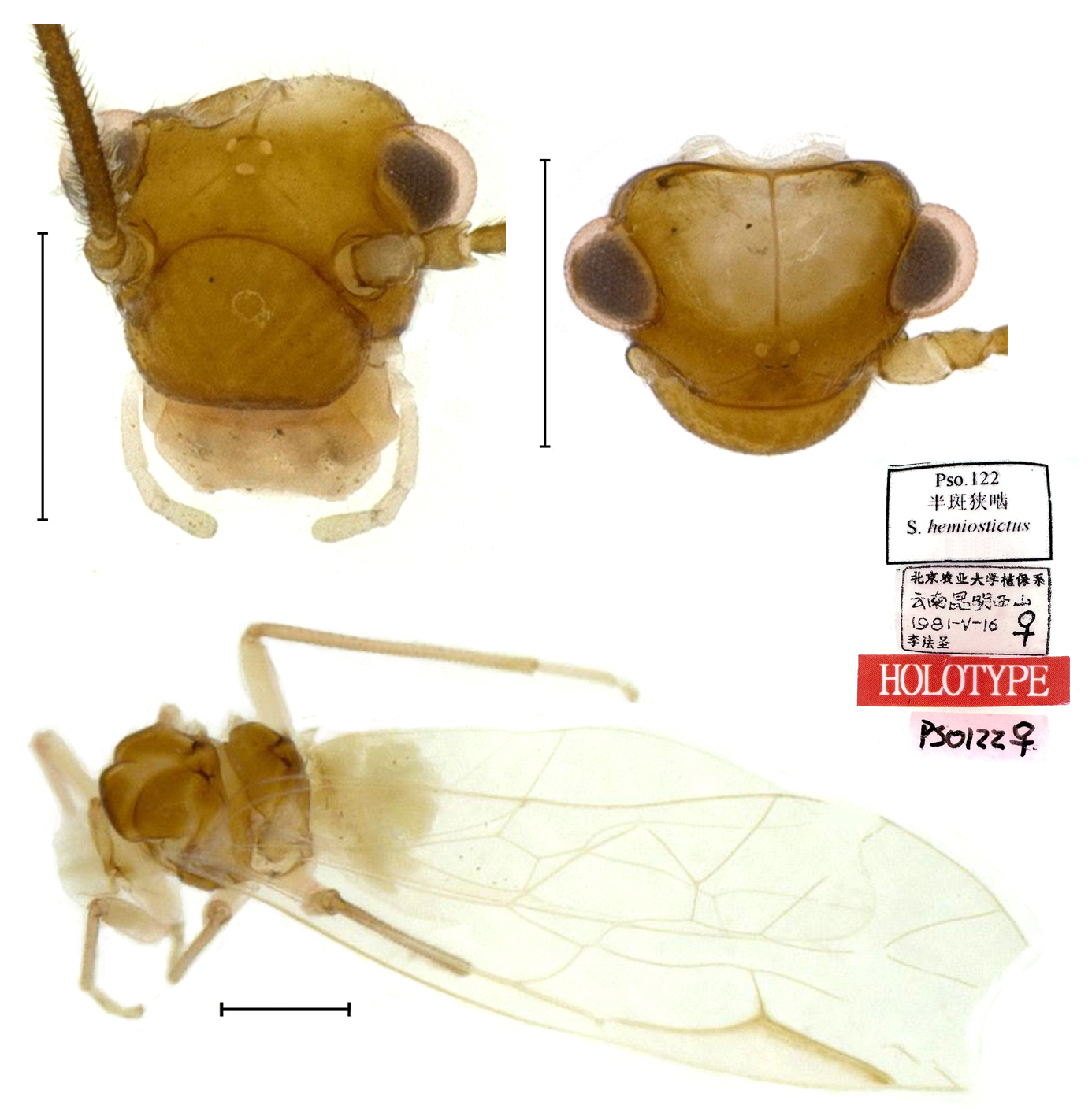

Supplement: Supplementary file 1 [file insects-16-01147-s001.zip › Figure S23 Holotype of Stenopsocus hemiostictus.jpg]

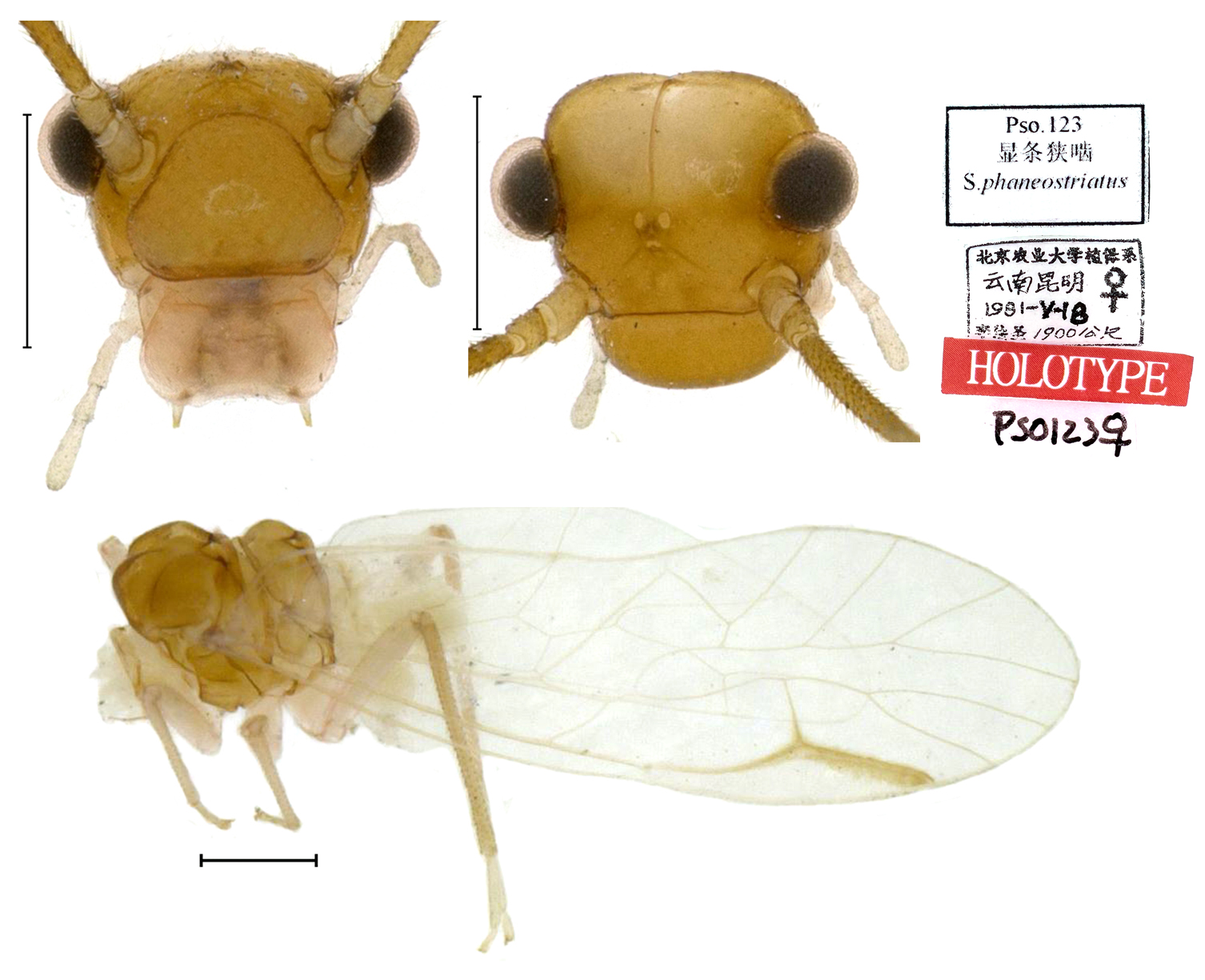

Supplement: Supplementary file 1 [file insects-16-01147-s001.zip › Figure S24 Holotype of Stenopsocus phaneostriatus .jpg]

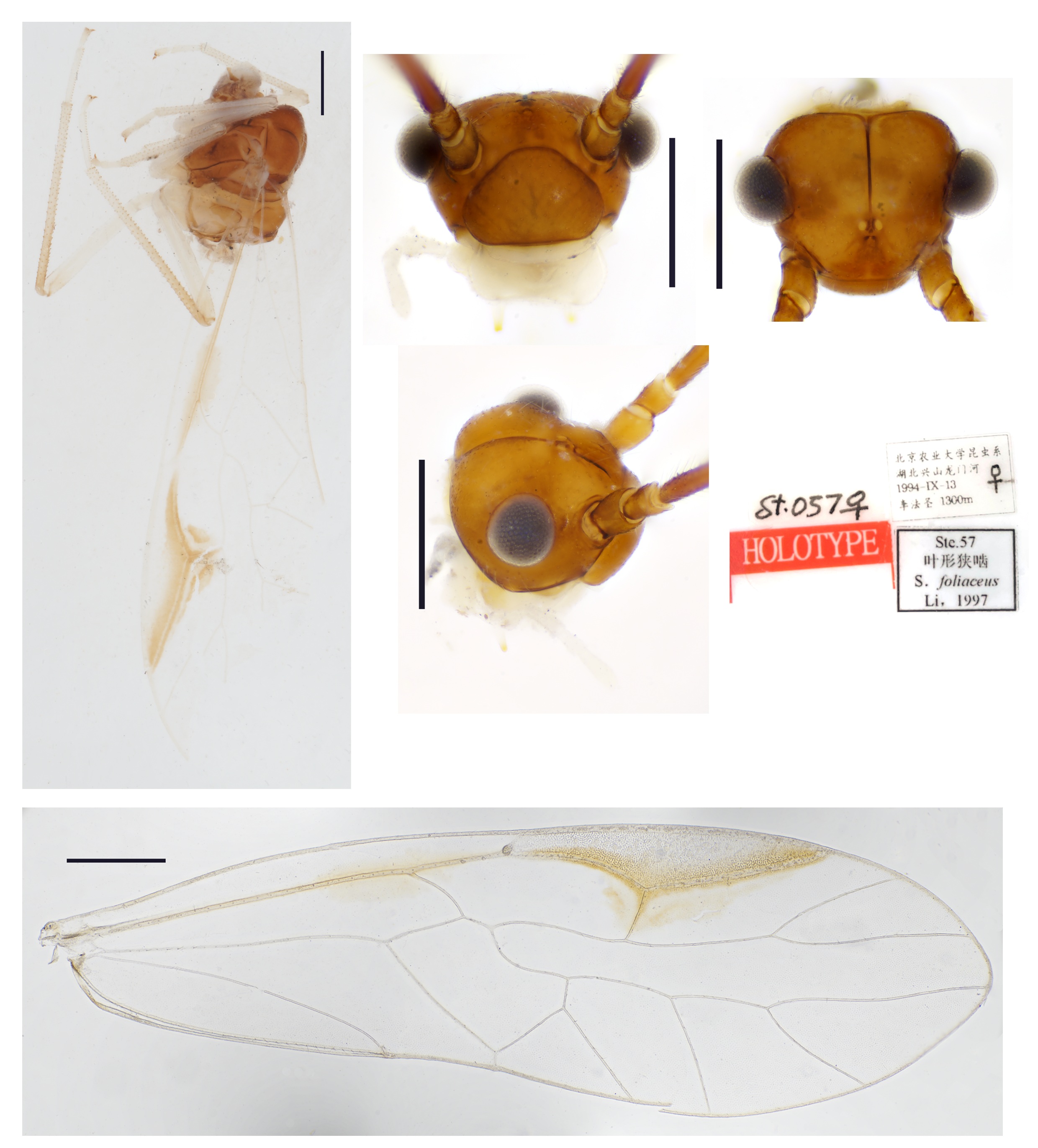

Supplement: Supplementary file 1 [file insects-16-01147-s001.zip › Figure S25 Holotype of Stenopsocus foliaceus.jpg]

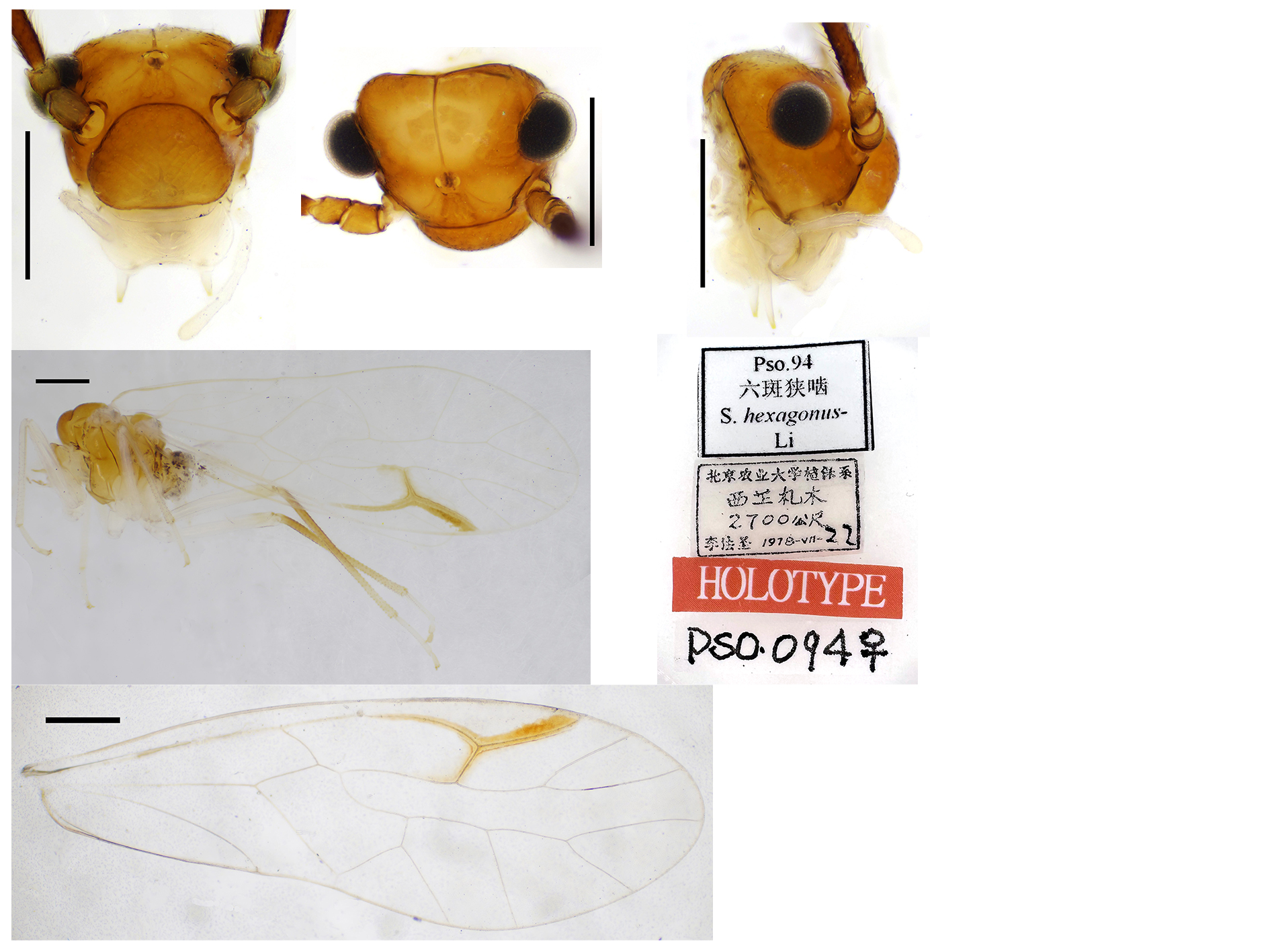

Supplement: Supplementary file 1 [file insects-16-01147-s001.zip › Figure S26 Holotype of Stenopsocus hexagonus.jpg]

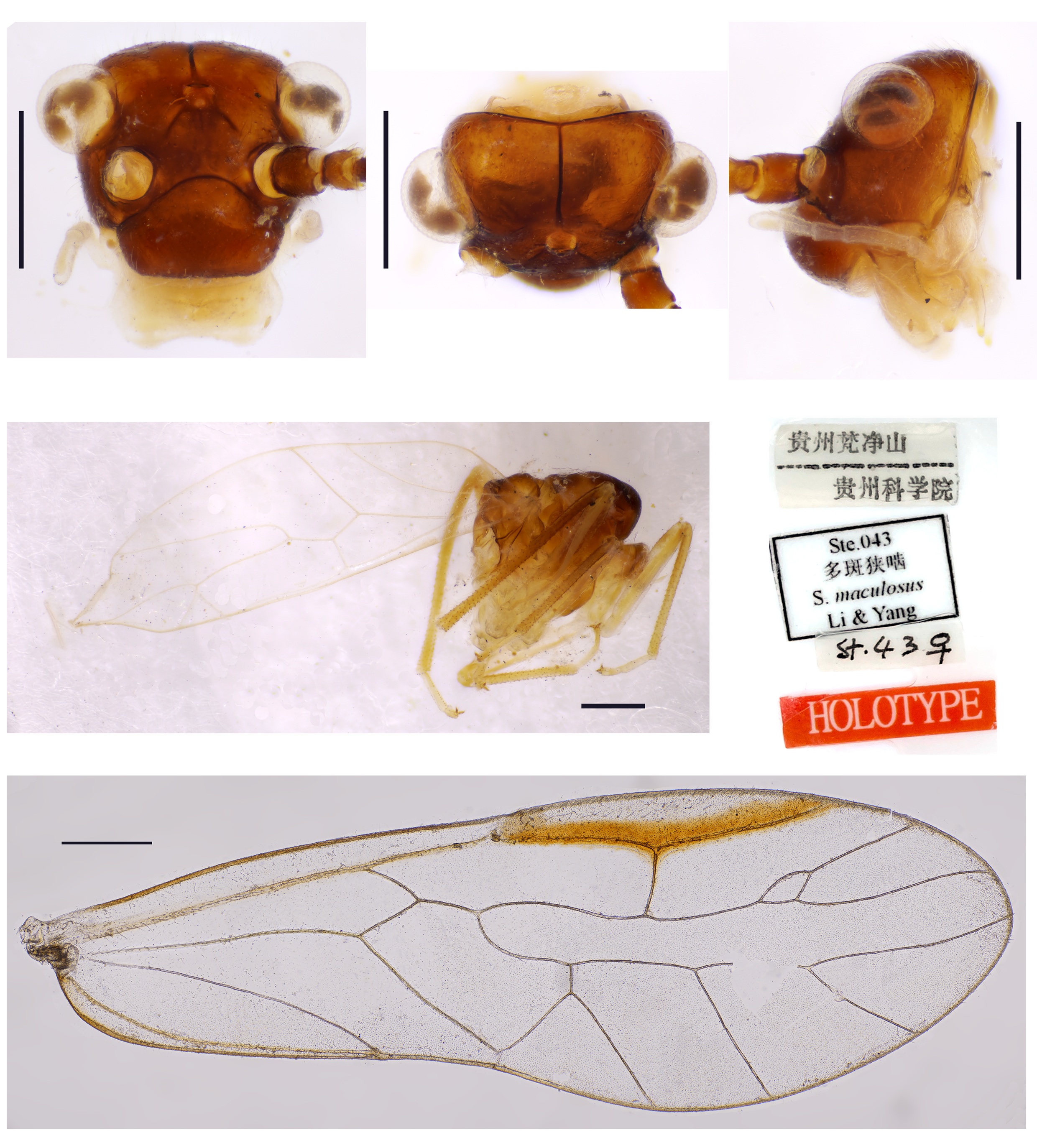

Supplement: Supplementary file 1 [file insects-16-01147-s001.zip › Figure S27 Holotype of Stenopsocus maculosus.jpg]

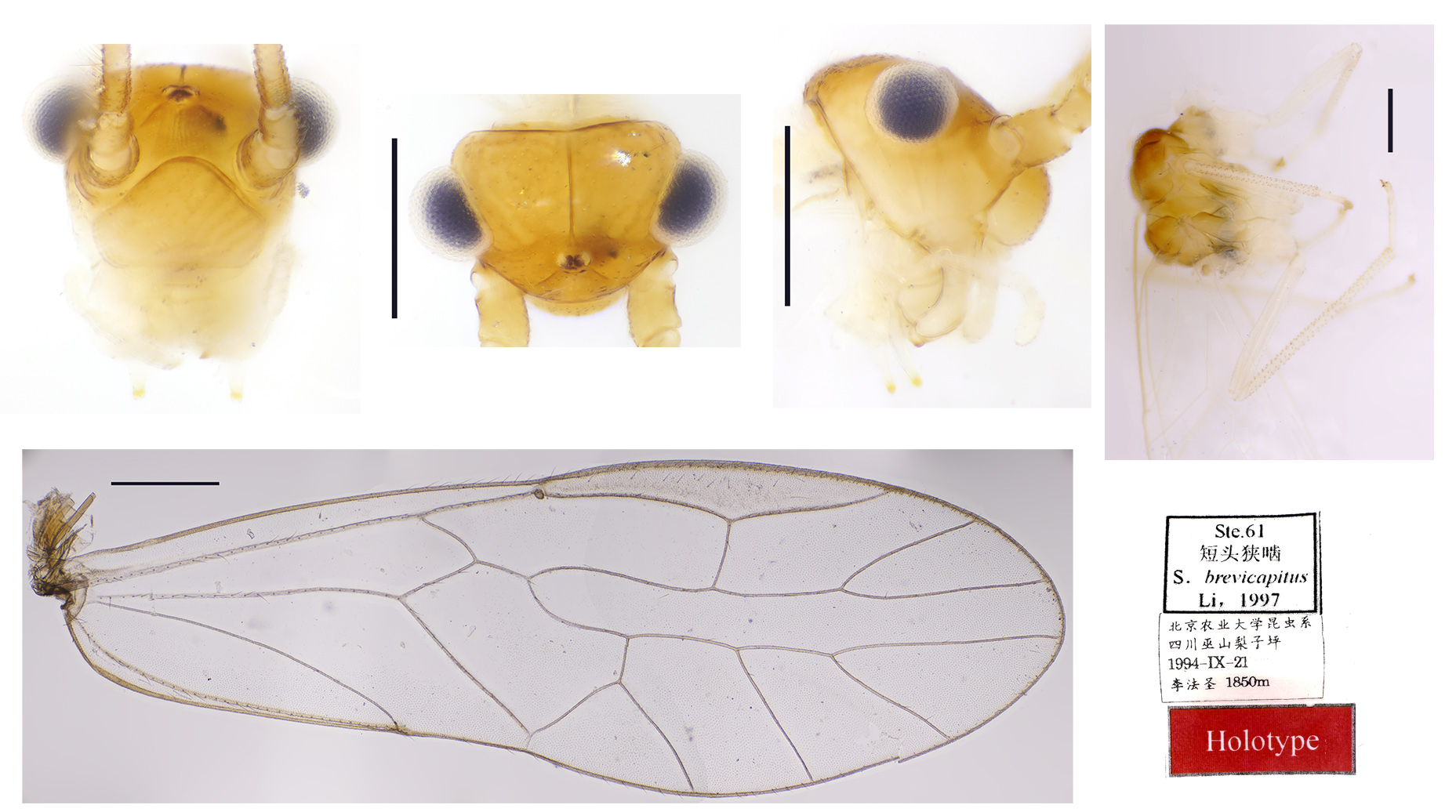

Supplement: Supplementary file 1 [file insects-16-01147-s001.zip › Figure S28 Holotype of Stenopsocus brevicapitus.jpg]

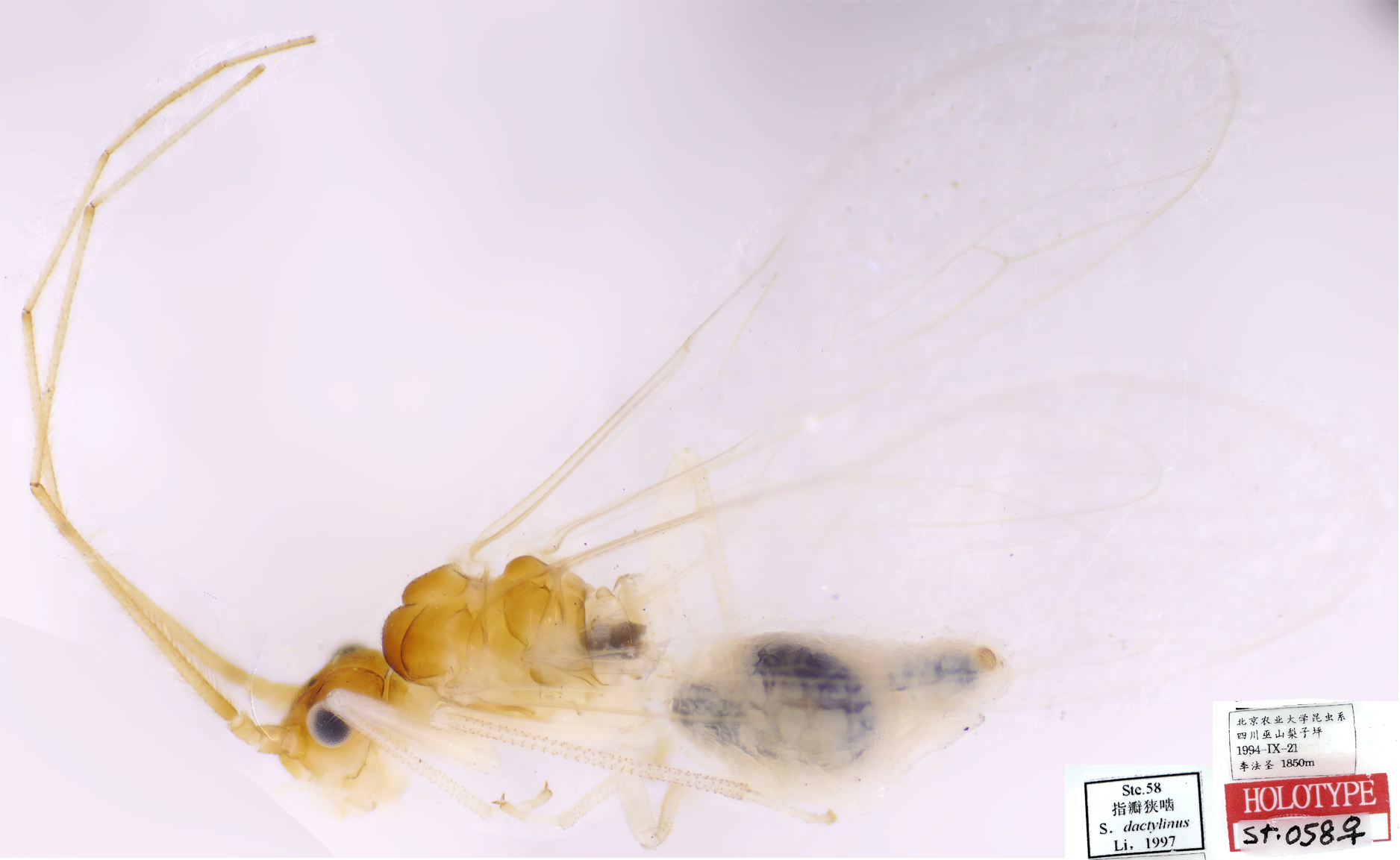

Supplement: Supplementary file 1 [file insects-16-01147-s001.zip › Figure S29 Holotype of Stenopsocus dactylinus.jpg]

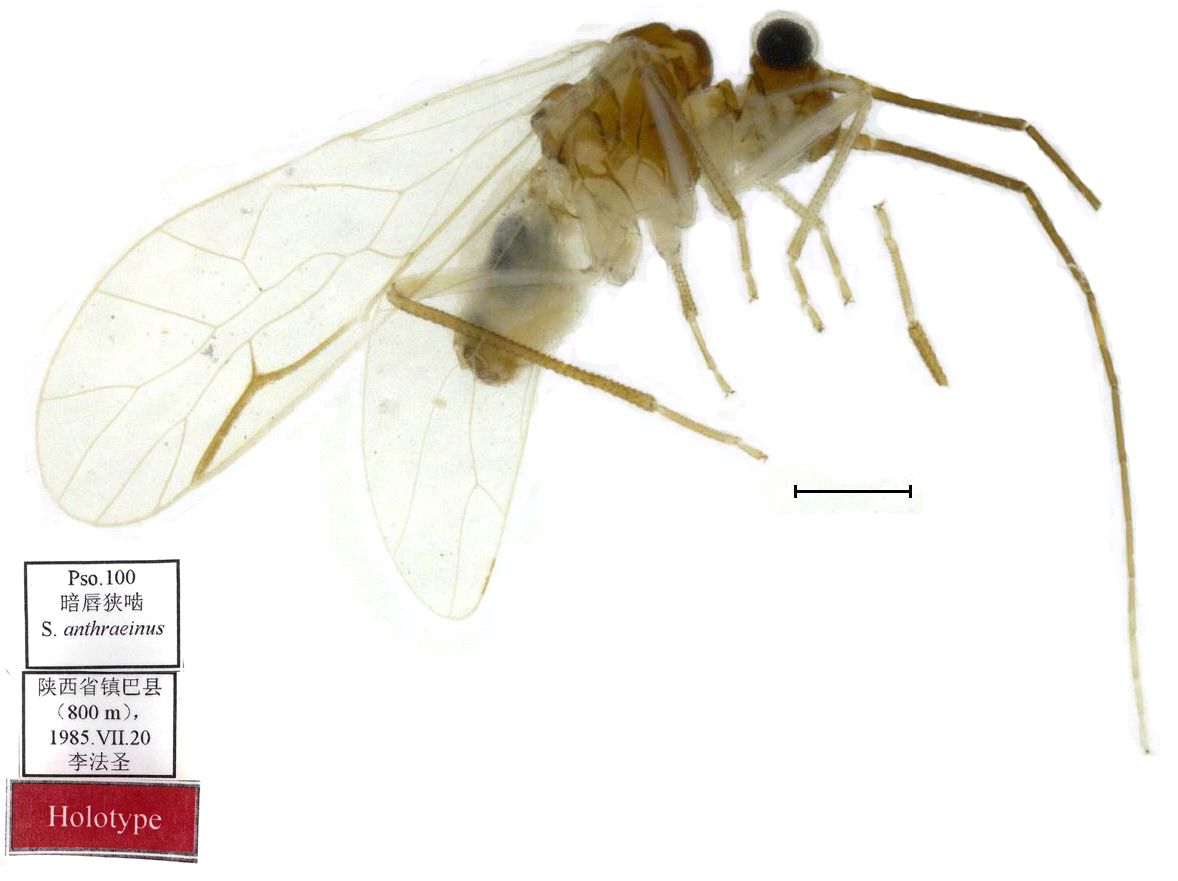

Supplement: Supplementary file 1 [file insects-16-01147-s001.zip › Figure S3 Holotype of Stenopsocus anthracinus.jpg]

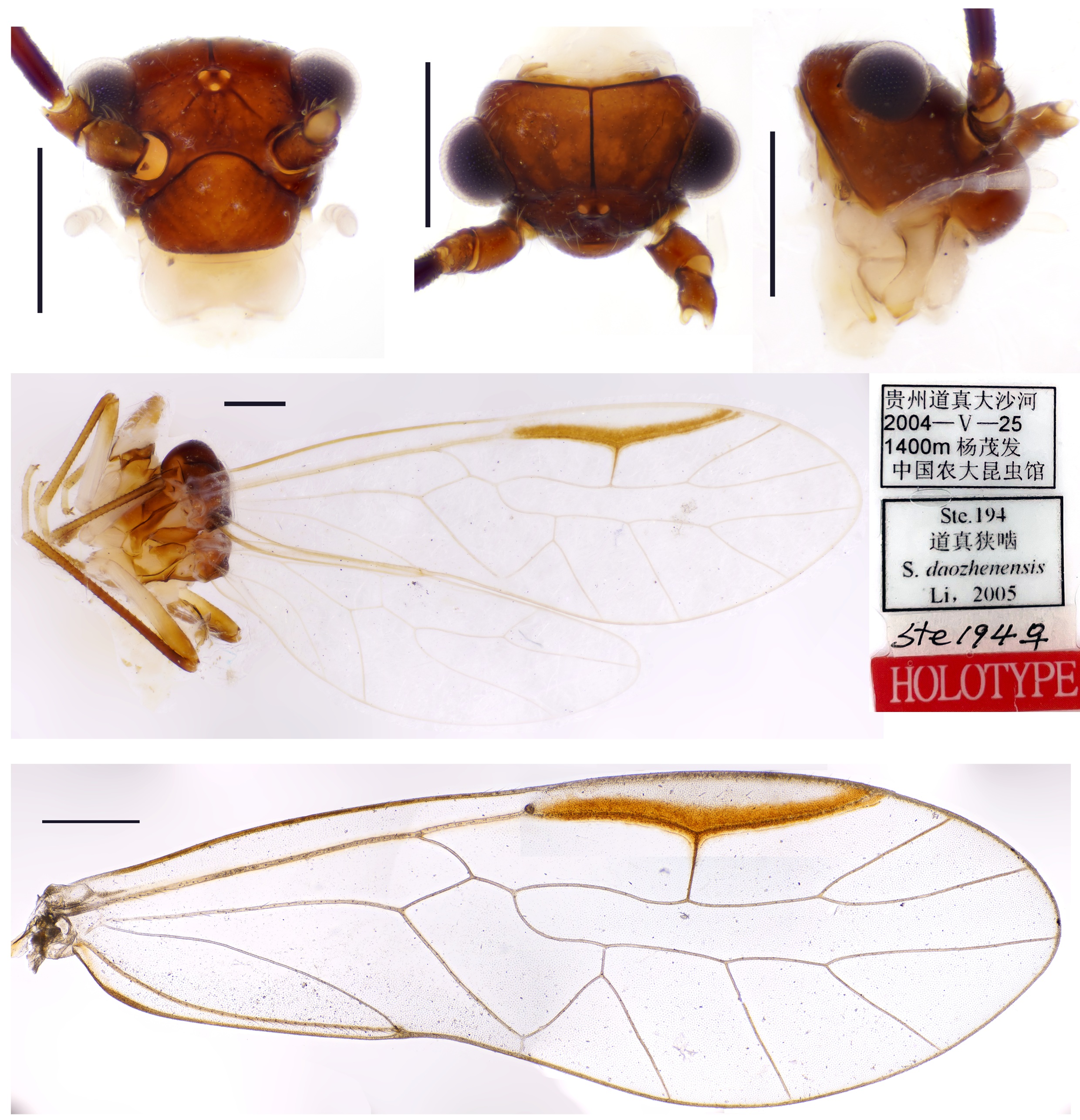

Supplement: Supplementary file 1 [file insects-16-01147-s001.zip › Figure S30 Holotype of Stenopsocus daozheniensis.jpg]

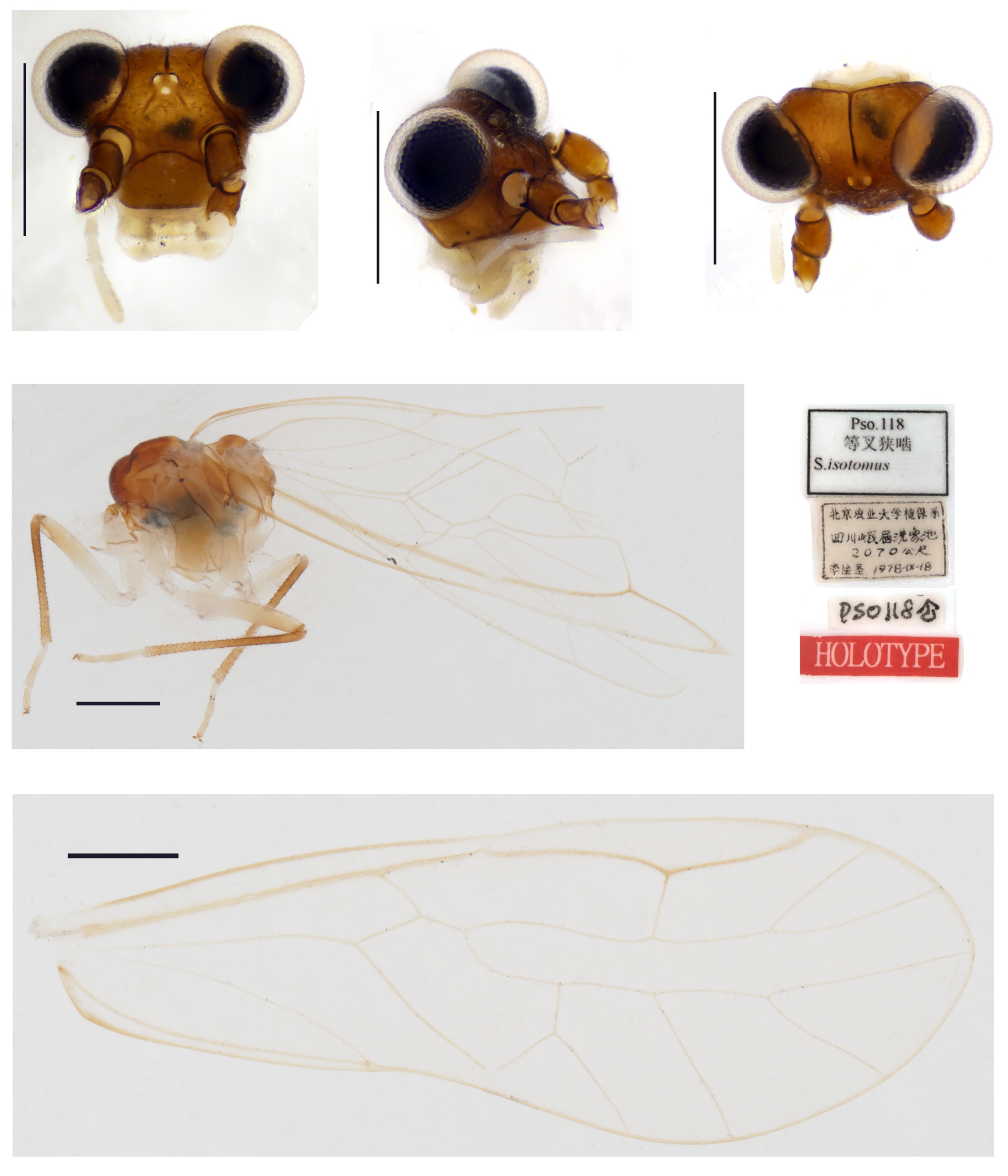

Supplement: Supplementary file 1 [file insects-16-01147-s001.zip › Figure S31 Holotype of Stenopsocus isotomus.jpg]

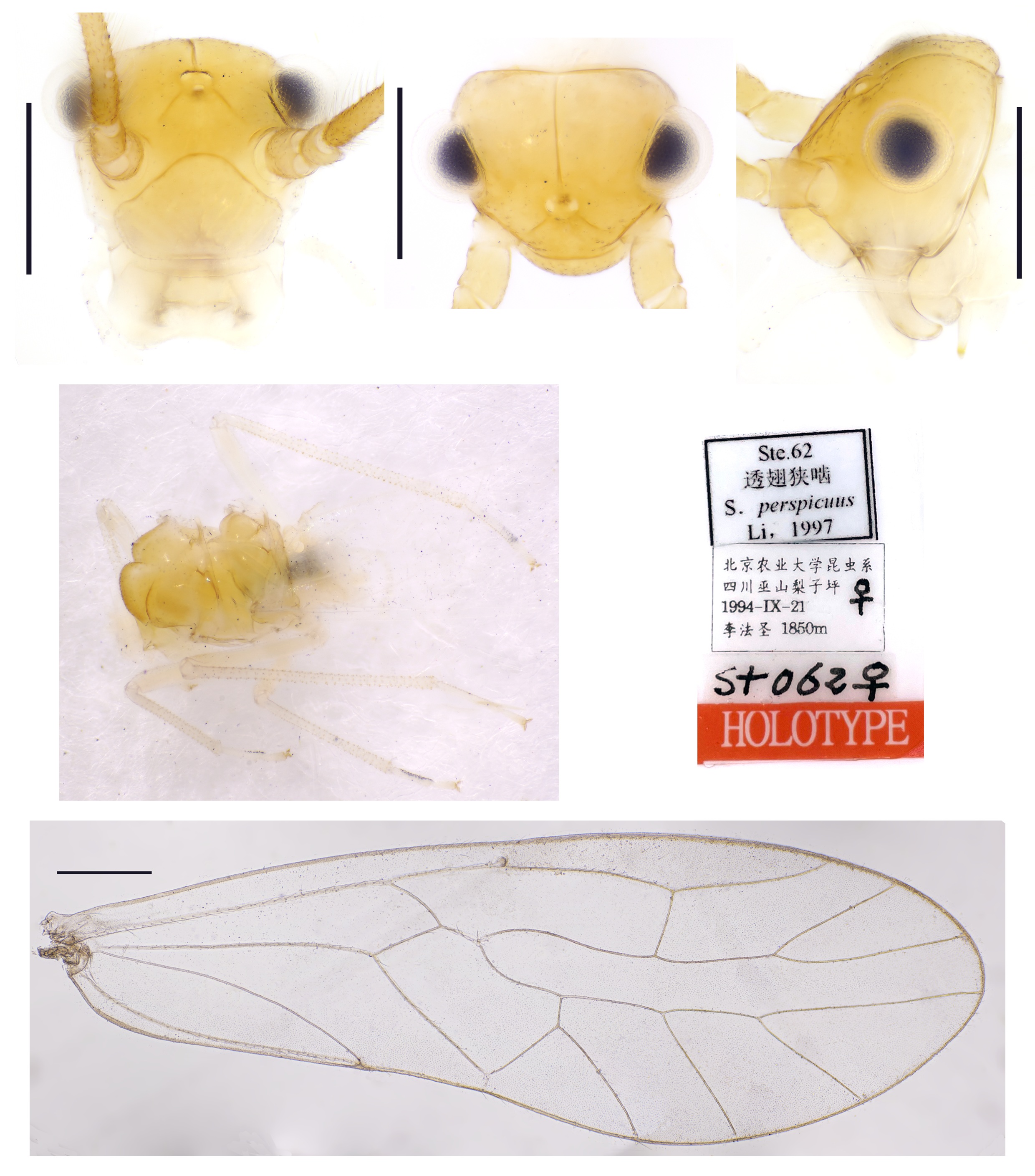

Supplement: Supplementary file 1 [file insects-16-01147-s001.zip › Figure S32 Holotype of Stenopsocus perspicuus.jpg]

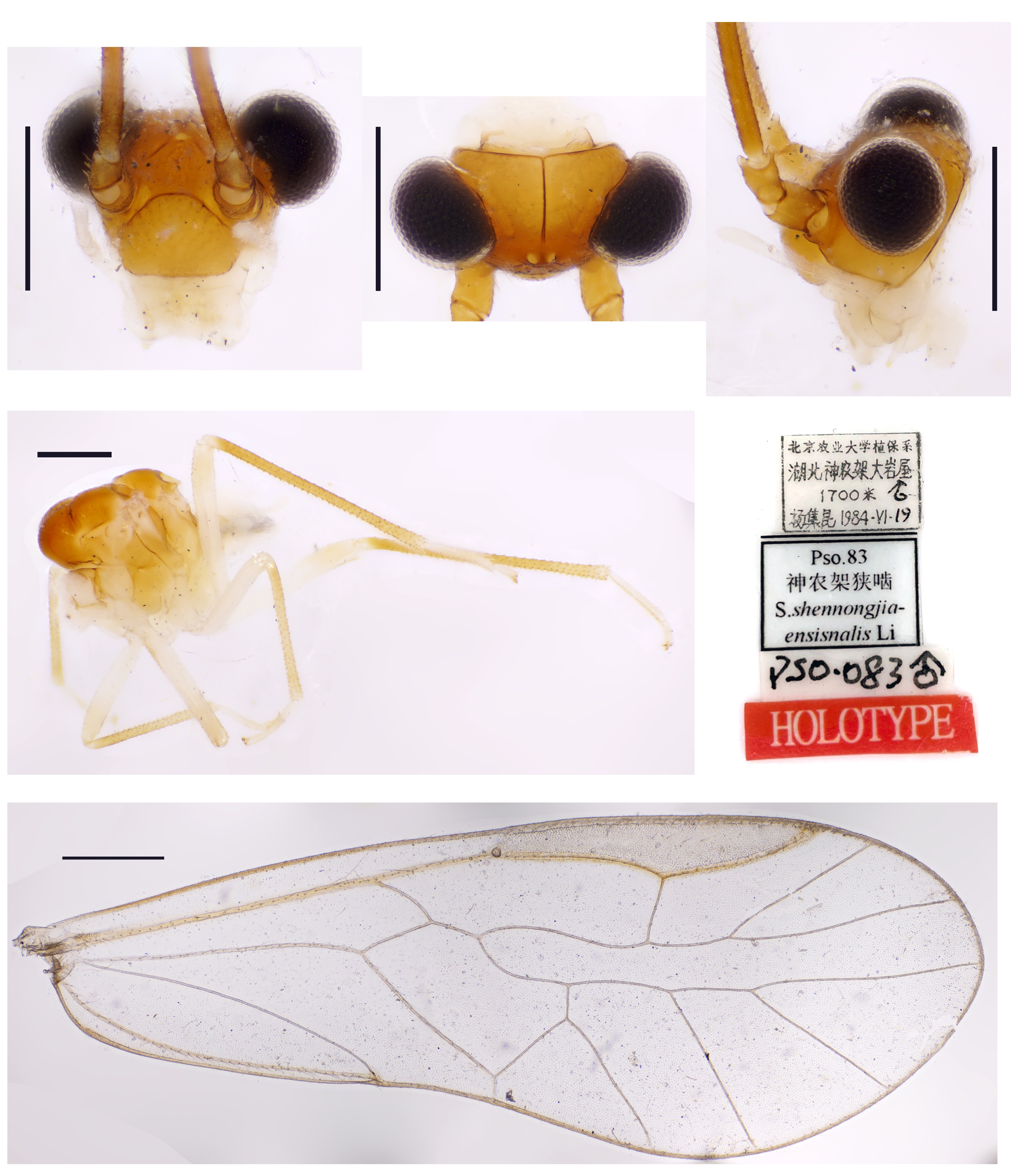

Supplement: Supplementary file 1 [file insects-16-01147-s001.zip › Figure S33 Holotype of Stenopsocus shennongjiaensis.jpg]

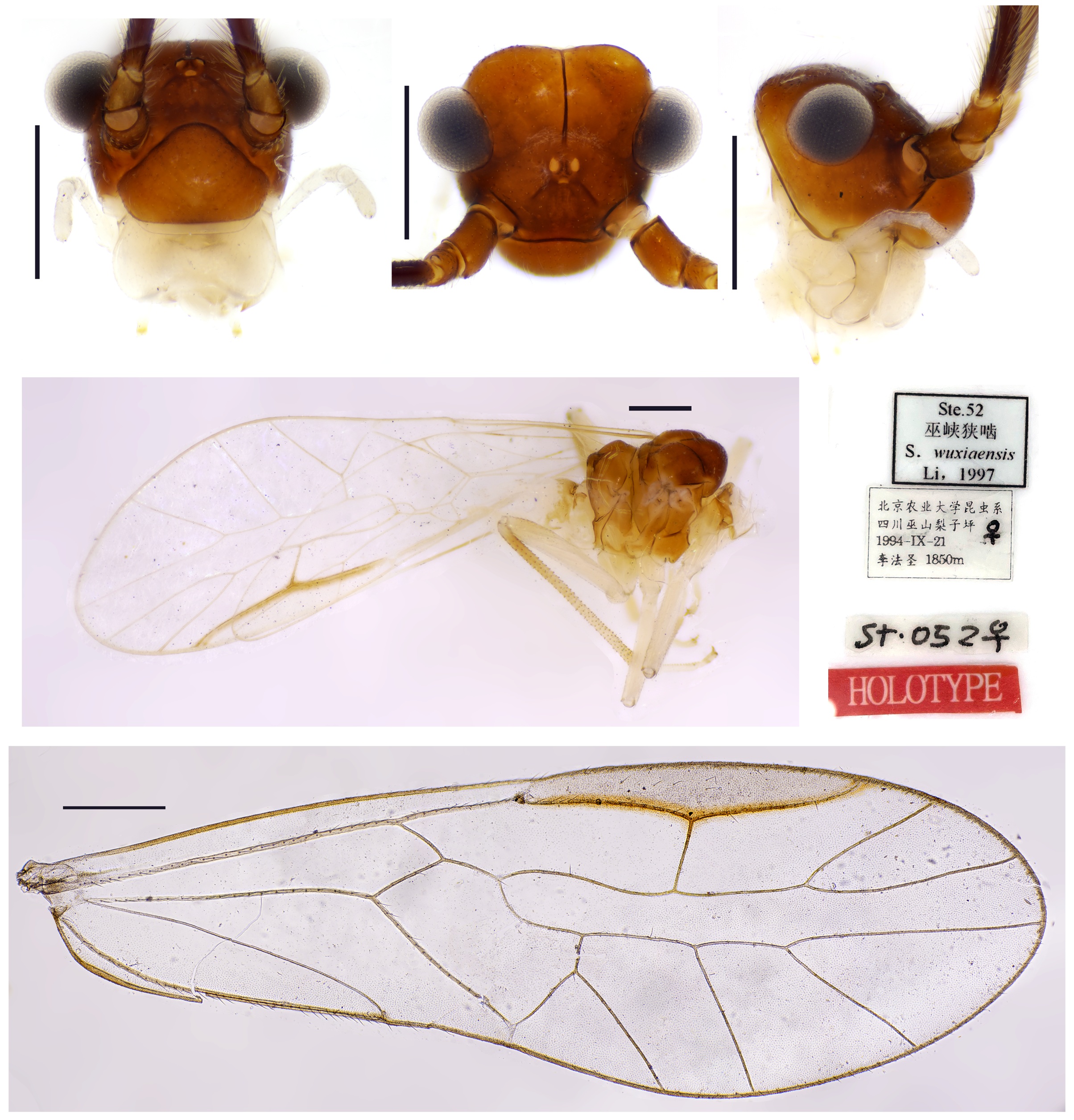

Supplement: Supplementary file 1 [file insects-16-01147-s001.zip › Figure S34 Holotype of Stenopsocus wuxiaensis.jpg]

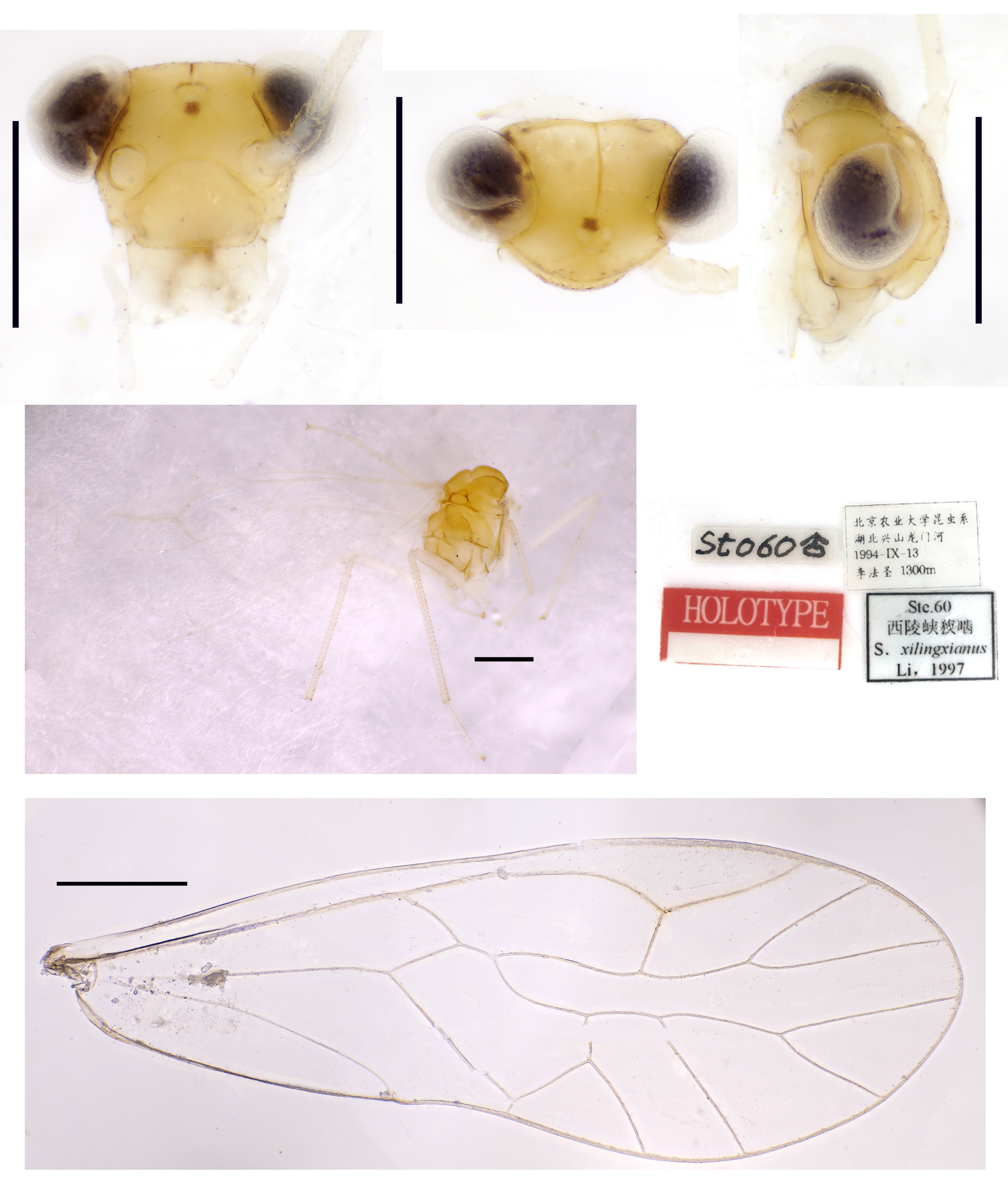

Supplement: Supplementary file 1 [file insects-16-01147-s001.zip › Figure S35 Holotype of Stenopsocus xilingxianicus.jpg]

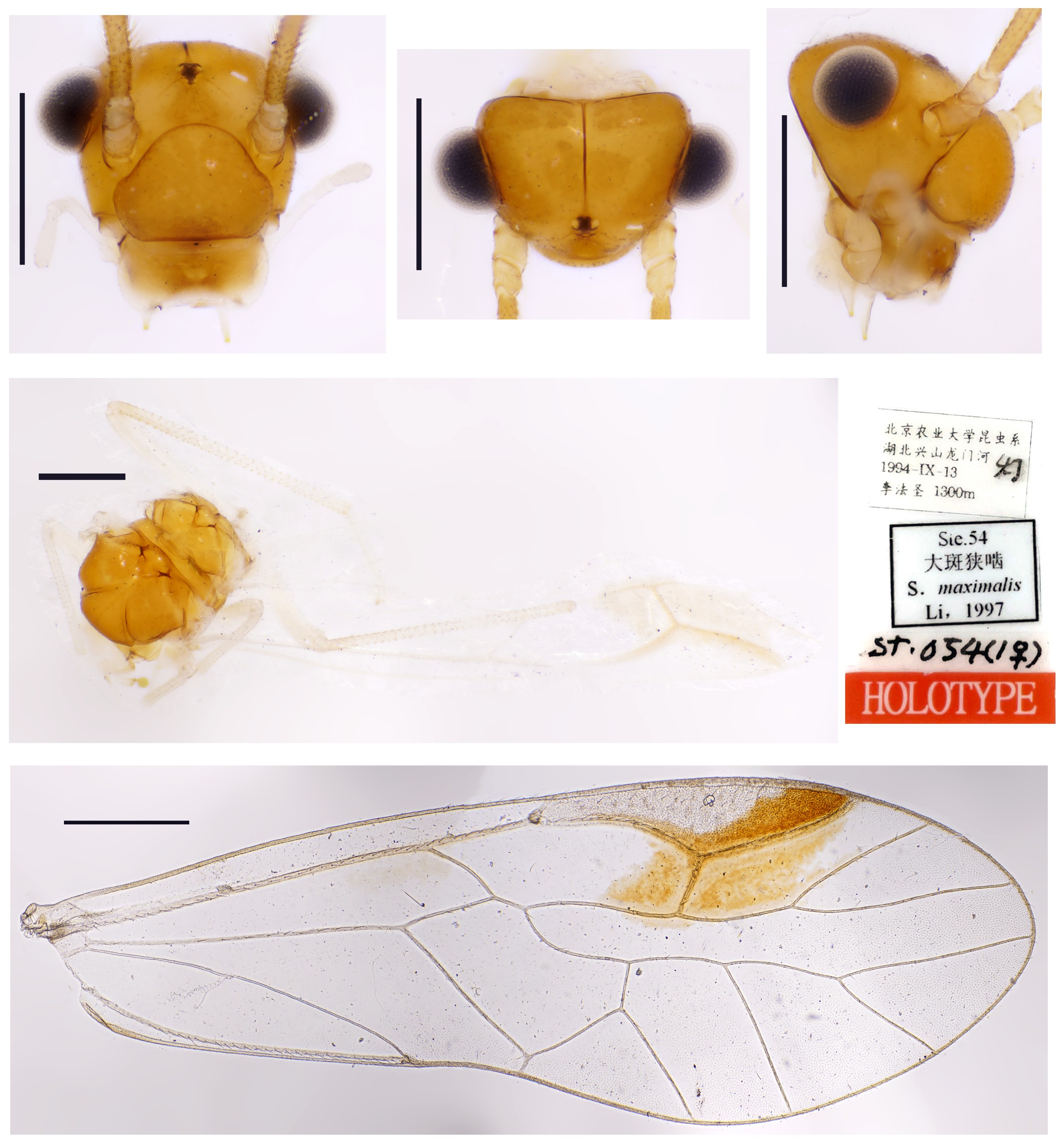

Supplement: Supplementary file 1 [file insects-16-01147-s001.zip › Figure S36 Holotype of Stenopsocus maximalis.jpeg]

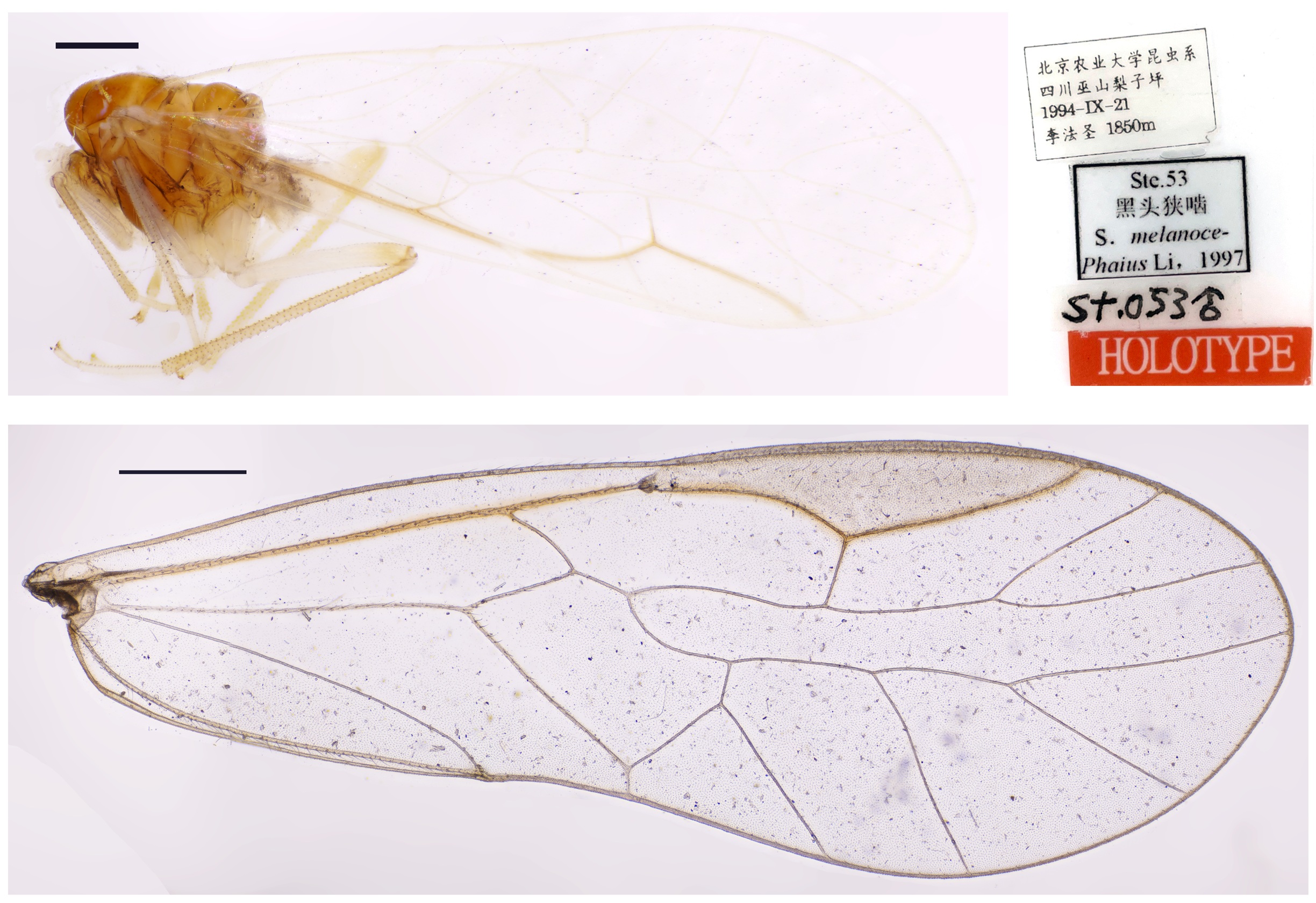

Supplement: Supplementary file 1 [file insects-16-01147-s001.zip › Figure S37 Holotype of Stenopsocus melanocephalus.jpg]

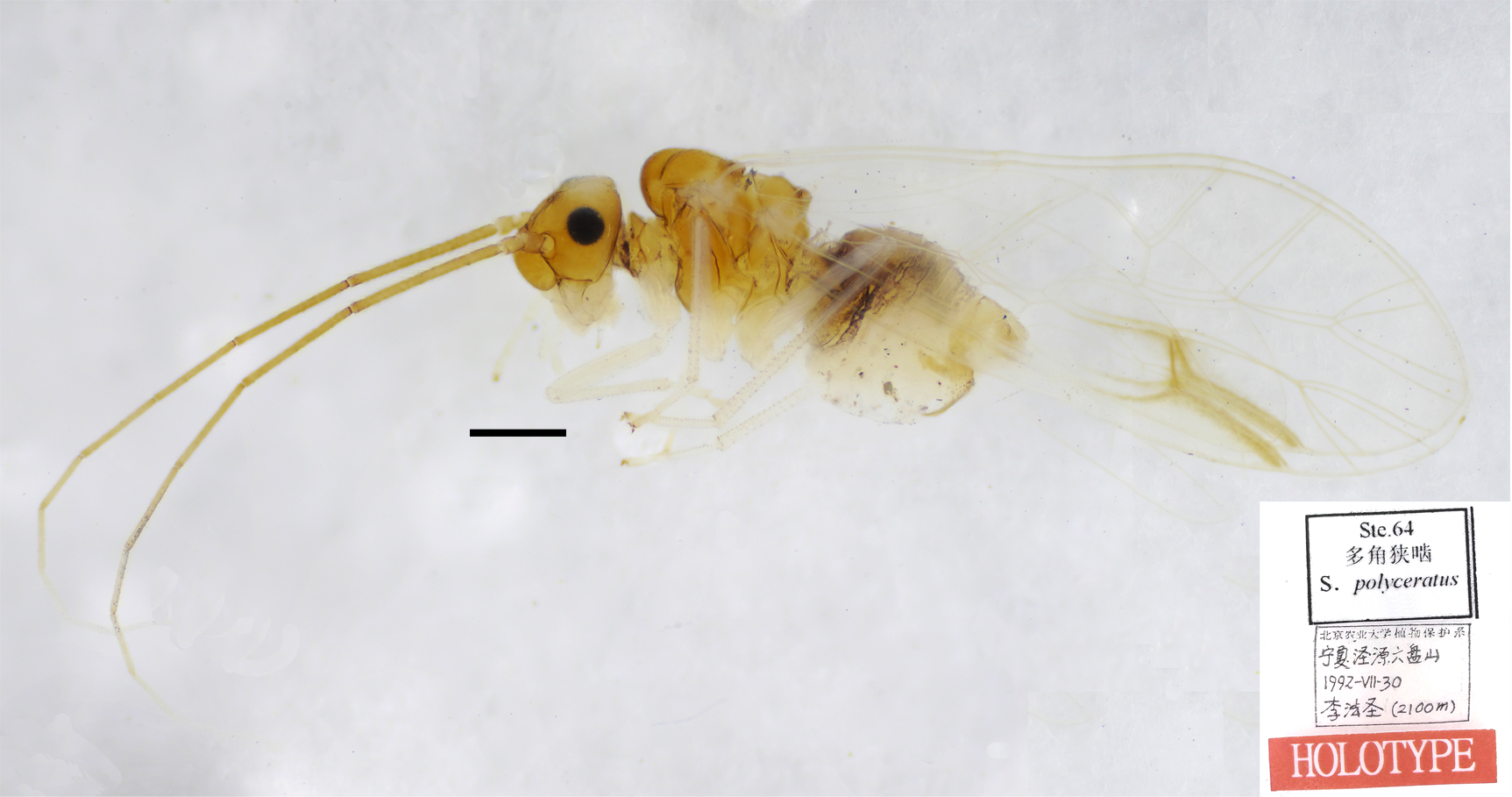

Supplement: Supplementary file 1 [file insects-16-01147-s001.zip › Figure S38 Holotype of Stenopsocus polyceratus.jpg]

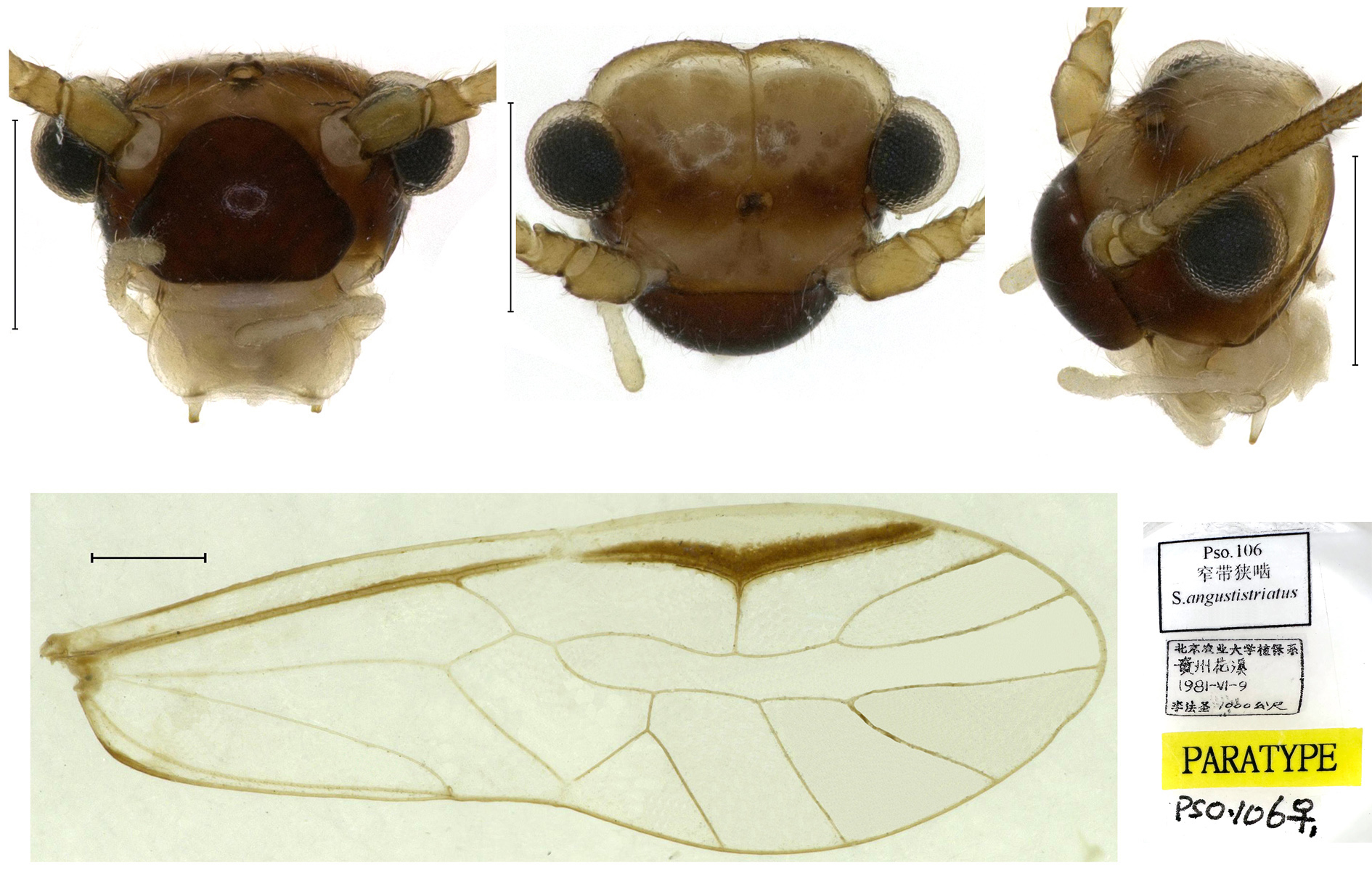

Supplement: Supplementary file 1 [file insects-16-01147-s001.zip › Figure S39 Paratype of Stenopsocus angustistriatus .jpg]

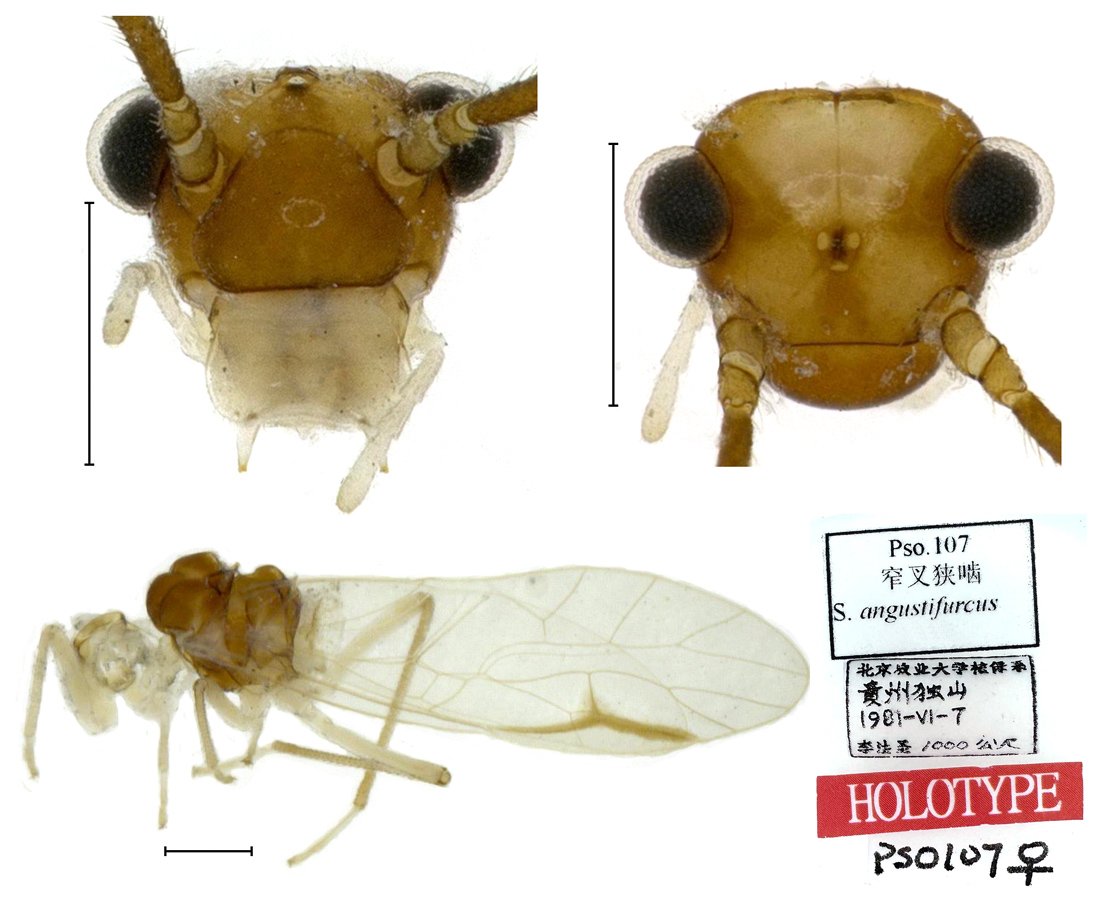

Supplement: Supplementary file 1 [file insects-16-01147-s001.zip › Figure S4 Holotype of Stenopsocus angustifurcus.jpg]

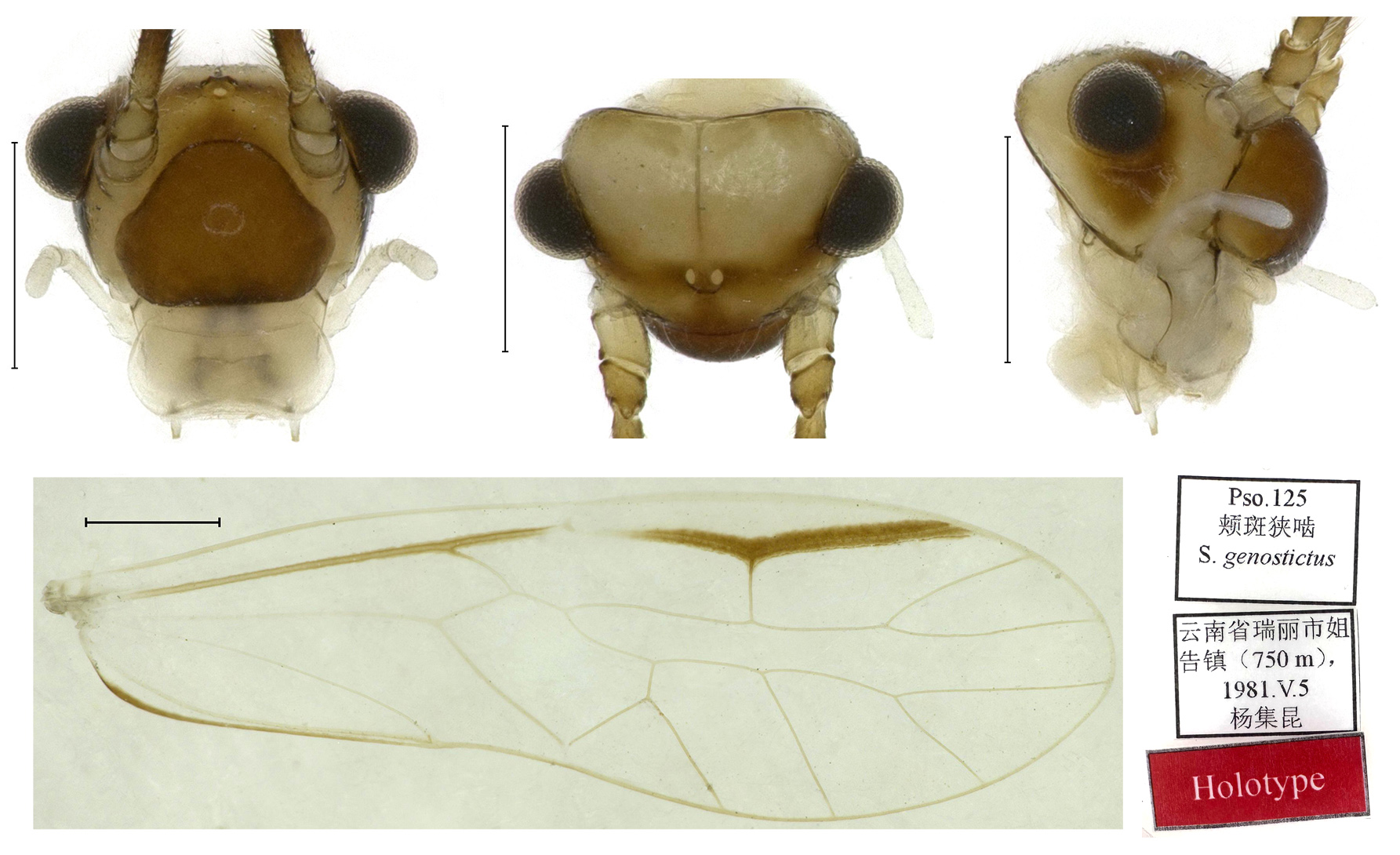

Supplement: Supplementary file 1 [file insects-16-01147-s001.zip › Figure S40 Holotype of Stenopsocus genostictus.jpg]

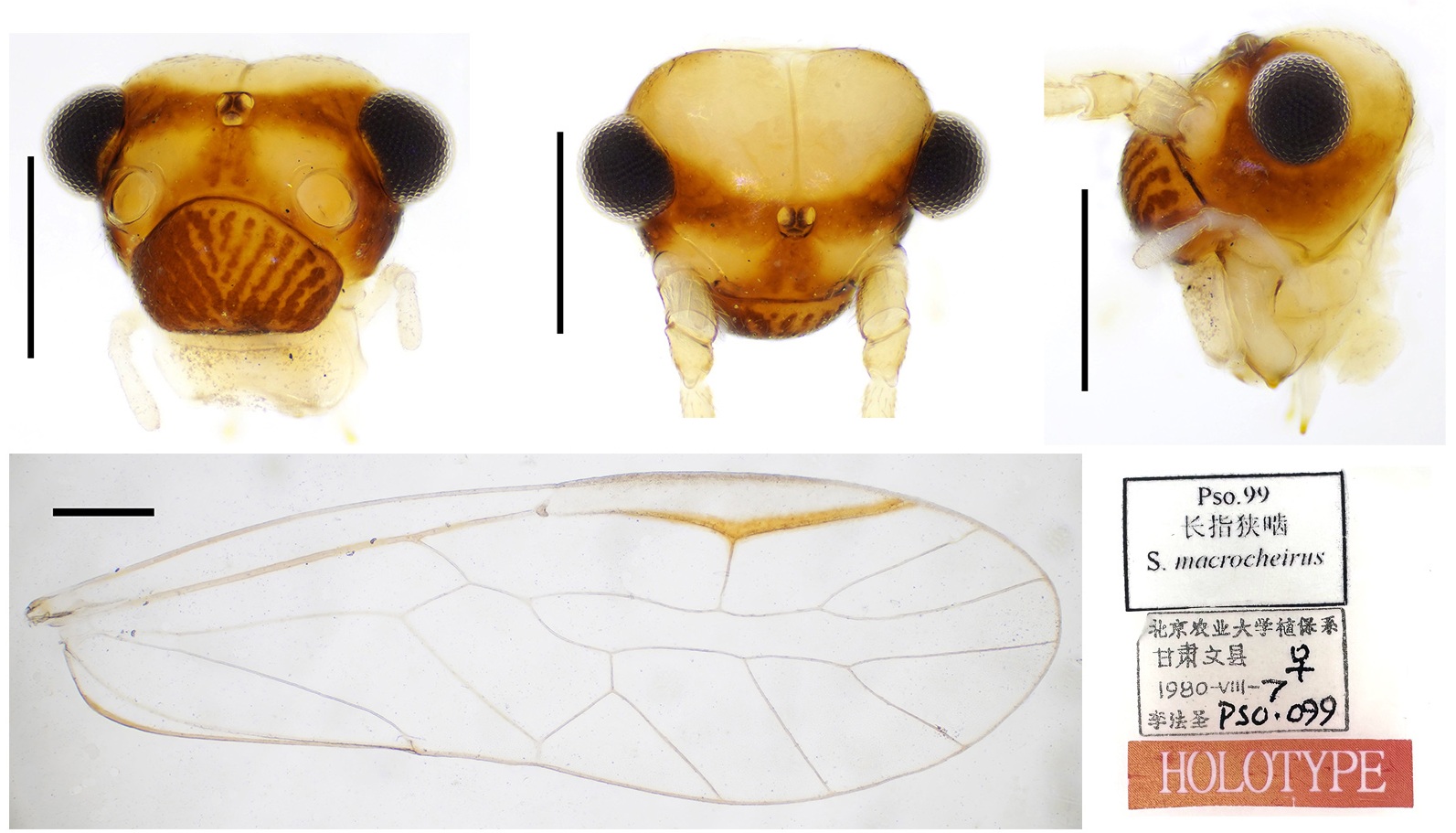

Supplement: Supplementary file 1 [file insects-16-01147-s001.zip › Figure S41 Holotype of Stenopsocus macrocheirus .jpg]

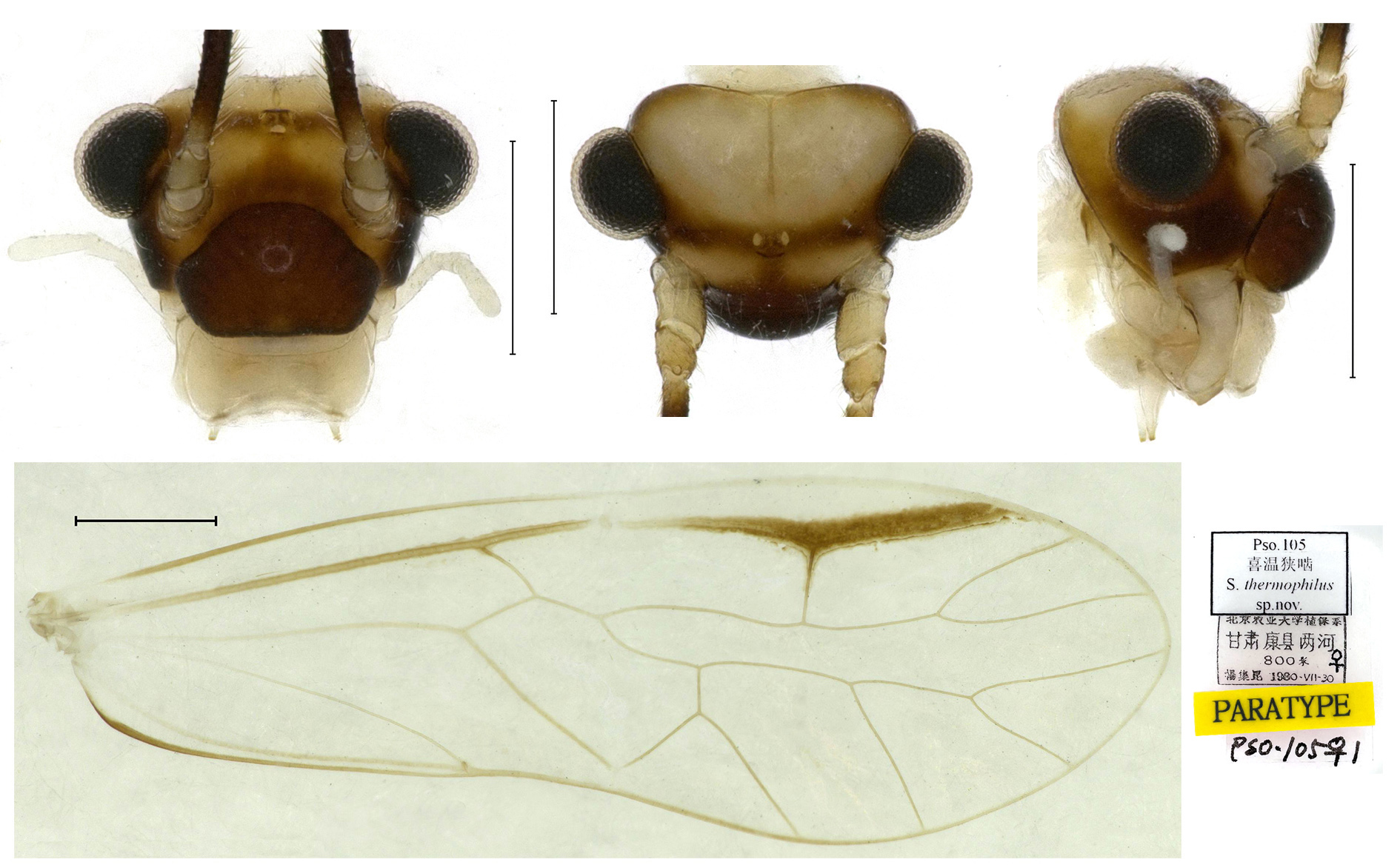

Supplement: Supplementary file 1 [file insects-16-01147-s001.zip › Figure S42 Paratype of Stenopsocus thermophile.jpg]

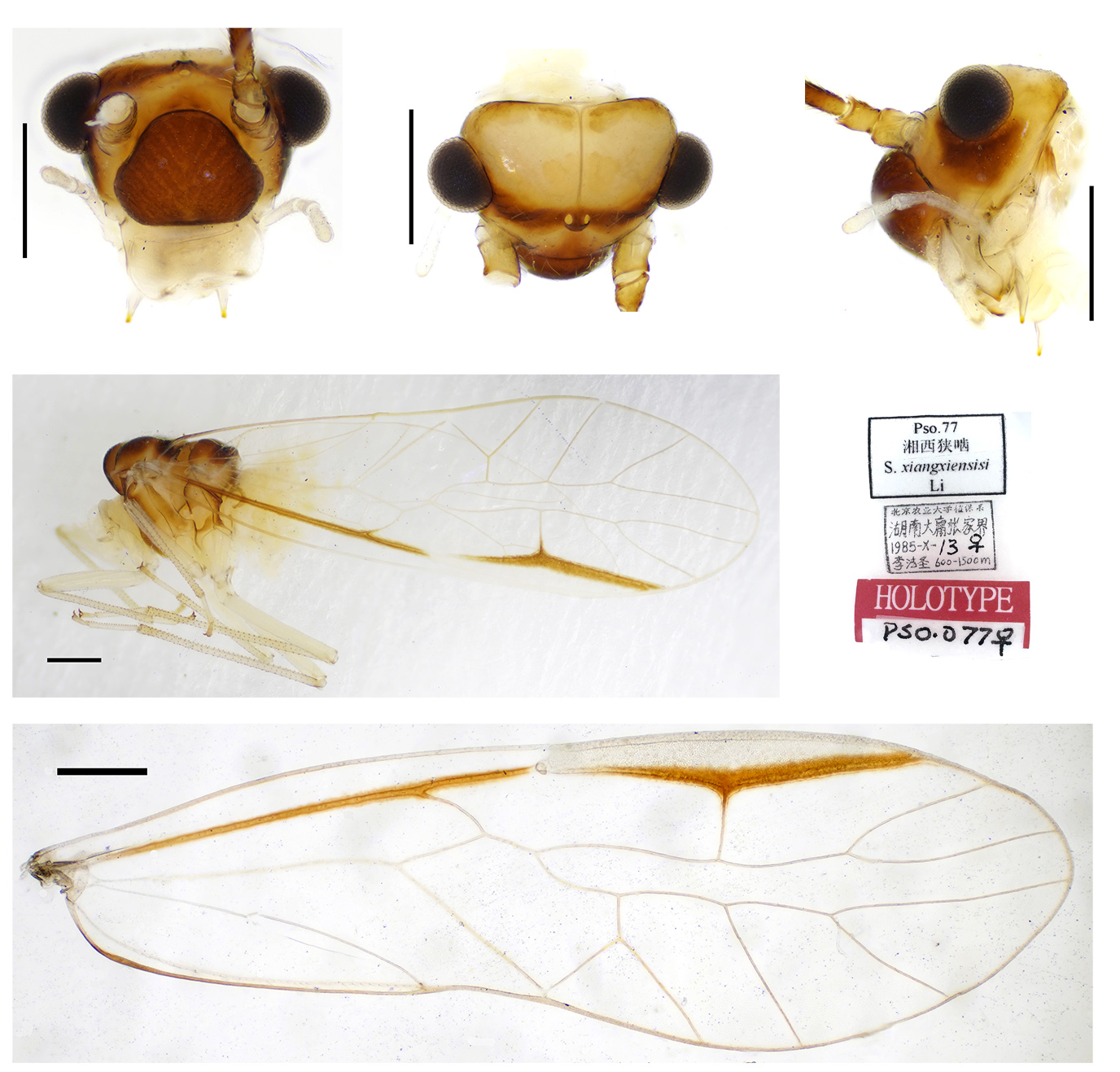

Supplement: Supplementary file 1 [file insects-16-01147-s001.zip › Figure S43 Holotype of Stenopsocus xiangxiensis .jpg]

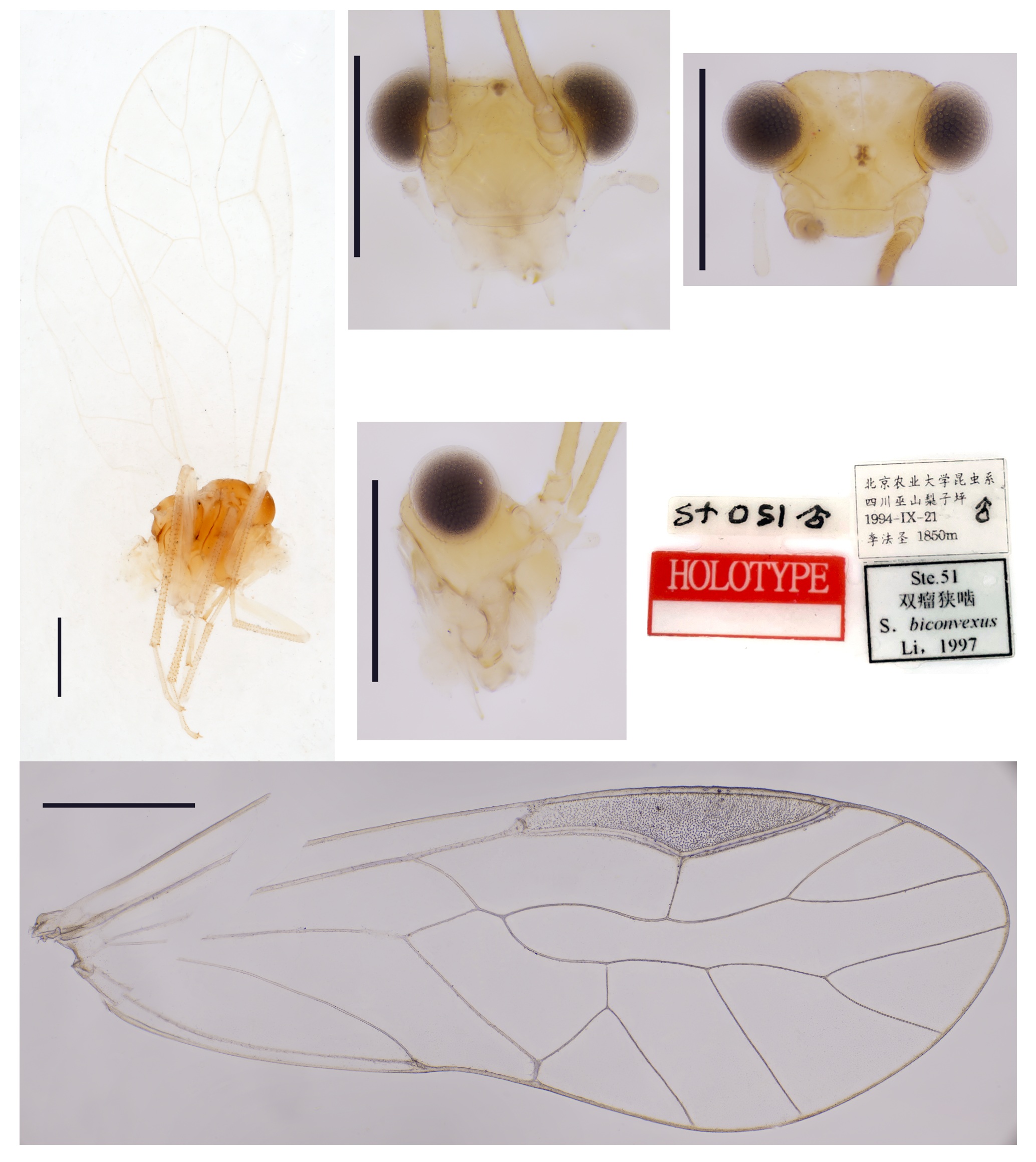

Supplement: Supplementary file 1 [file insects-16-01147-s001.zip › Figure S5 Holotype of Stenopsocus biconvexus .jpg]

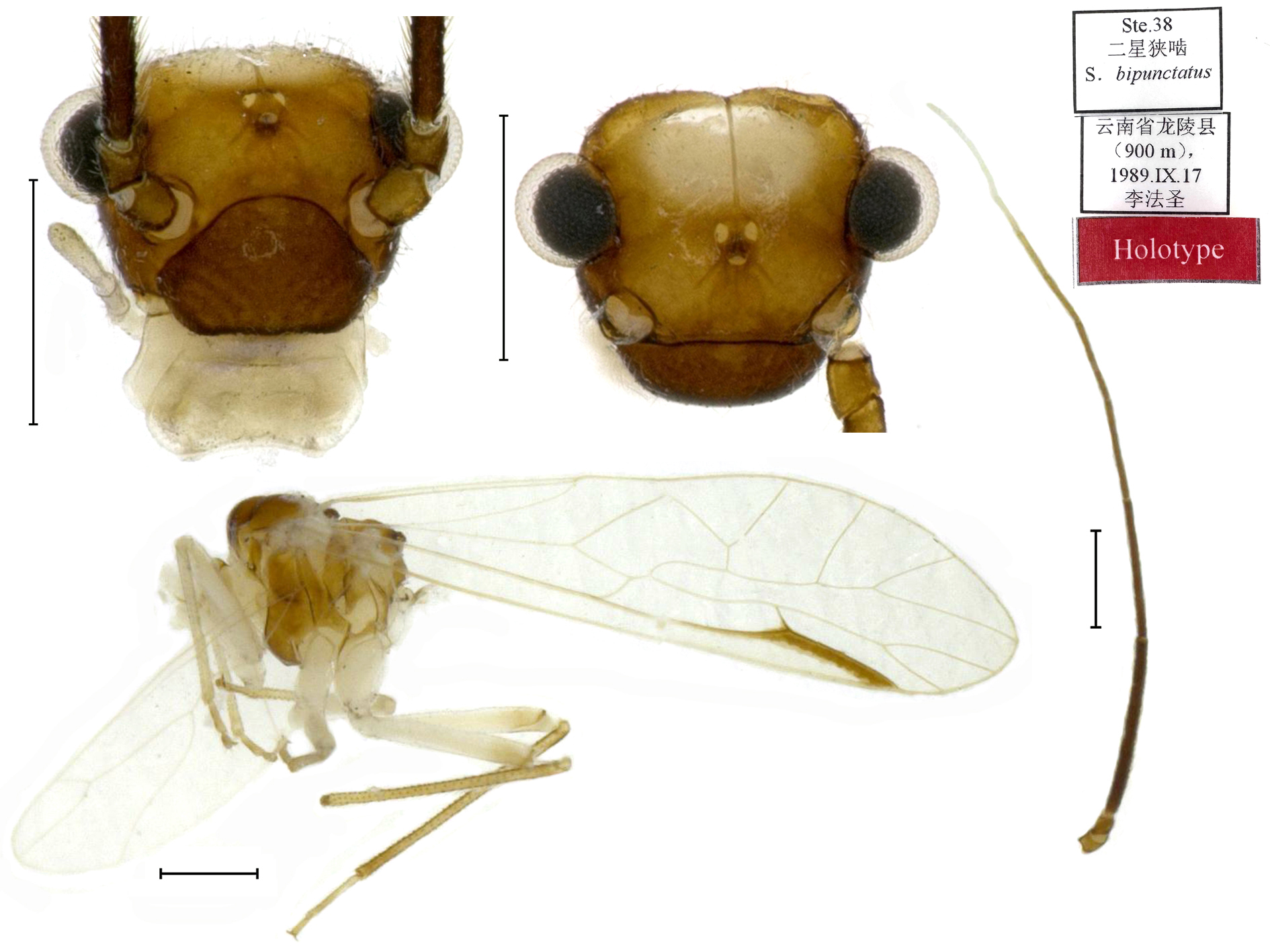

Supplement: Supplementary file 1 [file insects-16-01147-s001.zip › Figure S6 Holotype of Stenopsocus bipunctatus.jpg]

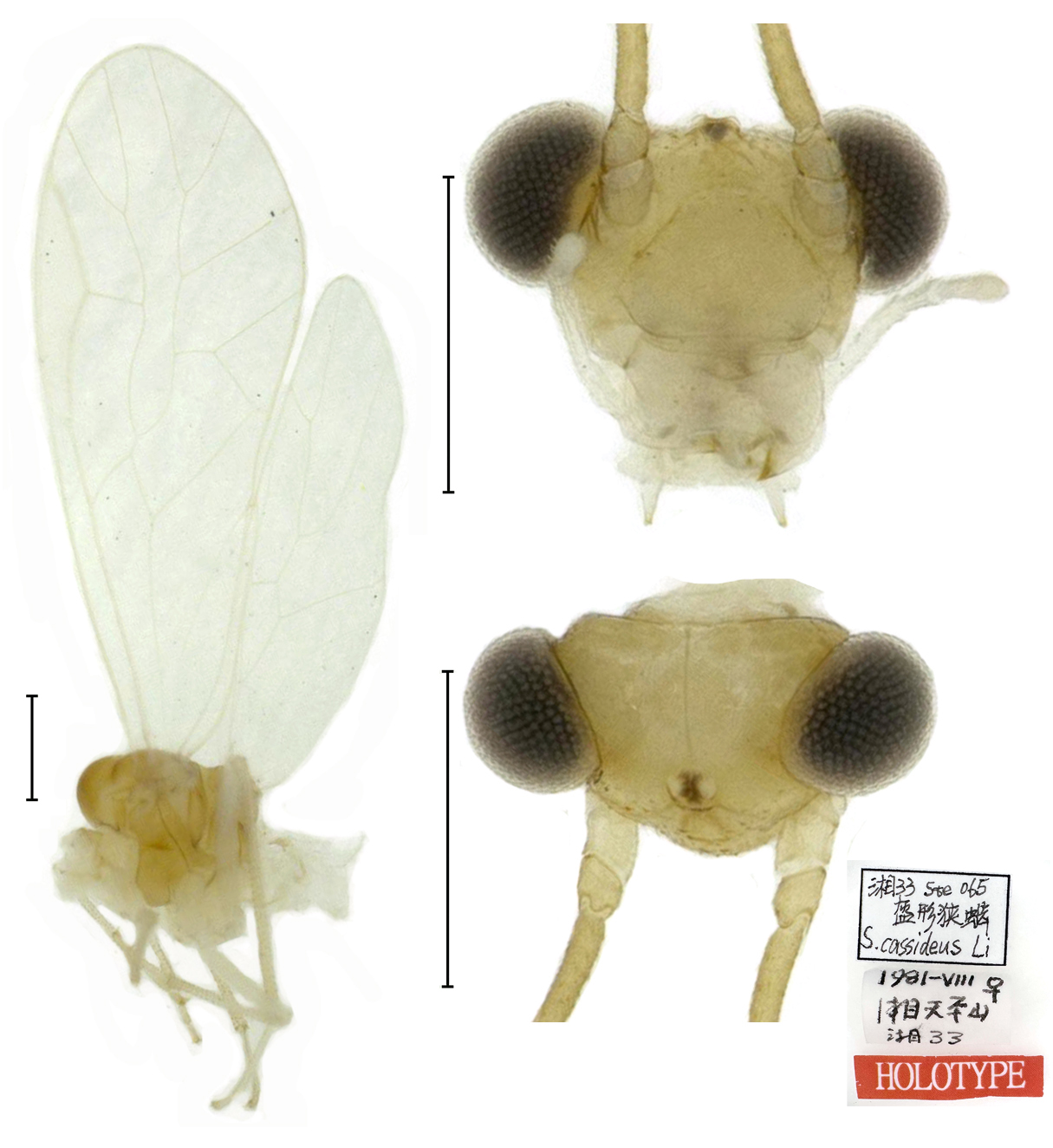

Supplement: Supplementary file 1 [file insects-16-01147-s001.zip › Figure S7 Holotype of Stenopsocus cassideus.jpg]

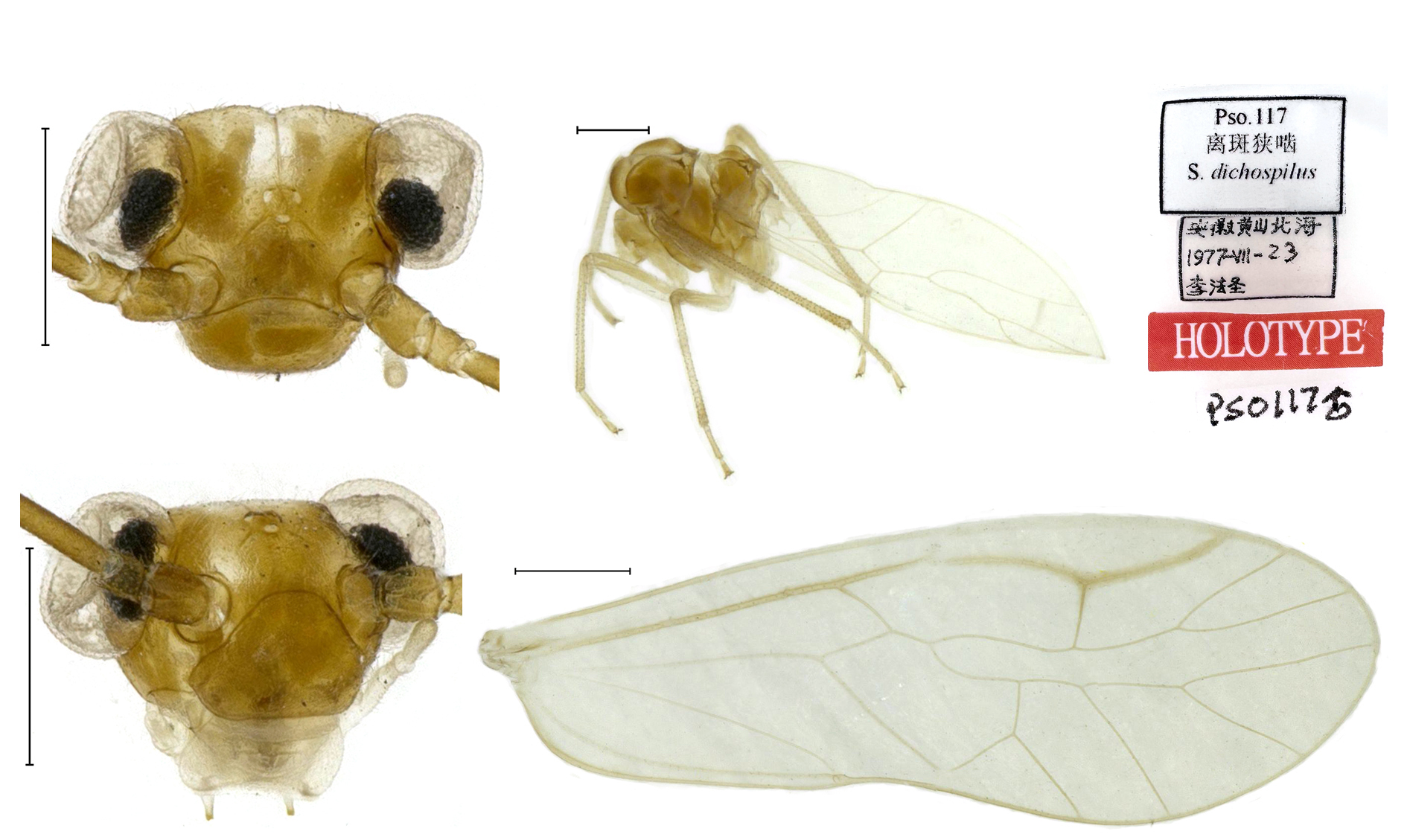

Supplement: Supplementary file 1 [file insects-16-01147-s001.zip › Figure S8 Holotype of Stenopsocus dichospilus.jpg]

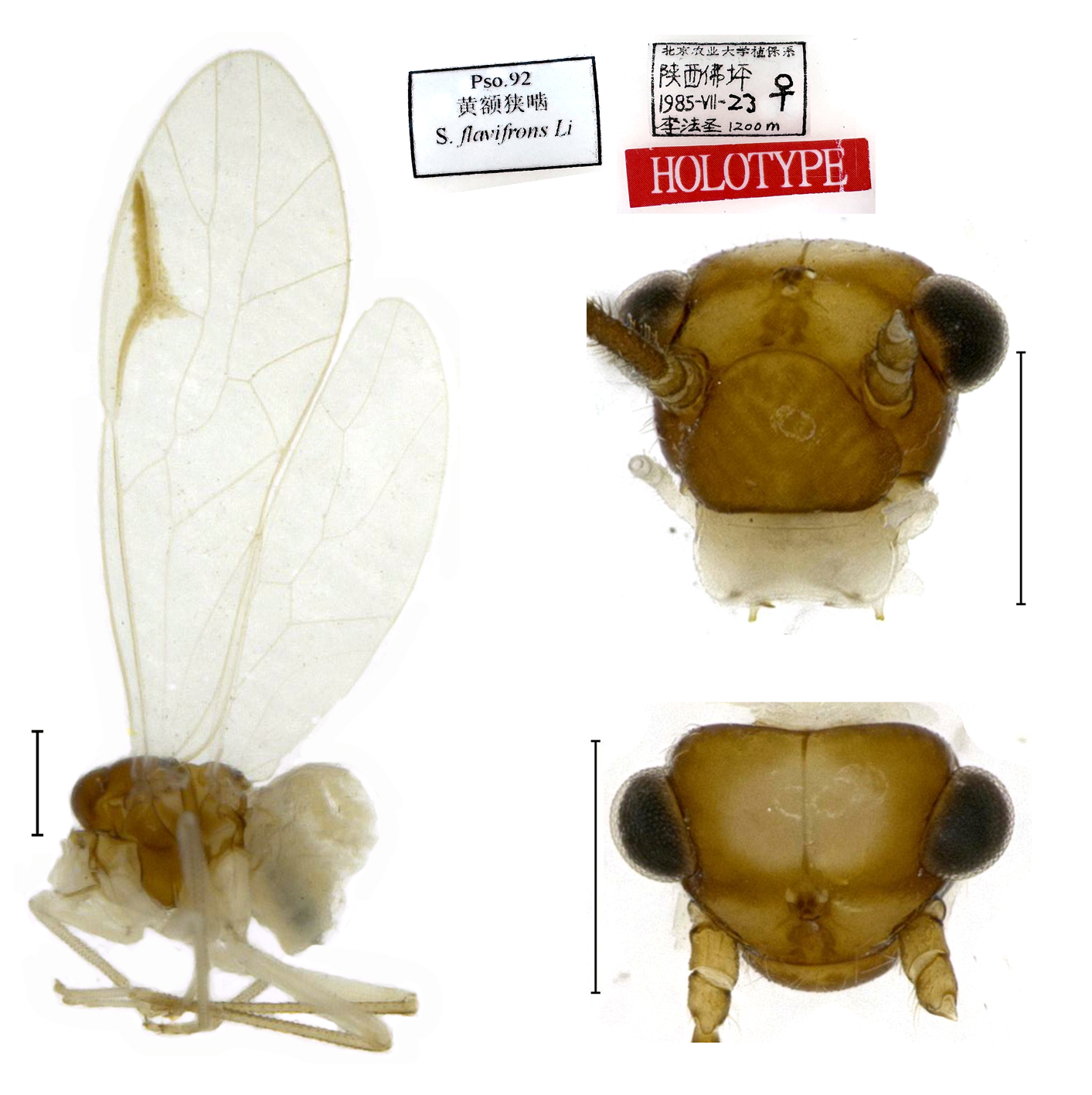

Supplement: Supplementary file 1 [file insects-16-01147-s001.zip › Figure S9 Holotype of Stenopsocus flavifrons.jpg]
